# Synthetic Glycolipids as Molecular Vaccine Adjuvants: Mechanism of Action in Human Cells and In Vivo Activity

**DOI:** 10.1021/acs.jmedchem.1c00896

**Published:** 2021-08-12

**Authors:** Fabio A. Facchini, Alberto Minotti, Andrea Luraghi, Alessio Romerio, Nicole Gotri, Alejandra Matamoros-Recio, Andrea Iannucci, Charys Palmer, Guanbo Wang, Rebecca Ingram, Sonsoles Martin-Santamaria, Grisha Pirianov, Marco De Andrea, Miguel A. Valvano, Francesco Peri

**Affiliations:** † Department of Biotechnology and Biosciences, University of Milano-Bicocca, Piazza della Scienza, 2, 20126 Milano, Italy; ‡ Department of Structural and Chemical Biology, Centro de Investigaciones Biologicas Margarita Salas, C/Ramiro de Maeztu, 9, 28040 Madrid, Spain; § Department of Translational Medicine, University of Eastern Piedmont, 28100 Novara, Italy; ∥ CAAD—Center for Translational Research on Autoimmune and Allergic Disease, University of Eastern Piedmont, 28100 Novara, Italy; ⊥ Department of Biomedical and Forensic Sciences, Anglia Ruskin University, East Road, Cambridge CB1 1PT, U.K.; # The Wellcome-Wolfson Institute for Experimental Medicine, Queen’s University of Belfast; 97 Lisburn Road, Belfast BT9 7BL, U.K.; ∇ Department of Public Health and Pediatric Sciences, University of Turin, Medical School, 10126 Turin, Italy

## Abstract

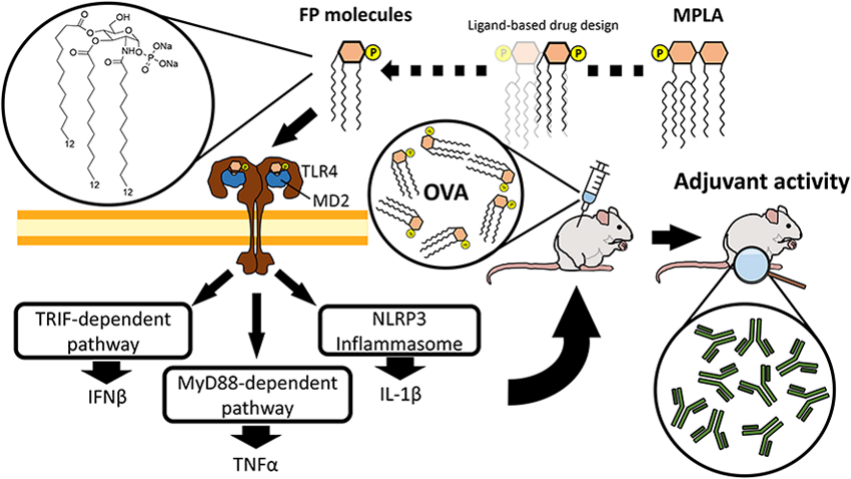

Modern adjuvants for vaccine formulations are immunostimulating agents whose action is based on the activation of pattern recognition receptors (PRRs) by well-defined ligands to boost innate and adaptive immune responses. Monophosphoryl lipid A (MPLA), a detoxified analogue of lipid A, is a clinically approved adjuvant that stimulates toll-like receptor 4 (TLR4). The synthesis of MPLA poses manufacturing and quality assessment challenges. Bridging this gap, we report here the development and preclinical testing of chemically simplified TLR4 agonists that could sustainably be produced in high purity and on a large scale. Underpinned by computational and biological experiments, we show that synthetic monosaccharide-based molecules (FP compounds) bind to the TLR4/MD-2 dimer with submicromolar affinities stabilizing the active receptor conformation. This results in the activation of MyD88- and TRIF-dependent TLR4 signaling and the NLRP3 inflammasome. FP compounds lack in vivo toxicity and exhibit adjuvant activity by stimulating antibody responses with a potency comparable to MPLA.

## Introduction

Modern subunit vaccines, based on purified or synthetic antigens that are often poorly immunogenic, require combination with adjuvants for optimal immune responses.^1^ Molecular adjuvants are single-molecule innate immune stimulants that enhance the adaptive immune response against antigens. Adjuvants activate antigen-presenting cells, such as dendritic cells and macrophages. These cells express pathogen recognition receptors that, when activated, initiate immune responses leading to the priming of T cells.^2^ Pathogen-associated molecular patterns (PAMPs), the ligands of pathogen recognition receptors, can be exploited as molecular adjuvants. Many well-defined PAMPs have been explored, most of them targeting the toll-like receptors (TLRs),^3^ C-type lectins,^4^ and nucleotide-binding oligomerization domain (NOD)-like receptors.^5^ Since TLR4 stimulation plays a key role in initiating rapid innate immune responses, TLR4 agonists are promising candidates to develop vaccine adjuvants^6−10^ and cancer immunotherapeutics.^11^ Lipopolysaccharide (LPS)-stimulated activation of TLR4 promotes the formation of the [TLR4/MD-2/LPS]_2_ membrane dimer,^12^ which interacts with two important adaptor protein molecules: MyD88 and TRIF. Signaling through the MyD88-dependent pathway results in rapid activation of NF-κB and mitogen-activated protein kinase (MAPK), both of which drive pro-inflammatory gene expression and cytokine production. Stimulation of the TRIF-dependent arm via the endocytic pathway activates interferon regulatory factors and secondary NF-κB activation,^13^ playing an important role in the stimulation of early T-cell responses.^14^ TRIF-dependent type I IFN is a central mechanism for TLR4-mediated adjuvant effects on T cell priming by TLR4 agonists.^15^ Moreover, the activation of the NLRP3 inflammasome and subsequent caspase-1 activation and release of interleukin-1β (IL-1β) is associated with the potent adjuvant effect by particulate adjuvants, such as alum, chitosan, and QuilA/saponin.^16,17^ IL-1β exerts multiple effects on the immune system,^18^ which include promoting the differentiation of Th17 cells.^19^

**Figure 1:**
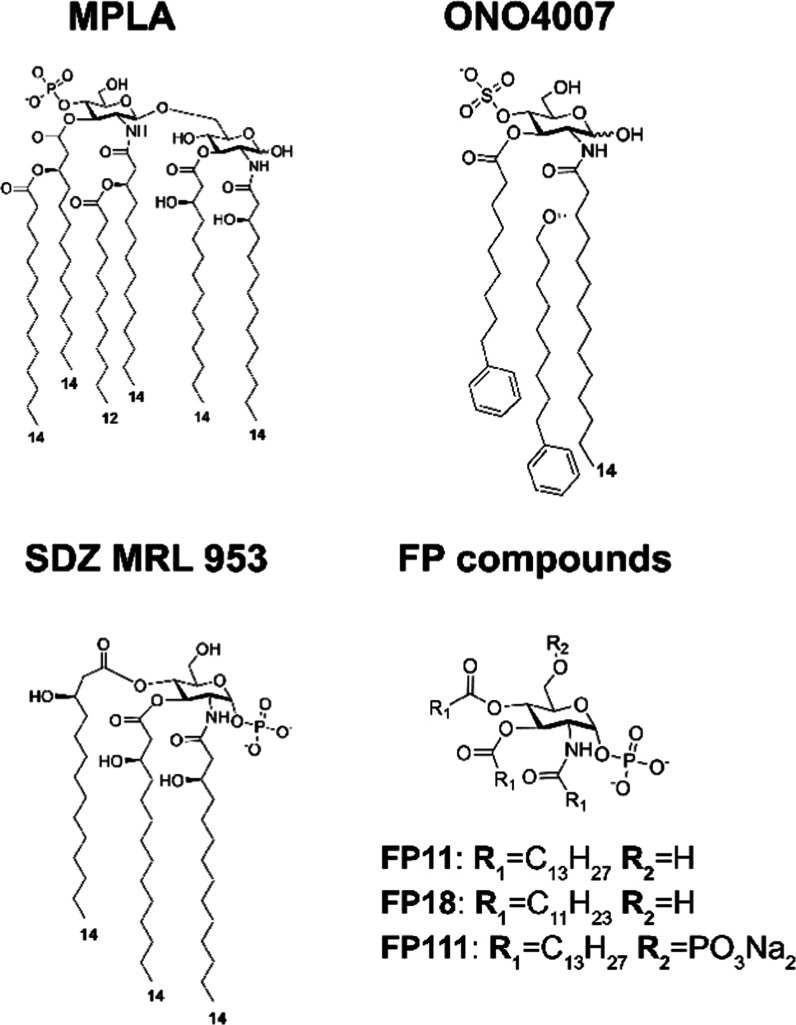
Chemical structure of TLR4 agonists: MPLA, ONO4007, SDZ MRL 953, and FP molecules.

Lipid A, the component of LPS that directly binds TLR4/MD-2 is one of the most potent immune-stimulating agents known. The toxicity of lipid A makes it unsuitable for safe use in humans but the monophosphoryl lipid A (MPLA, Figure 1),^20^ a lipid A derivative in which the anomeric phosphate has been removed, is an effective adjuvant used in various approved vaccines.^21,22^ MPLA and aminoalkyl glucosaminide phosphates (AGPs)^20^ are well-studied nontoxic TLR4 ligand adjuvants that promote Th1 (cellular)-biased immune responses. MPLA also stimulates the TLR4-mediated activation of the TRIF cascade,^14^ which also explains the reduced toxicity of MPLA.^23^ However, the production of MPLA is challenging. MPLA derived from the modification of a bacterial lipid A is not chemically homogeneous, making it difficult to assess its quality. Total synthesis of MPLA has been developed to obtain a homogeneous compound, which is named glucopyranosyl lipid A (GLA).^24−26^ However, the total synthesis of MPLA is complex (it involves around 24 chemical steps) and expensive. In contrast, easier synthetic access to monosaccharides bearing lipid chains and phosphate groups makes this class of molecules suitable to develop novel TLR4 agonists with simpler and scalable production methods. Synthetic monosaccharide mimetics of lipid X, a monosaccharide biosynthetic precursor of lipid A, were developed as TLR4 modulators. Compound SDZ MRL 953 (Figure 1) showed powerful immunostimulatory activity both in mice and humans^27^ and was tested in a phase I trial as a tumor immunotherapeutic.^28^ Also, the compound ONO 4007 (Figure 1) is a powerful immunostimulant for antitumor therapy.^29,30^ Our group developed synthetic monosaccharides, named FP compounds, which bind to the MD-2 coreceptor and block the TLR4 pathway in cells and in animal models.^31−34^ Guided by detailed structure–activity information, we predicted new compounds switching from antagonism to agonism by altering the ratio of fatty acid chains and phosphates. Compounds FP11 and FP18 (Figure 1), with a triacylated monophosphoryl glucosamine core and one phosphate group at C1, were designed and synthesized along with compound FP111, which has an additional phosphate group at C6. We present here a preclinical study on the new synthetic TLR4 agonists FP11 and FP18 (compound FP111 turning out to be inactive), their synthesis, computational studies, in vitro binding studies with the TLR4/MD-2 dimer, cell studies on the mechanism of action and TLR4 pathways activation, and in vivo assessment of their adjuvant potency, compared to FDA-approved MPLA, in an ovalbumin (OVA) vaccination model.

## Results and Discussion

### Synthesis of FP Molecules

**Scheme 1:**
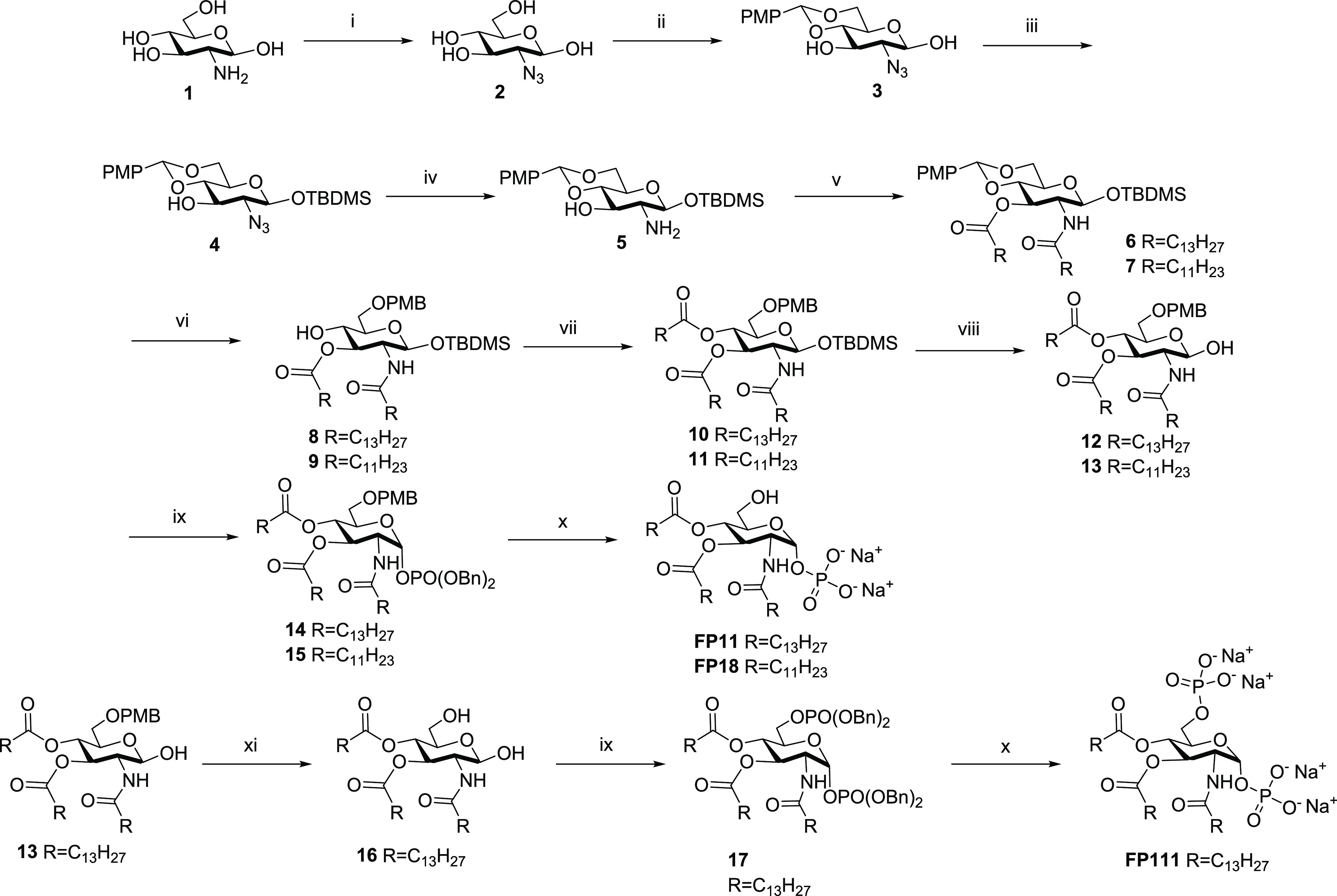
Synthesis of FP11, FP18 and FP111 Conditions: (i) NaN_3_, Tf_2_O, copper sulfate, Py, H_2_O, quant. (ii) Anisaldehyde dimethyl acetal, camphorsulfonic acid (CSA), dimethylformamide (DMF), 50 °C, 68%. (iii) TBDMSCl, imidazole, dichloromethane (DCM), 62%. (iv) PPh3, tetrahydrofuran (THF), H_2_O, quant. In the following steps, the first yield refers to FP11 synthesis and the second to FP18. (v) RCOCl, 4-dimethylaminopyridine (DMAP), triethylamine (TEA), DCM, 75%, 85%. (vi) NaBH3CN, 4 Å molecular sieves, THF, and then TFA, 83%, 85%. (vii) RCOCl, DMAP, TEA, DCM, 97%, 98%. (viii) TBAF, AcOH, THF, 87%, 90%. (ix) P(OBn)2N(*i*-Pr)_2_, imidazolium triflate, DCM and then *m*-CPBA, 50%, 55%. (x) H_2_/Pd–C, MeOH, and then IRA78-Na^+^, 80%, 87%. (xi) H_2_, Pd–C 10%, MeOH, 95%.

Compounds FP11, FP18, and FP111 were synthesized through a protocol previously developed and optimized for the preparation of TLR4 antagonists of the FP series.^32^ The synthesis for FP11 and FP18 (Scheme 1) was carried out starting from the commercially available d-glucosamine hydrochloride, following, in part, a previously published protocol consisting of the transformation of the amine at C2 into azide (**2**), followed by protection of sugar’s C4–C6 positions as *p*-methoxybenzylidene (**3**), silylation of the anomeric position (**4**), and Staudinger hydrolysis of azide into amine (**5**).^32^ Compound **5** was acylated with myristoyl chloride or lauroyl chloride to obtain, respectively, compound **6** or **7**. Regioselective opening of the *p*-methoxybenzylidene by means of sodium cyanoboronhydride (NaBH_3_CN) in trifluoroacetic acid (TFA) gave C6 *p*-methoxybenzyl (PMB)-protected compounds **8** and **9**, whose acylation with myristoyl chloride or lauroyl chloride, respectively, afforded triacylated sugars **10** and **11**. Cleavage of the anomeric silane with tetrabutylammonium fluoride (TBAF), followed by the reaction with phosphoramidite and one-pot oxidation with *m*-chloroperbenzoic acid (*m*-CPBA) gave compounds **14** and **15**. Catalytic hydrogenation, followed by the treatment with sodium cation exchange resin gave the final compounds FP11 and FP18. Similarly, FP111 was synthesized starting from the intermediate compound **13** by removal of the PMB group at C6 through catalytic hydrogenation (**16**), simultaneous phosphorylation of sugar’s C6 and C1 (**17**), and final removal of benzyl groups again by catalytic hydrogenation.

### In Vitro Binding Tests: FP11 and FP18 Bind to Human MD-2

**Figure 2:**
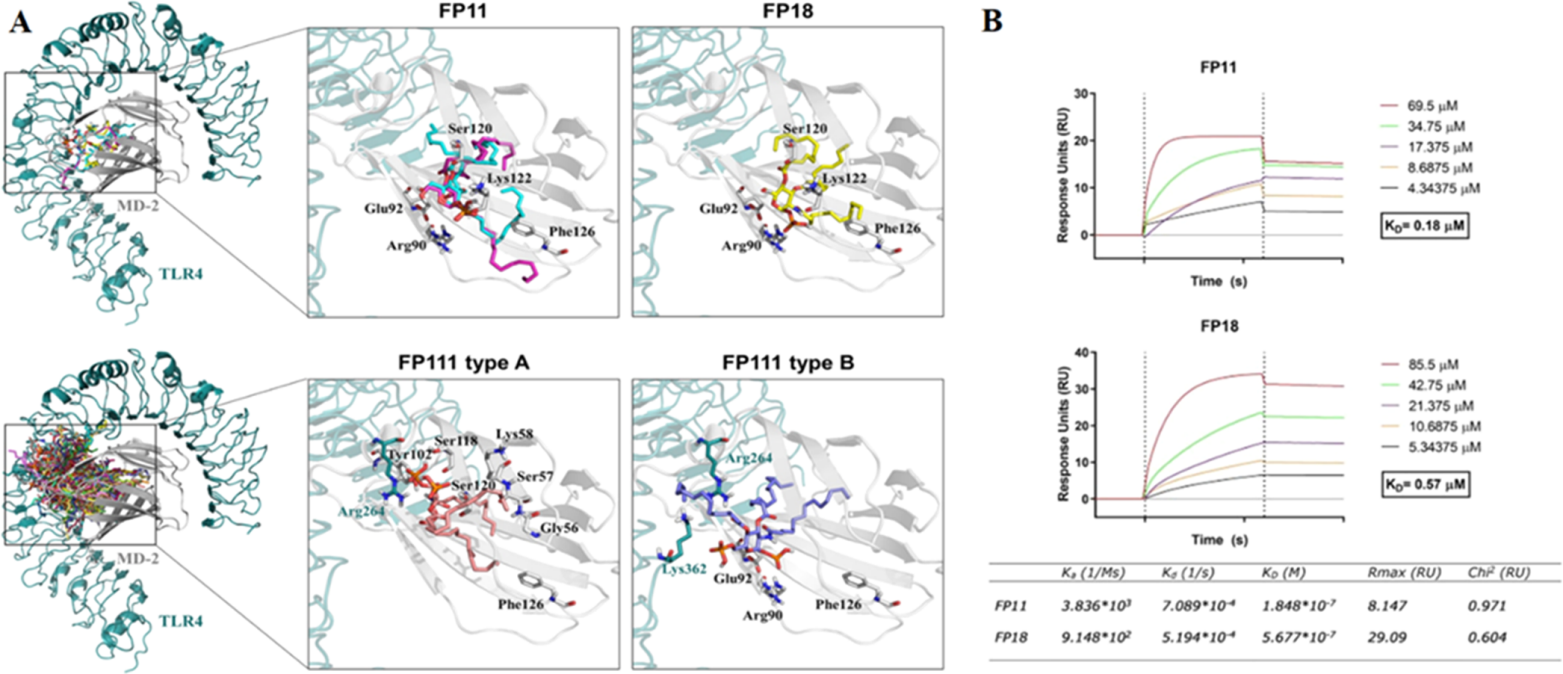
(A) Docking studies of compounds FP11, FP18, and FP111. (Top) Best AutoDock predicted binding modes for ligands FP11 and FP18 to the human TLR4/MD-2 heterodimer. On the right are details of the interactions between FP11 (magenta and cyan sticks) and FP18 (yellow sticks) and MD-2 protein. (Bottom) Binding of compound FP111 by means of the AutoDock program to the human TLR4/MD-2 system. On the right are represented detailed poses of FP111 to the TLR4/MD-2 heterodimer, with details of the interactions between FP111 type A antagonist-like (purple sticks) and type B agonist-like (salmon pink sticks) orientations. (B) Surface plasmon resonance (SPR) analyses of FP11 and FP18 binding to immobilized TLR4/MD2. Increasing concentrations of FP11 (4.34–69.50 μM, left panel) or FP18 (5.34–85.50 μM, right panel) diluted in running buffer were injected over a TLR4/MD2-immobilized NTA sensor chip. FP11 and FP18 bind to TLR4/MD2 with equilibrium dissociation constant (*K*_d_) values of 0.18 and 0.57 μM, respectively. Data are representative of three independent experiments. Binding kinetics are also shown. *K*_a_, association rate constant; M, molarity; s, seconds; *K*_d_, dissociation rate constant; *K*_D_, equilibrium dissociation constant; *R*_max_, maximum response; RU, response units; Chi_2_, average squared residual.

Direct interaction of FP11 and FP18 with human TLR4/MD-2 dimer was investigated by surface plasmon resonance (SPR) analysis. The recombinant human TLR4/MD-2 receptor complex was directly immobilized on a nitrilotriacetic (NTA) sensor chip by amine coupling and probed with increasing amounts of FP11 or FP18. The resulting SPR sensorgrams (Figure 2B) showed a direct interaction between FP molecules and the TLR4/MD-2 receptor with similar equilibrium dissociation constants (*K*_d_), 0.18 μM for FP11 and 0.57 μM for FP18. Moreover, these data indicated that both FP11 and FP18 bind to the TLR4/MD-2 receptor with fast association (*K*_a_) and slow dissociation (*K*_d_) rates, as reported for LPS and other FP molecules.^32,35^

### TLR4 Binding of FP11, FP18, and FP111: Computational Studies

To provide a three-dimensional (3D) perspective of the TLR4 binding, FP11 and FP18 were subjected to computational studies to predict their binding modes at the atomic level; FP111 was also studied. Compounds FP11, FP18, and FP111 were docked into the human TLR4/MD-2 heterodimer in the agonist activated conformation (Figure 2A, and the Supporting Information). Preliminary binding poses, obtained with AutoDock Vina, were used as starting geometries for redocking calculations with AutoDock. The calculations resulted in favorable predicted binding energies for FP11 and FP18 ligands (ranged from −3.8 to −2.7 kcal mol^–1^, for the best-ranked poses), and unfavorable binding energy for compound FP111 (values greater than 3.0 kcal mol^–1^). FP11 and FP18 showed the predicted binding poses with their fatty acid chains buried inside the MD-2 pocket, interacting with many hydrophobic and aromatic residues, and with the saccharide moiety located at the MD-2 rim, establishing polar interactions (Figure 2A). The docking calculations suggested a strong affinity and plausible binding modes for compounds FP11 and FP18 with the TLR4/MD-2 system but indicated nonefficient binding for ligand FP111 (Figure 2A). The stability of the best FP11 and FP18 predicted binding modes was confirmed by molecular dynamics (MD) simulations. Starting from the best docked TLR4/MD-2/ligand complexes, we constructed full [TLR4/MD-2/ligand]_2_ models (Figure S1) that were submitted to 50 ns MD simulations (Supporting Information). The complexes were stable during the simulations (Figure S2) and none of the ligands underwent orientation flip, all remaining in the agonist orientation predicted from docking calculations (Figure S3). Further, MD-2 Phe126 retained the agonist conformation along the MD simulations (Figure S4). We, therefore, suggest these complexes as plausible binding modes for FP11 and FP18, accounting for their agonist activity in the TLR4/MD-2 system.

### TLR4 Activation by Synthetic Agonists

The ability of FP molecules to activate human TLR4 was first assessed using HEK-Blue hTLR4 cells. These are a HEK293-derived cell line stably transfected with the LPS receptors CD14, TLR4, and MD-2 and a reporter gene, secreted embryonic alkaline phosphatase (SEAP) placed under the control of two TLR4-dependent transcription factors (NF-κB and AP-1). The HEK-Blue hTLR4 cells were treated with increasing concentrations (0.1–25 μM) of FP11, FP18, and FP111 over 18 h. Stimulation with LPS (smooth chemotype, S-LPS) served as a positive control for the activation of the TLR4-mediated pathway.

**Figure 3:**
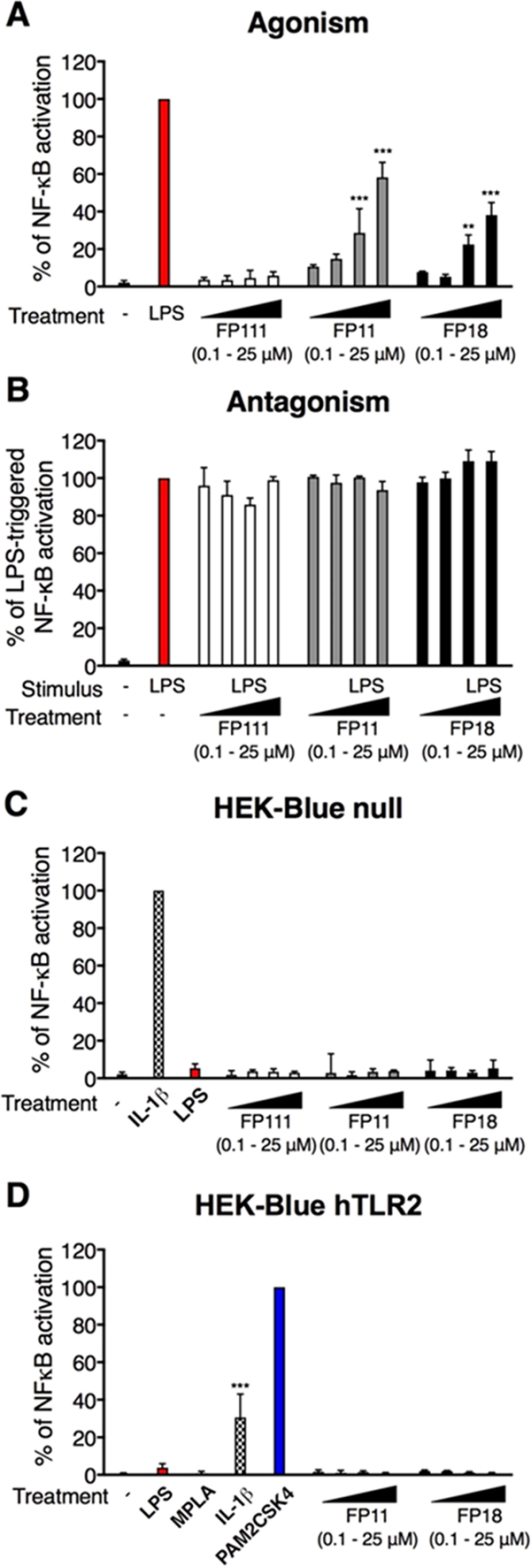
HEK-Blue hTLR4, HEK-Blue Null, and HEK-Blue hTLR2 cells were treated as indicated and incubated for 18 h. Supernatants were collected and SEAP levels were quantified by the QUANTI-blue method. Data were normalized to stimulation with S-LPS (A, B), IL-1β (C), or PAM2CSK4 (D) and expressed as the mean percentage ± standard deviation (SD) of three independent experiments (treated versus untreated (-): **p < 0.01 and ***p < 0.001).

The molecules FP11 and FP18 induced the release of the SEAP reporter protein in the medium in a concentration-dependent manner, indicating that both compounds activate NF-κB and AP-1, while FP111 was inactive (Figure 3A). The three compounds did not inhibit LPS-induced SEAP production, suggesting they lack a TLR4 antagonistic activity (Figure 3B). A lack of activity on HEK-Blue Null cells, which carry the same SEAP reporter gene but lack the LPS receptors, confirmed that both FP11 and FP18 act via TLR4 (Figure 3C). To confirm the selectivity on TLR4 over TLR2, the molecules were also tested on the HEK-Blue cells expressing hTLR2, and no agonist activity was detected (Figure 3D). These data confirm the binding data with purified TLR4/MD-2 and suggest that FP11 and FP18 are specific TLR4 agonists that directly bind to TLR4/MD-2.

### Activation of MyD88 and TRIF Pathways in Human Macrophages

**Figure 4:**
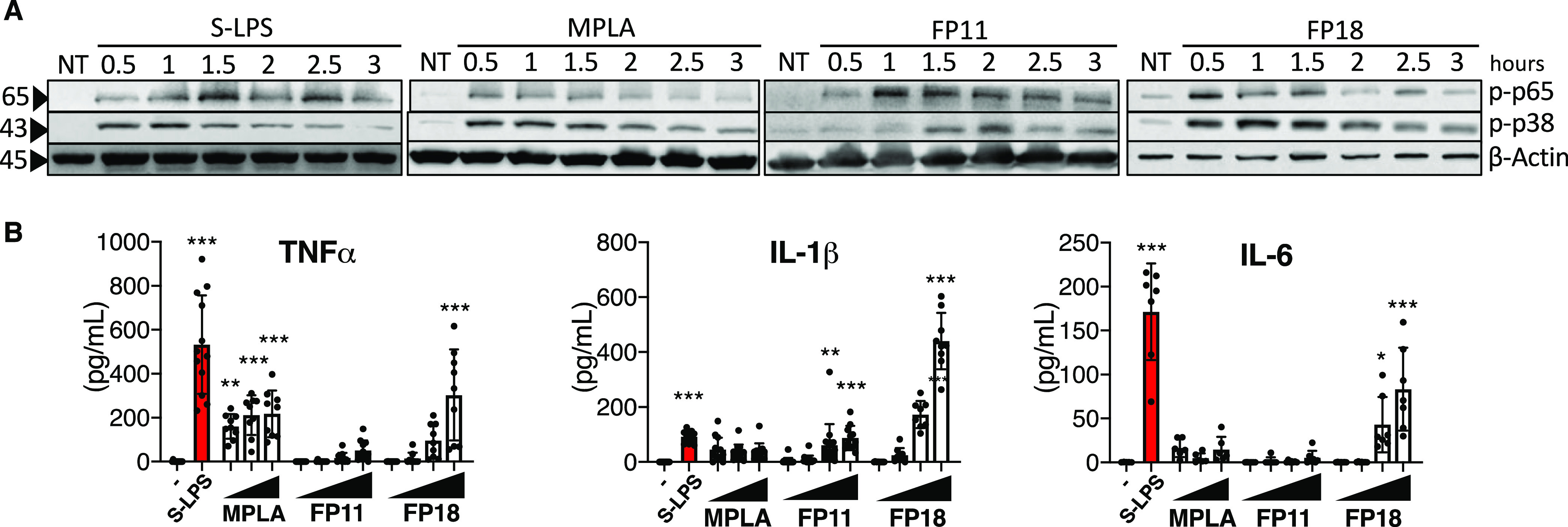
(A) TDM were treated with S-LPS (100 ng/mL), MPLA (1 μg/mL), FP11 (10 μM), FP18 (10 μM), or left untreated (NT) and collected after the indicated time. The levels of phospho-p65, phospho-p38, and actin were detected by immunoblotting. (B) In vitro cytokines released from TDM measured by enzyme-linked immunosorbent assay (ELISA). Cells were treated with S-LPS or with increasing concentrations of MPLA, FP11, and FP18 (0.1–1 to 10–25 μM) for 18 h. Error bars represent the SD of the mean. TNFα (*n* = 9), IL-1β (*n* = 9), and IL-6 (*n* = 7), where *p < 0.05, **p < 0.01, and ***p < 0.001. Statistical analysis is between treated vs untreated (-) (one-way analysis of variance (ANOVA) test).

We investigated whether the FP compounds are able to induce the same signaling pattern observed with S-LPS and MPLA stimulation in vitro. First, we evaluated whether FP11 and FP18 activate the MyD88-dependent pathway in THP-1-derived macrophages (TDM). The recruitment of MyD88 adaptor protein by the TLR4 cytosolic TIR domain promotes NF-κB and the mitogen-activated protein kinase (MAPK) activation.^36^ Therefore, the phosphorylation status of NF-κB (p65 subunit) and MAPK p38 in response to FP11, FP18, S-LPS, and MPLA treatment was assessed over time. The stimulation with S-LPS, and with FP11 and FP18, triggered p65 phosphorylation after 1 h, while for MPLA the activation of p65 occurred earlier (Figure 4A). The kinetics of p38 activation by FP11 and FP18 was similar to p65 NF-κB phosphorylation (Figure 4A), with the peak of phosphorylation after 1 h, as also observed upon S-LPS stimulation. In contrast, MPLA showed an earlier peak of phosphorylation at 30 min. The quantity of pro-inflammatory cytokines (TNFα, IL-1β, and IL-6) released following the treatment with increasing concentrations of FP11 and FP18 was compared with MPLA and S-LPS. S-LPS triggered the release of TNFα, IL-1β, and IL-6 (Figure 4B), as MPLA stimulation, albeit in reduced amounts. The effect of MPLA was dose-independent, suggesting that the concentrations used were sufficient to reach receptor saturation. FP11 treatment only triggered a dose-dependent secretion of IL-1β, while FP18 induced the production of all the three cytokines at levels exceeding those observed with MPLA stimulation (Figure 4B). These data clearly show that FP11 and FP18 activate MyD88-dependent intracellular TLR4 signaling in human macrophages.

To investigate whether FP11 and FP18 also trigger the TRIF-dependent pathway, four readouts in TDM were selected: the activation of IRF3, the expression and release of IFNβ, the activation of STAT1, and the expression of interferon-stimulated genes (ISGs). Stimulation with rough chemotype of LPS (R-LPS) was also included as reference in this part of the study.

**Figure 5:**
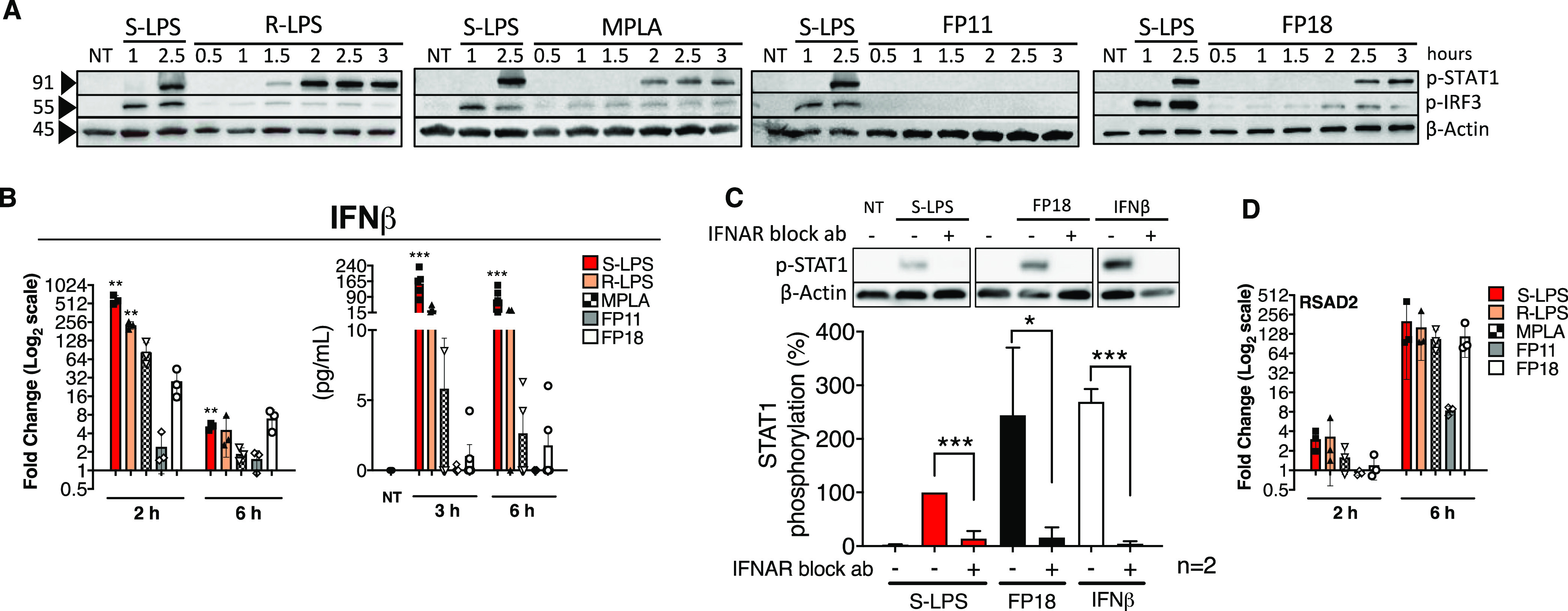
(A) TDM was treated with S-LPS (100 ng/mL), R-LPS (100 ng/mL), MPLA (1 μg/mL), FP11 (10 μM), FP18 (10 μM), or left untreated (NT) and collected after the indicated time. The levels of phospho-IRF3, phospho-STAT1, and actin were detected by immunoblotting. (B) TDM were treated as in panel A and collected after the indicated time. (Left) IFNβ production is expressed as fold change compared to untreated cells. Error bars represent SD of the mean; *n* = 3, where *p < 0.05, **p < 0.01, and ***p < 0.001. Statistical analysis is between treated vs untreated (Wilcoxon’s test). (Right) IFNβ release was quantified by ELISA. Error bars represent SD of the mean; *n* = 4 where *p < 0.05; **p < 0.01; and ***p < 0.001. Statistical analysis is between treated vs untreated (NT) (one-way ANOVA test). (C) TDM were pretreated with IFNAR2 neutralizing antibody (1 μg/mL) 1 h prior to exposure to the indicated stimulus. Cell lysates were collected after 3 h and p-STAT1 was measured via immunoblotting. Error bars represent SD of the mean; *n* = 2 where *p < 0.05 and ***p < 0.001 (one-way ANOVA test). (D) TDM were treated as in panel B and collected after the indicated time. RSAD2 expression is expressed as fold change compared to untreated cells. Error bars represent SD of the mean; *n* = 3, where *p < 0.05, **p < 0.01, and ***p < 0.001. Statistical analysis is between treated vs untreated (Wilcoxon’s test).

S-LPS strongly induced IRF3 phosphorylation 1 h after treatment but the effect of FP18 was delayed, with a peak of IRF3 activation observed after 2.5 h (Figure 5A). The peak of IRF3 phosphorylation following R-LPS and MPLA stimulation was of a similar magnitude to that induced by FP18, appearing between 1 and 1.5 h post-treatment (Figure 5A). In contrast, FP11 did not induce IRF3 phosphorylation (Figure 5A). Next, we evaluated whether IFNβ induction followed IRF3 activation by examining gene expression and cytokine release. Consistent with previous results, increased IFNβ transcription was observed 2 h poststimulation with S-LPS, R-LPS, and MPLA in comparison with untreated cells, with 600-, 250- and 60-fold increase, respectively, followed by a rapid decline at 6 h post-treatment (Figure 5B, left panel). FP18 showed limited ability to induce the IFNβ mRNA expression, with a 20-fold increase at 2 h post-treatment in comparison with untreated cells. In agreement with the IRF3 phosphorylation status, IFNβ mRNA expression was unaffected by FP11 stimulation. The levels of IFNβ secreted corroborated the mRNA results, whereby significantly higher levels were detected in the culture supernatants of cells treated with S-LPS and R-LPS than from untreated cells at both 3 and 6 h post-treatment (Figure 5B, right panel). In contrast, induction of IFNβ was lower in MPLA- and FP18-treated cells, and not observed with FP11 stimulation. Despite the diminished induction of IFNβ, downstream STAT1 phosphorylation was detected starting from 2.5 h upon FP18 and S-LPS stimulation, indicating that IFNβ release mediated by FP18 was sufficient to induce ISGs. By comparison, R-LPS and MPLA triggered STAT1 phosphorylation after 1.5 and 2 h upon stimulation, respectively (Figure 5A). The phosphorylation of STAT1 due to IFNβ binding to its receptor was confirmed using a specific blocking antibody that prevents IFNAR activation. As predicted, S-LPS and FP18 exposure or direct exogenous IFNβ stimulation triggered a significant increase in STAT1 phosphorylation (Figure 5C), which was completely abolished by blocking IFNAR in all of the three cases, indicating that FP18 can induce type I IFNs release (Figure 5C). Finally, STAT1-mediated expression of an ISG (namely, Viperin/RSAD2) was also evaluated. S-LPS induced Viperin/RSAD2 gene expression, reaching a maximum peak 6 h post-treatment (Figure 5D). A similar pattern of activation was observed for FP18, R-LPS, and MPLA treatments causing increased RSAD2 expression after 6 h, with a potency similar to S-LPS. In contrast, very low RSAD2 transcription was detected in cells stimulated with FP11 (Figure 5D). These results suggest that FP18 triggers both the MyD88- and the TRIF-dependent pathways, while FP11 preferentially activates the MyD88 pathway.

### FP18 Induces Caspase-1 Activation and Release of Mature IL-1β in a NLRP3-Dependent Manner

IL-1β is the predominant pro-inflammatory cytokine induced by FP molecules and its release is normally associated with the activation of NLRP3 inflammasome. The ability of FP11 and FP18 to trigger caspase-1 activation and IL-1β maturation was investigated using TDM. We first analyzed IL-1β production by a cell-associated ELISA, which allowed us to compare precursor and mature forms of the cytokine in cell lysates and supernatants, respectively.

**Figure 6:**
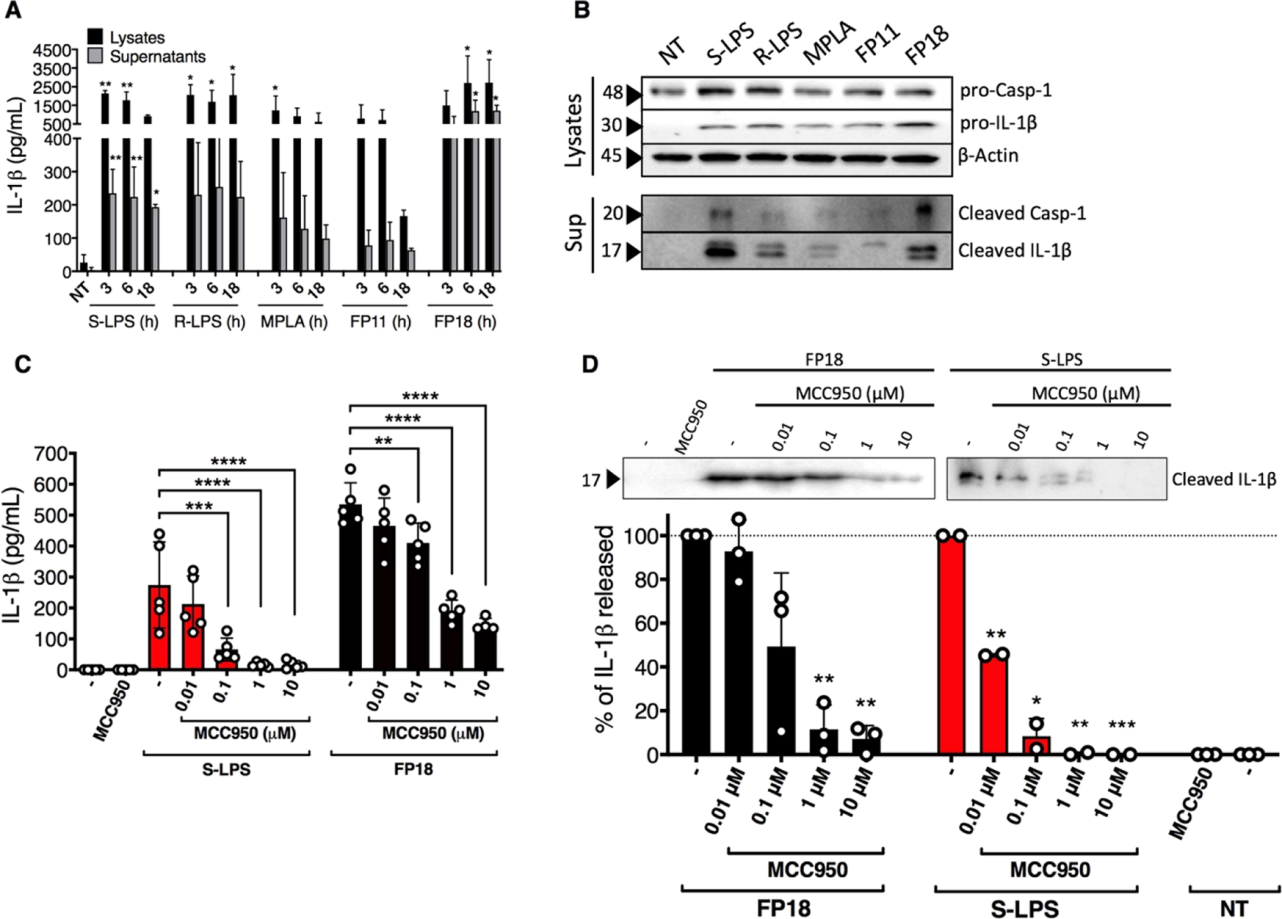
(A) TDM were treated with S-LPS (100 ng/mL), R-LPS (100 ng/mL), MPLA (1 μg/mL), FP11 (20 μM), FP18 (20 μM), or left untreated. IL-1β levels were measured in both cell lysates and supernatants by ELISA. Error bars represent SD of the mean; *n* = 3 where *p < 0.05, **p < 0.01, and ***p < 0.001. Statistical analysis is between treated vs untreated (NT) (one-way ANOVA test). (B) TDM were treated as in panel A for 6 h. Levels of pro-casp-1/pro-IL-1β, and cleaved casp-1/cleaved-IL-1β were detected by immunoblot in cell lysates and supernatants, respectively. (C, D) TDM were pretreated for 1 h with an increasing concentration of MCC950 and then stimulated with S-LPS (100 ng/mL) or FP18 (20 μM) for 6 h. The effect of MCC950 on LPS- and FP18-triggered IL-1β secretion was evaluated by ELISA (C) and immunoblot (D). In (C), error bars represent SD of the mean; *n* = 5 where *p < 0.05, **p < 0.01, and ***p < 0.001 (one-way ANOVA test). In (D), the percentage of IL-1β release was normalized to stimulation with only S-LPS or only FP18, and error bars represent SD of the mean (*p < 0.05, **p < 0.01, and ***p < 0.001, Wilcoxon’s test).

The accumulation of IL-1β precursor in cell lysates was induced by FP11 and FP18, as well as the other treatments tested, after 3, 6, and 18 h (Figure 6A). This induction was, however, only significant in the FP18-, S-LPS- and R-LPS-treated cells. Similarly, the analysis of supernatants revealed that although IL-1β release was triggered by all molecules, only FP18 and S-LPS caused significant secretion of the IL-1β mature form. The lower level of activity of FP11 is consistent with the previous results (Figure 6A). Moreover, IL-1β levels were higher in cell lysates, suggesting a partial activation of the inflammasome with a limited release of the mature cytokine. For this reason, we evaluated the canonical inflammasome activation by monitoring caspase-1 activation and mature IL-1β release in the extracellular compartment through western blotting. Caspase-1 was constitutively expressed in differentiated THP-1 cells, while, in agreement with the cell-associated ELISA assay, the IL-1β precursor was induced after 6 h by all compounds (Figure 6B). It was also demonstrated that only FP18 and S-LPS induced high levels of caspase-1 cleavage and IL-1β maturation and release, while MPLA and R-LPS stimulation resulted in weaker signaling (Figure 6B). To evaluate NLRP3 contribution in FP18-triggered IL-1β release, TDM were pretreated for 1 h with increasing concentration of the NLRP3 inhibitor MCC950 (0.01–10 μM) and then stimulated with FP18 or S-LPS for 6 h. After treatment, cell supernatants were checked for IL-1β levels by ELISA assay and western blot. MCC950 pretreatment significantly inhibited IL-1β release, in a concentration-dependent manner, in both FP18- and S-LPS-treated cells (Figure 6C,D). Collectively, these data demonstrated that FP18 triggers NLRP3 canonical inflammasome inducing caspase-1 activation and IL-1β release.

### Adjuvant Activity of FP11 and FP18 and In Vivo Toxicity: OVA Immunization Experiments

**Figure 7:**
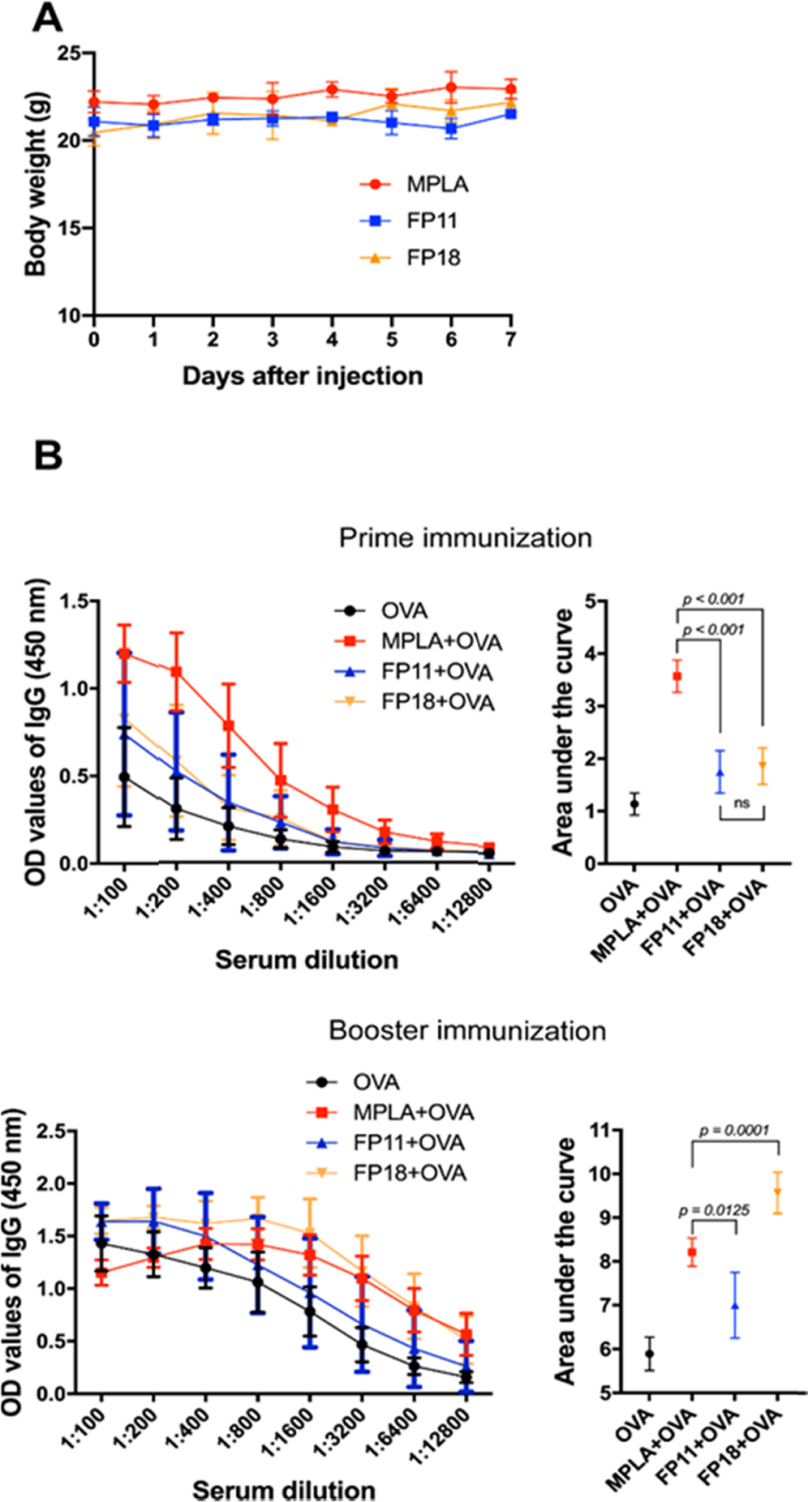
(A) Body weight of mice over 7 days after administration of adjuvants (*n* = 4 per treatment). (B) Antibody response to OVA immunization using MPLA, FP18, and FP11 as adjuvants after prime (22 days postimmunization) and booster immunization (19 days later) (*n* = 8 per treatment). For statistical comparisons, the area under each curve was examined by Brown–Forsythe and Welch one-way ANOVA tests with an α of 0.05.

The ability of FP11 and FP18 to induce immune responses in vivo was compared to MPLA by evaluating antibody production in C57Bl/6 mice immunized with chicken ovalbumin (OVA) as a model antigen. We first evaluated the toxicity of FPs in a pilot experiment in which mice were injected subcutaneously with 10 μg of FP11 and FP18. The results showed that the two test adjuvants had no obvious adverse effect on mice, as assessed by the local response at the injection site and by determining the animal weight and state of alertness over 7 days (Figure 7A). Next, mice were immunized with the tested adjuvants mixed with ovalbumin (OVA). The induction of antibody was evaluated 21 days postimmunization. The results showed that mice immunized with the test adjuvants exhibited marginally higher levels of anti-OVA total IgG after prime immunization compared to OVA-immunized control and significantly lower levels compared to MPLA-OVA-immunized animals (Figure 7B, prime immunization). In contrast, after a boost immunization given on day 22 and examined for ova-specific antibody titers 14 days later, the IgG levels in the FP18-immunized mice were higher than those in the FP11-immunized group (Figure 7B, booster immunization). These data indicate that, in agreement with in vitro and in cell results, FP18 is a more effective adjuvant in vivo than FP11 and has a potency comparable or even greater than MPLA.

## Conclusions

By engaging the TLR4/MD2 endotoxin receptor system, natural adjuvants such as LPS and the LPS-derived MPLA modulate adaptive immune responses by influencing early T-cell clonal expansion and the cytokine milieu expressed during antigen-dependent proliferation. In addition, inflammasome activation has been associated with the adjuvant efficiency of the first clinically approved combination adjuvants, AS01, and AS04, which contain MPLA, the saponin QS-21, alum,^17,37^ and the FDA-approved squalene-based oil-in-water emulsion adjuvants MF59 and AS03.^1,38,39^ AS04 and AS01 were used in U.S. vaccine (Cervarix) and in the recombinant Varicella zoster vaccine Shingrix, respectively. Despite showing reassuring overall safety profiles in both vaccines, the lack of chemical homogeneity of MPLA derived from chemical modification of bacterial LPS poses manufacturing and quality control challenges at the industrial level, potentially limiting its use. On the other hand, the chemical synthesis of MPLA, consisting of 24 steps, is expensive and its upscale is challenging. Druggable, chemically simplified MPLA substitutes, such as monosaccharide-based TLR4 agonists, could be more attractive compounds to develop adjuvants that can be sustainably produced on a global scale. Therefore, we are working toward the development of new glucosamine derivatives with phosphate at the α-anomeric position and three lipid chains linked at positions C2, C3, and C4 (termed FP compounds) as TLR4/MD-2 ligands. FP compounds are similar to synthetic agonist SDZ MRL 953, except for the absence of C3 hydroxyl groups on the three fatty acid chains. However, the retention of biological activity after deletion of C-3 is not obvious, and it generally causes reduced activity, as observed with other lipid A derivatives.^40^ The presence of nonhydroxylated fatty acid chains greatly simplifies FP chemical synthesis, compared to MPLA or other monosaccharide lipid A mimetics, making these compounds scalable at an industrial level. Compounds FP11 and FP18 differ only in the length of the fatty acid chains (14 and 12 carbons, respectively). We show here that both compounds have favorable predicted binding energies when docked to the activated TLR4/MD-2 system. Compound FP111, with a second phosphate on sugar’s C6, showed unfavorable predicted binding energy. This behavior can be explained by the presence of two phosphates in 1,6-positions that do not allow an LPS-like binding mode at the MD-2 rim, unlike the mono (1- or 6-) phosphate pattern. FP111 binding poses are predicted to be anchored through one phosphate group to MD-2 and the second phosphate to the TLR4 chain instead of being completely inserted into the MD-2, accounting for the observed lack of activity. Experiments to assess the ability of the three molecules to trigger TLR4 activation in HEK-Blue cells confirmed that FP11 and FP18 behave as selective TLR4 agonists, and are not active on TLR2, while the FP111 control molecule was inactive. FP11 and FP18 bound TLR4 at sub-micromolar affinities and are both capable to stimulate the MyD88-dependent pathway in human THP-1 cells, ultimately leading to the release of TNFα, IL-1β, and IL-6 cytokines, albeit to different extents. FP18 showed a higher potency than FP11 and MPLA in inducing IL-1β release due to a greater ability to activating the +NLRP3 inflammasome. This activity resembled that of S-LPS, a known potent inflammasome inducer. However, unlike S-LPS, the NLRP3-specific inhibitor MCC950 only partially reversed the efficacy of FP18, as shown by the amount of IL-1β still released even at the higher concentration of the inhibitor, suggesting this compound causes. In addition, results obtained showed that FP18 stimulatory activity is not limited to the activation of the MyD88-dependent pathway, but also involves activation of the TRIF-dependent pathway leading to a type I IFN signature.^15^ The stronger overall activity of FP18 over FP11 is consistent with the higher polarity and solubility in aqueous media of FP18 (calculated log *P* = 8.3 and 10.2 for FP11 and FP18, respectively, see Table S2), explaining higher efficacy when using this molecule in an aqueous environment. The FP18 and FP11 differences in inflammasome activation were also mirrored in their relative activities as immunization adjuvants. Although both molecules had adjuvant effects in vivo, FP18 was significantly more potent than FP11 and also displayed greater potency than MPLA upon boost immunization. In conclusion, we have demonstrated that synthetic FP11 and FP18 show in vivo immunostimulatory activity with a potency similar to MPLA, and established the molecular mechanisms explaining their action based on the selective TLR4 stimulation with activation of MyD88- and TRIF-dependent pathways and inflammasome. These observations, together with the lack of obvious in vivo and in vitro (Figure S18, Supporting Information) toxicity, and the straightforward synthesis procedure compared to MPLA, support preclinical, and clinical development of FP molecules as novel lead compounds for the production of effective vaccine adjuvants.

## Experimental Section

### General

All reagents and solvents were purchased from commercial sources and used without further purifications unless stated otherwise. Reactions were carried out under a nitrogen atmosphere unless otherwise noted and were monitored by thin-layer chromatography (TLC) performed over Silica Gel 60 F254 plates (Merck) and revealed using UV light or staining reagents (H_2_SO_4_ 5% in EtOH), ninhydrin (5% in EtOH), basic solution of KMnO_4_ (0.75% in H_2_O), molybdate solution (molybdate phosphorus acid and Ce(IV) sulfate in 4% H_2_SO_4_). Flash chromatography purifications were performed on silica gel 60 (40–63 μm) from commercial sources. ^1^H and ^13^C NMR spectra of compounds were recorded with a Bruker Advance 400 with TopSpin software, or with an NMR Varian 400 with Vnmrj software. Chemical shifts are reported in parts per million (ppm) relative to the residual solvent; coupling constants are expressed in Hz. The multiplicity in the ^13^C spectra was deduced by attached proton test (APT) pulse sequence; peaks were assigned with the help of 2D-COSY and 2D-HSQC experiments. Exact masses were recorded with Orbitrap Fusion Tribrid. Purity of final compounds was about 95% as assessed by quantitative NMR analysis. Reaction conditions and compound characterization are described in the Supporting Information.

### Surface Plasmon Resonance Analysis

Real-time binding interaction experiments were performed with a Biacore X100 (GE Healthcare). Recombinant human TLR4/MD2 complex was covalently immobilized onto the surface of a sensor chip NTA (cat # BR100034, GE Healthcare) via amine coupling. TLR4/MD2 complex was diluted to a concentration of 20 μg/mL in 10 mM sodium acetate at pH 4.0, and was injected on the NTA chip at a flow rate of 10 μL/min, upon washing with 0.35 M ethylenediaminetetraacetic acid (EDTA), injection of NiCl_2_ for 60 s, a second wash with 3 mM EDTA, and activation of the carboxyl groups on the sensor surface with 7 min injection of a mixture of 0.2 M EDC and 0.05 M NHS. The remaining esters were blocked with 7 min injection of ethanolamine. Based on the ligand (TLR4/MD2) and analytes (FP11, FP18), molecular weights (MW) of 90 kDa, and 934.39 or 850.04 Da, respectively, the appropriate ligand density (RL) on the chip was calculated as follows: RL = (ligand MW/analyte MW) × *R*_max_ × (1/*S*_m_), where *R*_max_ is the maximum binding signal and *S*_m_ corresponds to the binding stoichiometry. The target capture level of the TLR4/MD2 complex was 1933.5 response units (RUs). The other flow cell was used as a reference and was immediately blocked after the activation. Increasing concentrations of FP11 or FP18 were flowed over the NTA sensor chip coated with TLR4/MD2 at a flow rate of 30 μL/min at 25 °C with an association time of 60 s and a dissociation phase of 180 s. A single regeneration step with 50 mM NaOH and an extra wash with phosphate buffered saline (PBS-P) + with 50% dimethyl sulfoxide (DMSO) were performed following each analytic cycle. All the analytes tested were sonicated for 15 min and then diluted in the PBS-P + buffer (GE Healthcare) with 5% DMSO. The *K*_D_ values were evaluated using the Biacore evaluation software (GE Healthcare) after solvent correction, and the reliability of the kinetic constants calculated by assuming a 1:1 binding model supported by the quality assessment indicators values.

### Cell Cultures

HEK-Blue cells and RAW-Blue Cells (InvivoGen) were cultured according to the supplier’s instructions. Briefly, cells were cultured in Dulbecco’s modified Eagle’s medium (DMEM) high glucose medium supplemented with 10% heat-inactivated fetal bovine serum (FBS), 2 mM glutamine, penicillin (100 U/mL), streptomycin (100 U/mL), and supplemented with the antibiotics indicated in Table S1. Cells were detached using a cell scraper, counted, and seeded in a 96-well multiwell plate at a density indicated in Table S1. After overnight incubation (37 °C, 5% CO_2_, 95% humidity), supernatants were removed, and cell monolayers washed with warm PBS. Cells were resuspended in a fresh medium supplemented with the molecules to be tested and incubated for 18 h. THP-1 cells were cultured in the Roswell Park Memorial Institute Medium (RPMI) supplemented with 10% heat-inactivated FBS, 2 mM glutamine, penicillin (100 U/mL), and streptomycin (100 U/mL). Cells were split three times weekly and maintained at a density of 0.5 × 10^6^ cells/mL. For experimental procedures, THP-1 were seeded in multiwell plates at a density of 0.5 × 10^6^ cells/mL, 200 μL/well (96 wells), 1.5 mL/well (12 wells), and 3 mL/well (6 wells), and differentiated into macrophages with phorbol 12-myristate 13-acetate (PMA, Invivogen) at a final concentration of 25 ng/mL. After 72 h of differentiation, the culture medium was replaced with a fresh medium, and cells were rested for another 24 h before exposure to the molecules to be tested.

### Cell Stimulation and Treatments

All LPS variants were purchased from Innaxon. Unless otherwise indicated, cells were stimulated with 100 ng/mL ultrapure Smooth-LPS from *Salmonella minnesota* (S-LPS) for 18 h. Rough-LPS R595 (Re) from *S. minnesota* (R-LPS) was used at 100 ng/mL, while MPLA R595 (Re) from *S. minnesota* (MPLA) was used at 1 μg/mL. For TLR2 activation, PAM2CSK4 (Invivogen) was added at 10 ng/mL for 18 h. hIL-1β (Merk) was used as control for NF-κB activation and added at a final concentration of 100 ng/mL. FP11, FP111, and FP18 compounds were resuspended in ultrapure DMSO and diluted in culture medium. Anti-human IFNAR2 neutralizing antibody (clone MMHAR-20) was purchased from PBL Assay Science and used at 1 μg/mL. The NLRP3 inhibitor MCC950 was purchased from Merck and added to cells at the following concentrations: 0.01, 0.1, 1, and 10 μM.

### HEK-Blue Cells Reporter Assay

After the addition of the molecules to be tested, cells were incubated for 18 h. Supernatants were collected and SEAP levels were quantified using QUANTI-Blue assay according to the manufacturer’s instruction. Briefly, 20 μL of the supernatants of SEAP-expressing cells was incubated with 200 μL of QUANTI-Blue substrate in a 96-well plate for 0.5–4 h at room temperature (RT). SEAP activity, as an indicator of TLR4 activation, was assessed reading the well’s optical density (OD) at 630 nm. The results were normalized with positive control (Smooth-LPS for HEK-Blue hTLR4 cells, PAM2CSK4 for HEK-Blue hTLR2 cells, and IL-1β for HEK-Blue Null cells) and expressed as the mean of percentage ± standard error of the mean (SEM) of at least three independent experiments.

### RNA Extraction, cDNA Synthesis, and Real-Time Polymerase Chain Reaction

Total RNA was extracted using Quick-RNA MiniPrep (Zymo Research) according to the manufacturer’s instruction. Reverse transcription was performed with 1 μg of total RNA using LunaScript RT SuperMix Kit (New England BioLabs, MA), and cDNA was amplified using the Luna Universal qPCR Master Mix (New England BioLabs, MA) under the following conditions: denaturation for 1 min at 95 °C; annealing for 30 s at 62 °C for human IFNβ, 58 °C for human IL-1β, 60 °C for human RSAD2, and 60 °C for human β-actin; and 30 s of extension at 72 °C. Primer sequences were as follows: human IFNβ forward 5′-CAACTTGCTTGGATTCCTACAAAG-3′ reverse 5′-GTATTCAAGCCTCCCATTCAATTG-3′; human IL-1β forward 5′-AGAATGACCTGAGCACCTTC-3′, reverse 5′-GCACATAAGCCTCGTTATCC-3′; human RSAD2 forward 5′-AGAATACCTGGGCAAGTTGG-3′, reverse 5′-GTCACAGGAGATAGCGAGAATG-3′; β-actin (forward 5′-AAGATGACCCAGATCATGTTTGAGACC-3′, reverse 5′-AGCCAGTCCAGACGCAGGAT-3′) was used as a housekeeping gene. Gene expression was calculated using the ΔΔ*C*_t_ function and expressed as fold change compared to not treated cells.

### Enzyme-Linked Immunosorbent Assay (ELISA)

TNF-α, IL-1β, IL-6, and IFNβ levels were measured in TDP supernatants and cell lysates after the indicated timing using the following sensitive enzyme-linked immunosorbent assays (ELISA) (R&D Systems; #DY210-05, #DY201-05, #DY200-05, #DY206-05, #DY208-05, #DY814-05 Minneapolis). The optical density of each well was determined using a microplate reader set to 450 nm (wavelength correction: 570 nm).

### Western Blot Analysis

Immunoblotting of caspase-1 and mature IL-1β from precipitated supernatant was performed as described.^41^ For cell extracts, cells were washed twice in ice-cold PBS and lysed in radioimmunoprecipitation assay buffer (RIPA) buffer (CST, #9806), supplemented with protease (Roche, Mannheim, Germany) and phosphatase inhibitors (CST ##5870). After centrifugation at 13 000 RCF for 30 min at 4 °C, the supernatants were collected as whole cell lysates. Methanol/chloroform precipitated cell supernatants and cell lysates were resuspended in the Laemmli buffer, denatured for 5 min at 100 °C, and separated on 10 or 13% polyacrylamide gels. Proteins were transferred on poly(vinylidene difluoride) (PVDF) filters (Bio-Rad), blocked in 5% w/v BSA TTBS, and incubated with the primary and corresponding secondary antibodies indicated below. Proteins were revealed by chemiluminescence (LiteAblot EXTEND, Euroclone) and detected using Odyssey Fc LI-COR Imaging System. The PVDF membrane filters were incubated with the following primary antibodies: anti-phospho NF-κB (Ser536) (93H1) rabbit mAb (CST #3033; diluted 1:1000); anti-phospho-p38 MAPK (Thr180/Tyr182) (D3F9) XP rabbit mAb (CST #4511; diluted 1:1000); anti-phospho-IRF-3 (Ser386) (E7J8G) XP rabbit mAb (CST #37829 diluted 1:1000); anti-phospho-STAT1 (Tyr701) (58D6) rabbit mAb (CST #9167 diluted 1:1000); anti-IL-1β (3A6) mouse mAb (CST #12242 diluted 1:1000); anti-cleaved-IL-1β (Asp116) (D3A3Z) rabbit mAb (CST #83186 diluted 1:1000); caspase-1 (D7F10) Rabbit mAb (CST #3866 diluted 1:1000); and anti-β-actin (13E5) rabbit mAb (CST #4970 diluted 1:1000). Secondary antibodies used were anti-rabbit or anti-mouse IgG and HRP-linked secondary antibody (Cell Signaling #7074 and #7076, diluted 1:3000). Densitometric analysis was carried out using Image J.

### Mice Immunization Experiments

The in vivo protocols were reviewed by the Queen’s University Animal Welfare and Ethical Review Body (AWERB), and the work was carried out under an approved UK Home Office Project License (PPL2807). Chicken ovalbumin (OVA, Sigma-Aldrich) was resuspended in pyrogen-free Dulbecco’s phosphate-buffered saline (DPBS) (Sigma-Life Science) at 5 mg/mL. Endotoxins were removed by Pierce High-Capacity Endotoxin Removal Spin Columns (Thermo Scientific). The endotoxin level of purified OVA was determined by the Limulus Amebocyte Lysate (LAL) Gel-clot method (Associates of Cape Cod; East Falmouth, MA) in the form of single test vials. The samples were assessed at a sensitivity of 0.125 endotoxin unit (EU)/mL. OVA concentration was determined by BioRad Protein Assay Dye Reagent (Bio Rad) and bovine serum albumin (BSA, Sigma-Aldrich) as a reference standard. Six-week-old female C57BL/6 mice were purchased from Envigo, U.K. For the pilot toxicity experiment, mice (*n* = 3 per treatment) were injected subcutaneously on the flank with 10 μg of adjuvants (FP11, FP18, or MPLA) suspended in 50 μL of PBS. The mice were monitored and weighed daily for 7 days. For immunization, C57BL/6 mice (*n* = 8 per treatment) were immunized by subcutaneous injection on the flank with 50 μL of 500 μg of OVA mixed with 10 μg of FP11, FP18, or MPLA resuspended in PBS. A control group of OVA without the adjuvant was also included. Mice were given a booster immunization on the alternative flank 22 days after prime immunization. Serum, obtained from blood samples drawn from the tail vein, was examined for anti-OVA antibodies at days 21 and 41. The antibody levels in sera were measured by ELISA. Wells of polystyrene microplates Nunc Maxisorp (Thermo Scientific) were coated with 50 μL of OVA at 4 μg/mL diluted in 50 mM carbonate/bicarbonate buffer, pH 9.6, at 4 °C overnight. The coating solution was removed, and plates were washed with 300 μL of PBS/Tween 20 (0.05%). Additional blocking was achieved by adding 200 μL of blocking buffer (BSA 5%). Plates were covered and incubated at room temperature for 1 h and then washed three times with PBS/Tween20. Fifty microliters of serum diluted in half-strength blocking buffer (from 1:100 to 1:12 800) was added to the wells and incubated for 90 min at room temperature. After incubation, plates were washed four times with PBS/Tween20. Affinity purified horseradish peroxidase-conjugated goat anti-mouse IgG (catalog number: 170-6516, Bio-Rad, U.K.) diluted to 1:5000 was added to wells for 1 h at RT. After washing four times with PBS/Tween20, 50 μL of the substrate solution 3,3′,5,5′-tetramethylbenzidine was added per well and incubated in the dark at room temperature for 10 min. After sufficient color development, 30 μL of stop solution (2 N H_2_SO_4_) was added and the absorbance of each well was read with a POLARstar Omega microplate reader (BMG Labtech, Ortenberg, Germany) at 450 nm.

All animal experiments performed in this study were conducted in compliance with institutional guidelines.

### Statistical Information

All experimental results represent the mean ± standard deviation (SD) of at least three independent experiments unless specified. In real-time polymerase chain reaction (PCR) and western blot experiments, gene expression and protein amount were evaluated in relation to the housekeeping gene β-actin. Gene expression is represented as fold change compared to untreated cells, and results were evaluated using the one-sample Wilcoxon test. The western blots shown were representative data from at least two independent experiments. For ELISA experiments, means were compared by *t*-tests (two groups) or one-way ANOVA (three or more groups). Tukey multiple comparison test following one-way ANOVA was performed to obtain adjusted *P* values. For statistical comparisons of immunization results, the area under the ELISA titration curves was examined by Brown–Forsythe and Welch one-way ANOVA tests with an α of 0.05. This study includes no data deposited in external repository.

## Supporting Information Available

Molecular formula strings (CSV)
computational methods; docking calculations; synthesis procedures; all NMR spectra; cytotoxicity of compounds FP11 and FP18; log *P* and log *S* calculations (PDF)

## Abbreviations Used

AP-1: activator protein 1
CD14: cluster of differentiation 14
CSA: camphorsulfonic acid
ELISA: enzyme linked immunosorbent assay
ERK: extracellular regulated kinase
FA: fatty acid
IFNs: interferons
IFNAR: interferon-α/β receptor
IgG: immunoglobulin G
IL-: interleukin-
IRF3: interferon regulatory factor 3
ISGs: interferon stimulated genes
LPS: lipopolysaccharide
R-LPS: rough lipopolysaccharide
S-LPS: smooth lipopolysaccharide
MAPK: mitogen-activated protein kinase
MD: molecular dynamics
MD-2: myeloid differentiation factor 2
MPLA: monophosphoryl lipid A
MyD88: myeloid differentiation factor 88
NF-κB: nuclear factor kappa-light-chain enhancer of activated B cells
NLRP3: NLR family pyrin domain containing 3
NLRs: nucleotide-binding oligomerization domain (NOD)-like receptors
NT: untreated
OVA: ovalbumin
PAMPs: pathogen-associated molecular patterns
PRRs: pattern recognition receptors
RSAD2/Viperin: radical S-adenosyl methionine domain containing 2
SD: standard deviation
SEAP: secreted embryonic alkaline phosphatase
SPR: surface plasmon resonance
STAT1: signal transducer and activator of transcription
TBAF: tributylammonium fluoride
TFA: trifluoroacetic acid
TLRs: toll-like receptors
TNFα: tumor necrosis factor α
TDM: THP-1 derived macrophages
TRIF: TIR domain-containing adaptor inducing IFN-β
