# Supplementary material for: Synthetic Glycolipids as Molecular Vaccine Adjuvants: Mechanism of Action in Human Cells and In Vivo Activity
Source: J Med Chem. 2021 Aug 12;64(16):12261–72. doi: 10.1021/acs.jmedchem.1c00896 (PMC8404200; doi:10.1021/acs.jmedchem.1c00896)
Supplement: Supplementary file 2 — jm1c00896_si_002.pdf [file jm1c00896_si_002.pdf]

Supporting Information

Synthetic glycolipids as molecular vaccine adjuvants: mechanism of action in human cells and in vivo activity

Fabio A. Facchini[1§], Alberto Minotti[1§], Andrea Luraghi[1], Alessio Romerio[1], Nicole Gotri[1], Alejandra Matamoros-Recio[2], Andrea Iannucci[3,4], Charys Palmer[5], Guanbo Wang[6], Rebecca Ingram[6], Sonsoles Martin-Santamaria[2], Grisha Pirianov[5], Marco De Andrea[4,7], Miguel A. Valvano[6], Francesco Peri[1*]

[§]first co-authors

1 Department of Biotechnology and Biosciences, University of Milano-Bicocca, Piazza della Scienza, 2; 20126, Milano, Italy

2 Department of Structural and Chemical Biology, Centro de Investigaciones Biologicas Margarita Salas, C/ Ramiro de Maeztu, 9. 28040-Madrid, Spain.

3 Department of Translational Medicine, University of Eastern Piedmont, 28100 Novara, Italy

4 CAAD - Center for Translational Research on Autoimmune and Allergic Disease, University of Eastern Piedmont, 28100 Novara, Italy

5 Department of Biomedical and Forensic Sciences, Anglia Ruskin University, East Road, Cambridge CB1 1PT, UK

6 The Wellcome-Wolfson Institute for Experimental Medicine, Queen's University of Belfast; 97 Lisburn Rd. Belfast, UK BT9 7BL

7 Department of Public Health and Pediatric Sciences, University of Turin, Medical School, 10126 Turin, Italy

[*]**Corresponding author.** Email: francesco.peri@unimib.it.

Contents

Molecular Formula Strings……3

Computational Methods …… 4

Ligands 3D structure: construction, optimization and parameters derivation. …… 4

Macromolecule preparation…… 4

Docking calculations. …… 4

MD simulations. …… 4

Docking calculations of the binding of FP11, FP18, and FP111 to TLR4/MD-2 …… 5

MD simulations of $(TLR4/MD\text{-}2)_2$ complex with FP11 and FP18 …… 7

Chemical synthesis procedures …… 12

Compound 8 …… 12

Compound 9 …… 12

Compound 10 …… 13

Compound 11 …… 13

Compound 12 …… 13

Compound 13 …… 14

Compound 14 …… 14

Compound 15 …… 15

FP11 …… 15

FP18 …… 16

Compound 16 …… 16

Compound 17 …… 16

FP111 …… 17

NMR spectra …… 18

In vitro evaluation of the cytotoxicity of compounds FP11 and FP18 …… 33

Log P and log S calculations …… 34

References …… 35

Name	SMILES	Kd (µM)	TNF-α induction (pg/mL at 25 µM)	IL-1β induction (pg/mL at 25 µM)	IL-6 induction (pg/mL at 25 µM)	Antibody response to OVA immunization (AUC)
FP11	O=C(CCCCCCCCCCCCC)O[C@H]1[C@H](OC(CCCCCCCCCCCCC)=O)[C@@H](CO)O[C@H](OP(O[Na])(O[Na])=O)[C@@H]1NC(CCCCCCCCCCCCC)=O	0.18	50	100	80	7
FP111	O=C(CCCCCCCCCCCCC)O[C@H]1[C@H](OC(CCCCCCCCCCCCC)=O)[C@@H](COP(O[Na])(O[Na])=O)O[C@H](OP(O[Na])(O[Na])=O)[C@@H]1NC(CCCCCCCCCCCCC)=O	N/D	N/D	N/D	N/D	N/D
FP18	O=C(CCCCCCCCCCC)O[C@H]1[C@H](OC(CCCCCCCCCCC)=O)[C@@H](CO)O[C@H](OP(O[Na])(O[Na])=O)[C@@H]1NC(CCCCCCCCCCC)=O	0.57	300	450	0	9.5
1	N[C@@H]1[C@@H](O)[C@H](O)[C@@H](CO)O[C@H]1O					
2	O[C@H]1[C@H](N=[N+]=[N-])[C@@H](O)[C@H](O)[C@@H](CO)O1					
3	O[C@H]1[C@H](N=[N+]=[N-])[C@@H](O)[C@H]2[C@@H](CO[C@@H](C3=CC=C(OC)C=C3)O2)O1					
4	O[C@H]1[C@H]2[C@@H](CO[C@@H](C3=CC=C(OC)C=C3)O2)O[C@@H](O[Si](C)(C)C(C)(C)C)[C@@H]1N=[N+]=[N-]					
5	N[C@@H]1[C@@H](O)[C@H]2[C@@H](CO[C@@H](C3=CC=C(OC)C=C3)O2)O[C@H]1O[Si](C)(C)C(C)(C)C					
6	COC(C=C1)=CC=C1[C@H]2O[C@H]3[C@H](OC(CCCCCCCCCCCCC)=O)[C@@H](NC(CCCCCCCCCCCCC)=O)[C@H](O[Si](C)(C)C(C)(C)C)O[C@@H]3CO2					
7	COC(C=C1)=CC=C1[C@H]2O[C@H]3[C@H](OC(CCCCCCCCCCC)=O)[C@@H](NC(CCCCCCCCCCC)=O)[C@H](O[Si](C)(C)C(C)(C)C)O[C@@H]3CO2					
8	O[C@H]1[C@H](OC(CCCCCCCCCCCCC)=O)[C@@H](NC(CCCCCCCCCCCCC)=O)[C@H](O[Si](C)(C)C(C)(C)C)O[C@@H]1COCC2=CC=C(OC)C=C2					
9	O[C@H]1[C@H](OC(CCCCCCCCCCC)=O)[C@@H](NC(CCCCCCCCCCC)=O)[C@H](O[Si](C)(C)C(C)(C)C)O[C@@H]1COCC2=CC=C(OC)C=C2					
10	O=C(CCCCCCCCCCCCC)N[C@@H]1[C@@H](OC(CCCCCCCCCCCCC)=O)[C@H](OC(CCCCCCCCCCCCC)=O)[C@@H](COCC2=CC=C(OC)C=C2)O[C@H]1O[Si](C)(C)C(C)(C)C					
11	O=C(CCCCCCCCCCC)N[C@@H]1[C@@H](OC(CCCCCCCCCCC)=O)[C@H](OC(CCCCCCCCCCC)=O)[C@@H](COCC2=CC=C(OC)C=C2)O[C@H]1O[Si](C)(C)C(C)(C)C					
12	O[C@H]1[C@H](NC(CCCCCCCCCCCCC)=O)[C@@H](OC(CCCCCCCCCCCCC)=O)[C@H](OC(CCCCCCCCCCCCC)=O)[C@@H](COCC2=CC=C(OC)C=C2)O1					
13	O[C@H]1[C@H](NC(CCCCCCCCCCC)=O)[C@@H](OC(CCCCCCCCCCC)=O)[C@H](OC(CCCCCCCCCCC)=O)[C@@H](COCC2=CC=C(OC)C=C2)O1					
14	O=C(CCCCCCCCCCCCC)N[C@@H]1[C@@H](OC(CCCCCCCCCCCCC)=O)[C@H](OC(CCCCCCCCCCCCC)=O)[C@@H](COCC2=CC=C(OC)C=C2)O[C@@H]1OP(OCC3=CC=CC=C3)(OCC4=CC=CC=C4)=O					
15	O=C(CCCCCCCCCCC)N[C@@H]1[C@@H](OC(CCCCCCCCCCC)=O)[C@H](OC(CCCCCCCCCCC)=O)[C@@H](COCC2=CC=C(OC)C=C2)O[C@@H]1OP(OCC3=CC=CC=C3)(OCC4=CC=CC=C4)=O					
16	OC[C@H]1O[C@H](O)[C@H](NC(CCCCCCCCCCCCC)=O)[C@@H](OC(CCCCCCCCCCCCC)=O)[C@@H]1OC(CCCCCCCCCCCCC)=O					
17	O=C(CCCCCCCCCCCCC)N[C@@H]1[C@@H](OC(CCCCCCCCCCCCC)=O)[C@H](OC(CCCCCCCCCCCCC)=O)[C@@H](COP(OCC2=CC=CC=C2)(OCC3=CC=CC=C3)=O)O[C@@H]1OP(OCC4=CC=CC=C4)(OCC5=CC=CC=C5)=O					

Computational Methods

Ligands 3D structure: construction, optimization and parameters derivation. The 3D structures of FP11, FP18 and FP111 were built with PyMOL molecular graphics and modeling package (www.pymol.org), using as a template the 6YA monosaccharide (GLYCAM database, www.glycam.org). The structures of the ligands were first refined at the AM1 level of theory, and then optimized at the Hartree-Fock level (HF/6-311G**) with Gaussian09.[1] The parameters needed for MD simulations were obtained using the standard Antechamber procedure in Amber14.[2] The partial charges were derived from the HF calculations, and formatted for AmberTools15 and Amber14 with Antechamber, assigning the general AMBER force field (GAFF) atom types, and GLYCAM force field atom types for the saccharide atoms.

Macromolecule preparation. 3D coordinates from the X-ray structure of the human $(TLR4/MD\text{-}2/\textit{E. coli}\ LPS)_2$ ectodomain (PDB ID 3FXI) were retrieved from the Protein Data Bank (www.rcsb.org), and the chains A (TLR4) and C (MD-2) were extracted and considered as TLR4/MD-2 heterodimer in agonist conformation. Ligands and ions were removed. Hydrogen atoms were added to the X-ray structure using the preprocessing tool of the Protein Preparation Wizard of the Maestro package (www.schrodinger.com). The protein structure went through a restrained minimization under the OPLS3 force field with a convergence parameter to RMSD for heavy atoms kept default at 0.3 Å.

Docking calculations. AutoDockTools 1.5.6 program[3] was used to assign the Gasteiger-Marsili empirical atomic partial charges to the atoms of both the ligands and the receptor. Nonpolar hydrogens were merged for the ligands. The structure of the receptor and the ligands were set rigid and flexible, respectively. Preliminary docked poses were obtained with AutoDock Vina 1.1.2.[4] The box spacing was set to the default value of 1 Å; the size of the box was set to 33.00, 40.50, and 35.25 Å in the x, y, z-axes respectively, with the box center located equidistant to the mass center of residues Arg90 (MD-2), Lys122 (MD-2) and Arg264 (TLR4). The best-predicted docked poses from AutoDock Vina were redocked with AutoDock 4.2[3] using the Lamarckian evolutionary algorithm; all parameters were kept default except that the number of genetic algorithm runs was set to 100 to enhance the sampling. Docking box spacing was set to 0.375 Å and box size was set to the same dimensions as for AutoDock Vina.

MD simulations. Selected docked complexes were submitted to all-atom MD simulations for 50 ns in the Amber14 suite using the force field ff14SB to describe the protein system. The simulation box was designed such that the edges were distant at least 10 Å of any atom. The system was solvated with the TIP3P water molecules model; Na^+ ions were added to counterbalance the charges of the protein-ligand systems. All the simulations were performed with the same equilibration and production protocol. First, the system was

submitted to 1000 steps of the steepest descent algorithm followed by 7000 steps of the conjugate gradient algorithm. A 100 kcal·mol^{-1}·Å^{-2} harmonic potential constraint was applied to both the proteins and the ligand. In the subsequent steps, the harmonic potential was progressively lowered (respectively to 10, 5, and 2.5 kcal·mol^{-1}·Å^{-2}) for 600 steps of the conjugate gradient algorithm each time, and then the whole system was minimized uniformly. Next, the system was heated from 0 to 100 K using the Langevin thermostat in the canonical ensemble (NVT) while applying a 20 kcal·mol^{-1}·Å^{-2} harmonic potential restraint on the protein and the ligand. Finally, the system was heated from 100 to 300 K in the isothermal-isobaric ensemble (NPT) under the same restraint condition as in the previous step and followed by simulation for 100 ps with no harmonic restraint. At this point, the system was ready for the production run, which was performed using the Langevin thermostat under the NPT ensemble, at a 2 fs time step. All production runs were performed for 150 ns. The analysis was performed using the cpptraj module of AmberTools.[5] Atomic coordinates of the TLR4 complexes (TLR4-FP11-01.pdb, TLR4-FP11-02.pdb, TLR4-FP18.pdb, TLR4-FP111-A.pdb and TLR4-FP111-B.pdb) after simulation are available.

Docking calculations of the binding of FP11, FP18, and FP111 to TLR4/MD-2

A deeper analysis of the docked binding poses revealed, in the case of FP18, a tendency for all poses to bury the three lipid acyl tails deep inside the hydrophobic cavity of MD-2, occupying the full pocket. In the case of FP11, different docked poses were obtained, most of them with all the FA chains allocated inside the MD-2 pocket, and other poses with one acyl chain located into the hydrophobic channel of MD-2, delimited by residues Arg90 and Phe126, and the other FA chains inside the MD-2 cavity (Fig. 1B, top). This second binding pose is similar to that observed for *E. coli* LPS in human and murine $(TLR4/MD\text{-}2)_2$ heterotetramers (PDB ID 3FXI[6] and 3VQ2[7]), where one FA chain is found occupying this channel. The presence of longer acyl chains in FP11 (C14 vs C12 in FP18) may favor the placement of one of them protruding into the MD-2 channel, whereas this phenomenon was not observed for FP18. Regarding FP111, in all AutoDock4 predicted poses, at least one FA tail was left outside the MD-2 pocket, without interacting either in the MD-2 Phe126 channel, and remaining exposed to the outer (Fig. 1B, bottom, left). These binding modes justify the unfavorable predicted binding energies.

The best docked clusters from each compound were visually inspected, with special attention to the ligand/receptor interactions, to establish a relationship between the chemical structure of the ligands and their effect on functional activity of TLR4 receptor. For FP11 and FP18, common features were observed. The polar head groups were placed at the entrance of MD-2 cavity, interacting with the polar residues present in that region, whereas the FA chains established contacts with many hydrophobic residues from the MD-2 pocket, specifically, Val24, Ile32, Ile46, Val48, Ile52, Leu54, Leu61, Ile63, Leu74, Phe76, Leu78, Ile80, Val82, Leu87, Ile94, Tyr102, Phe104, Ile117, Phe119, Phe121, Ile124, Phe126, Ser127, Tyr131, Val135, Phe147,

Leu149, Phe151, and Ile153. The ligands phosphate groups were often placed at the rim of MD-2 where they are exposed to the solvent, in agreement with the reported X-ray crystallographic complexes of TLR4/MD-2 with glycolipids (for example, complex with Eritoran, PDB ID 2Z65,[8] or with lipid IVa, PDB ID 2E59[9]). Two different orientations have been reported for TLR4 binders, rotated 180° between them[10]: type A (antagonist-like binding mode), similar to that found for lipid IVa in PDB ID 2E59;[7] and type B (agonist-like binding mode), similar to that found for *E. coli* lipid A in PDB ID 3FXI.[6] These two different binding types lead to opposed biological activities.[11] Remarkably, the obtained docked poses for FP11 and FP18 showed a type B like binding mode, in agreement with their reported *in vitro* agonist activity.

Most of the FP11 and FP18 poses presented the hydroxyl group of the saccharide moiety interacting with the Glu92 side chain from MD-2, and the amide CO group was often close to the Arg90 of MD-2, establishing hydrogen bonds and electrostatic interactions, while the ester oxygen of the 4-acyl chain interacted with the hydroxyl group of the MD-2 Ser120. The main difference observed between FP11 and FP18 ligands was related to the interactions established by the phosphate group; in FP11 poses, the phosphate interacted with the backbone of MD-2 Lys122, whereas in FP18 interacted with MD-2 Arg90 (Fig. 1B, top, right). The relevance of the interactions between the studied ligands and the above-mentioned MD-2 residues, which are key residues in the recognition of *E. coli* LPS by the TLR4/MD-2 complex according to the literature data must be highlighted. [6,12]

As for FP111, although AutoDock Vina was able to produce plausible docked poses inside the TLR4/MD-2 system, AutoDock did not lead to poses with favorable predicted binding energies. It is well-known the better performance correlation between predicted binding free energy and experimental value for AutoDock *versus* AutoDock Vina, as well as better precision and success rates.[13] Remarkably, FP111 poses showed the two above mentioned types of ligand poses, A and B, along the results generated by means of both docking programs (Fig. 1B, bottom, right). In any case, we also analyzed the docking results for FP111 from AutoDock. FP111 poses were predicted to be anchored through one phosphate group to MD-2, and the second phosphate to positively charged residues present in TLR4; in the type A poses, the 6-phosphate group interacted with TLR4 residues, equivalent to the interaction of the 1-phosphate in the type B poses (Fig. 1B, bottom). The 6-phosphate should mimic the interaction of the hydroxyl group present in FP11 and FP18 but, given a bigger size and different electronic distribution, this interaction is not possible and tries to reach positive residues at TLR4. As a consequence of these contacts between FP111 and TLR4, the generated poses remained not as deep inside the MD-2 pocket as in the FP11 and FP18 cases, and more exposed to the solvent. These observations could explain the unfavorable predicted binding energy. We selected two of the best binding poses for further study, one type A (antagonist-like binding pose, Fig. 1B, bottom, right), and one type B (agonist-like binding pose, Fig. 1B, bottom, right). In the type A docked pose, rotated 180° with respect to FP11 and FP18 predicted poses, the 1-phosphate interacted with TLR4 Arg264, establishing two

hydrogen bonds, and with MD-2 Tyr102 residue, whereas the 1-phosphate interacted with MD-2 residues Ser118 and Ser120. The 4-acyl chain was found outside MD-2, interacting with unexpected residues, such as MD-2 Gly56, Ser57 and Lys58. These residues have not been reported before in the interaction between TLR4 modulators and the receptor complex. The other two FP111 acyl chains, buried inside MD-2, established contacts with Leu61, Phe76, Leu78, Ile94, Phe121, Lys122, Ile124, Phe126, Cys133, Val135 and Phe151. In the type B orientation, the 1-phosphate interacted with MD-2 Arg90 and Glu92 residues, establishing a hydrogen bond with the latter one, similar to the interaction previously described for the hydroxyl group of FP11 and FP18, and the 6-phosphate interacted with the Lys362 of TLR4. The disposition adopted by FP111 due to this last contact, forces to place the 4-acyl chain outside the MD-2 cavity, establishing a hydrogen bond between the ester CO group and the TLR4 Arg264 side chain. Additionally, other electrostatic and hydrophobic interactions were observed between this acyl tail and TLR4 residues, namely Asp101, Tyr292, Leu293, Tyr296, Ser317, Val318, Thr319, Asn339, Cys340 and Lys341, and between the remaining FA chains, located inside MD-2 protein, and MD-2 residues, such as Val24, Ile32, Ile46, Val48, Leu61, Ile63, Phe76, Leu78, Ile94, Tyr102, Phe104, Phe117, Phe119, Val135, Phe147 and Phe151. It is well known that the determining factors of the immunostimulatory activity of TLR4 modulators, are the number and the distribution of acyl chains and the phosphorylation pattern. As example, the orientation of the lipid IVa is rotated by 180° in the di-saccharide plan thus lipid IVa presents two different molecular patterns of interaction for human and mouse TLR4 receptors. These different binding modes of lipid IVa determine how the phosphate groups interact with the TLR4/MD-2 complex and are crucial for the observed different behavior among both species.[11] Interestingly, the distances between the two phosphates in all the FP111 docked solutions ranged from 5.4 to 8.2 Å, values lower to the distance between the two phosphates of the agonist *E. coli* lipid A at C1 and C4' positions (distance of 12.4 Å in PDB ID 3FXI[6]). Specifically, the two phosphate groups were placed at a distance of 6.5 Å in the selected FP111 type A pose, and of 8 Å in the type B. Overall, we can conclude there is a different binding pattern for FP111 in comparison to FP11 and FP118, not including extensively reported and well-known TLR4/MD-2/ligand interactions,[10] as well as unfavorable predicted binding energies. This anomalous behavior can be explained by the presence of two phosphate groups in 1, 6-positions simultaneously, that cannot allow a proper docking into the MD-2 rim and need to anchor to TLR4, unlike the mono (1-or 6-) phosphate pattern, accounting for the lack of activity observed for this compound.

MD simulations of $(TLR4/MD\text{-}2)_2$ complex with FP11 and FP18

Stability of the best FP11 and FP18 predicted binding modes was confirmed by molecular dynamics (MD) simulations. Starting from the docked TLR4/MD-2/ligand complexes, we constructed three $(TLR4/MD\text{-}2/ligand)_2$ heterodimer models (Fig. S1a) which were submitted to 50 ns MD simulations: the best pose from the FP18 docking calculations (Fig. S1b), and two from the FP11 results; one pose with all the ligand acyl

chains inside the MD-2 pocket (FP11-01, Fig. S1b), and another one with one FA chain protruding towards the MD-2 channel, and the other chains folded into the MD-2 cavity (FP11-02, Fig. S1c). After 50 ns simulation, the complexes remained stable, as shown by the RMSD analysis (Fig. S2).

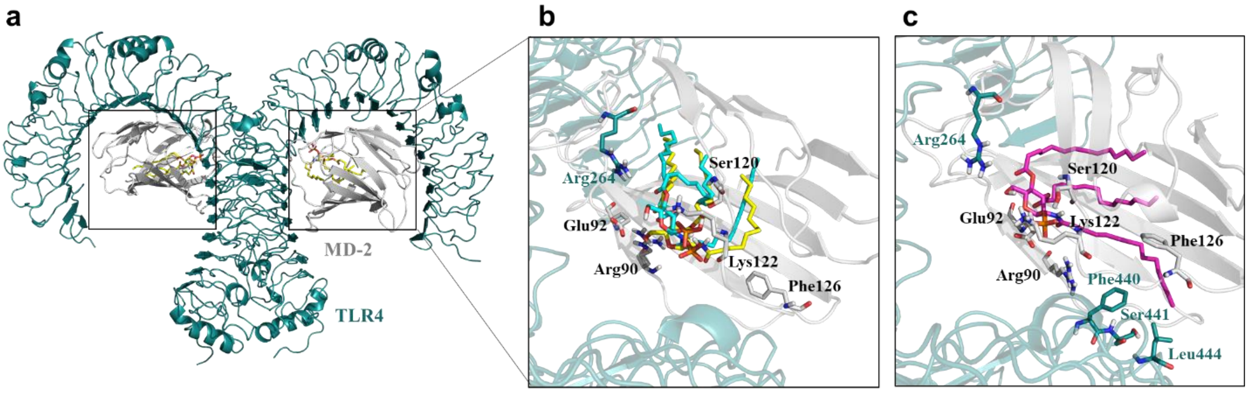

Fig. S1: MD simulations of ligands FP11 and FP18 selected binding modes.

a General view of the simulated (TLR4/MD-2/FP18)$_2$ complex, as example. **b, c** Details of the averaged interactions between FP11 (blue and magenta sticks) and FP18 (yellow sticks) and TLR4/MD-2, over simulation time. During simulations, in the FP11 binding mode with the 2-acyl chain protruding within the MD-2 channel, this FA chain remained outside from the MD-2 pocket, pointing toward the partner TLR4, only in one of the (TLR4/MD-2/FP11)$_2$ subunits, interacting with residues Phe440, Ser441 and Leu444 from the partner TLR4 (**c**, FP11 as magenta sticks). Residue MD-2 Phe126 remained in agonist conformation throughout simulation time (t = 50 ns). TLR4 and MD-2 are respectively depicted in turquoise blue and grey cartoons. Residues that interacted with the ligands are in sticks with their corresponding individual labels.

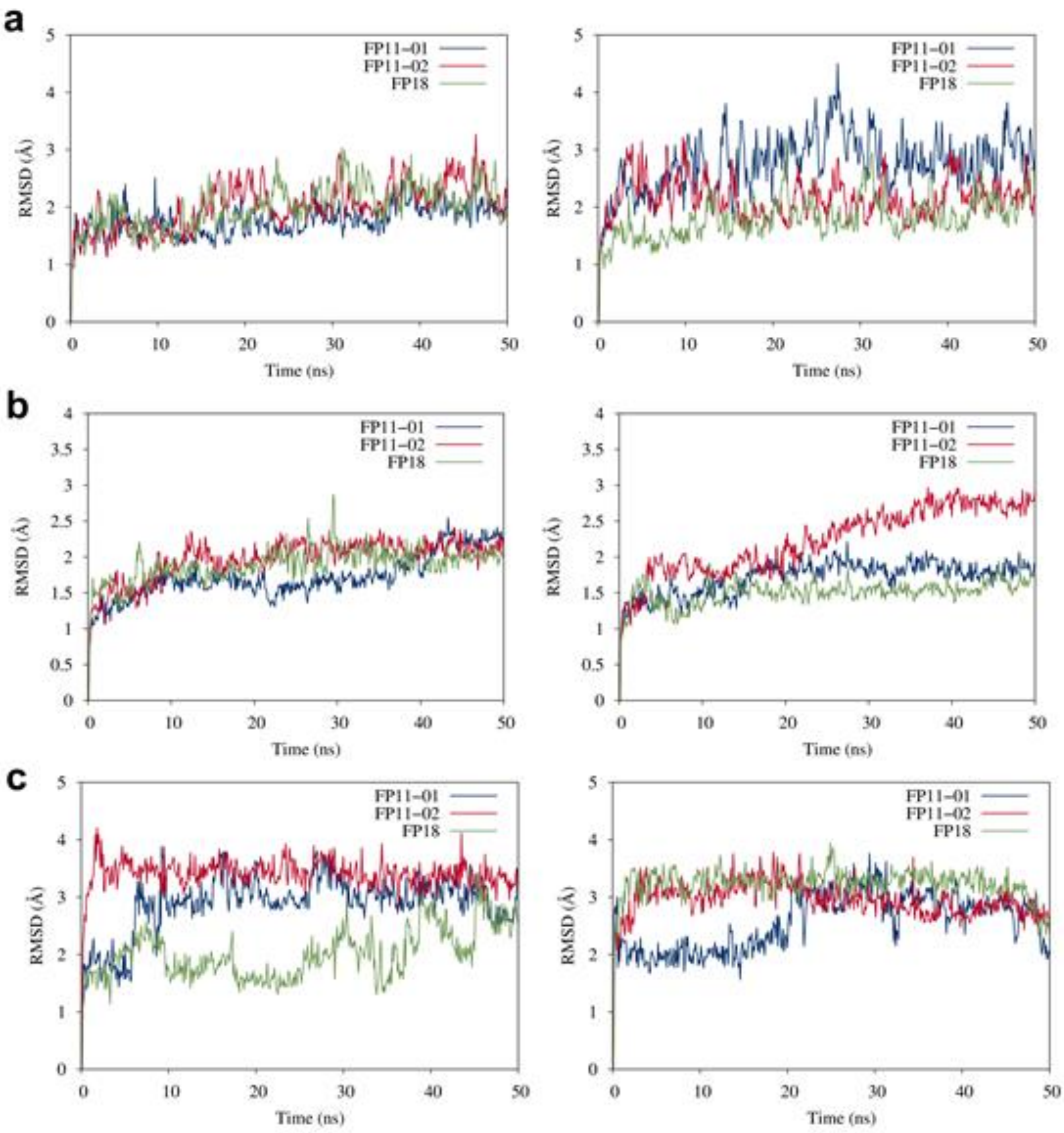

Fig. S2: Molecular dynamics simulations of the (TLR4/MD-2)2 system in complex with ligands FP11 and FP18. a RMSD of the TLR4 chain A (on the left) and B (on the right) backbone over time. **b** RMSD of the MD-2 chain A (on the left) and B (on the right) backbone, over time. **c** RMSD for the heavy atoms of the ligands docked in TLR4/MD-2 chain A (on the left) and B (on the right), over time.

The relative orientation between the ligands and MD-2 was evaluated. We arbitrarily defined two vectors, one from the amide α-carbon atom to the ester α-carbon atom of the ligand, and another one from the α-carbon of residues Pro78 to Thr105 of MD-2 (Fig. S3a). The angle between these two vectors was plotted both over time, and it was observed that none of the ligands undergoes orientation flip during the 50 ns simulations, all remaining in the agonist type B orientation obtained from the docking calculations (Fig. S3b). Furthermore, the motion of the TLR4 molecular switch, MD-2 Phe126 chain was also evaluated (Fig. S4). FP11 and FP18 ligands were able to retain the agonist conformation for MD-2 Phe126 along simulation time.

Therefore, we suggest these complexes as plausible binding modes for FP11 and FP18 accounting for their agonist activity in the TLR4/MD-2 system.

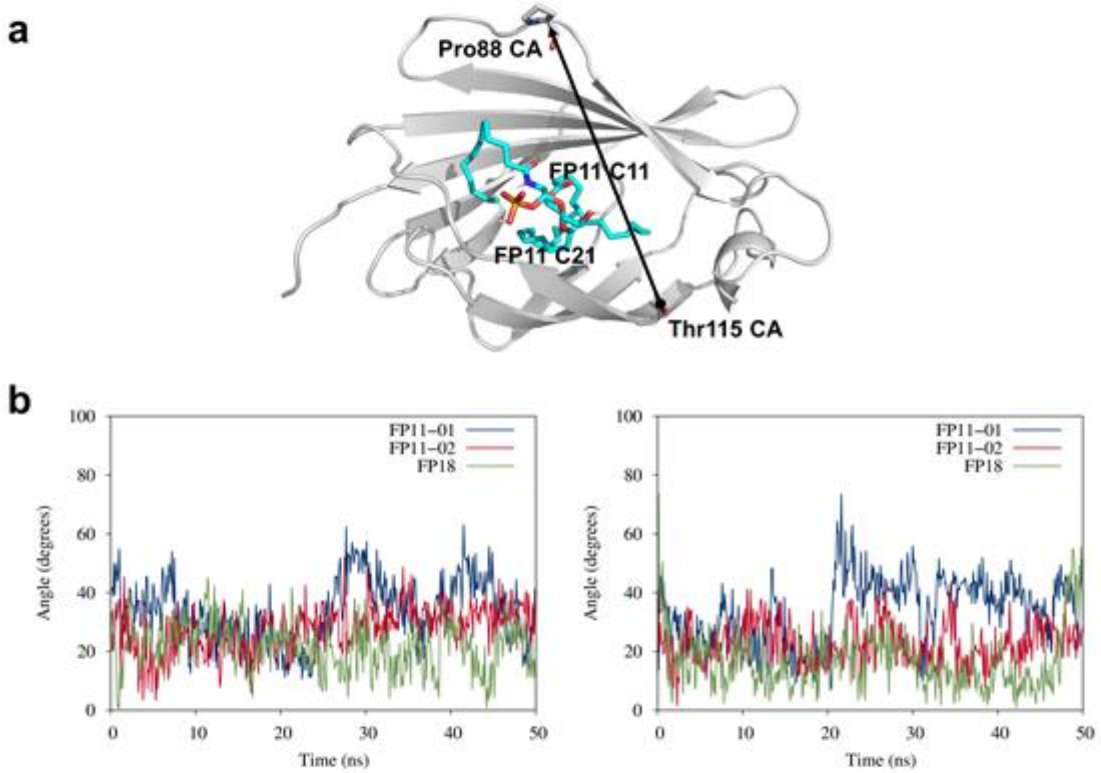

Fig. S3: Ligands orientation within the MD-2 pocket. a Angle plotted over simulation time between two arbitrarily selected vectors starting from the α-carbon (CA) of Pro88 to the α-carbon of Thr115, and the vector from amide α-carbon atom (C11) and the ester αcarbon atom (C21) of FP11, used to follow the orientation of the ligands along the MD simulations. Angle for MD-2 chain A, on the left and chain B, on the right. FP11 is used as an example; the same applies for the other ligands. **b** Representation of the two selected vectors, within MD-2. Angle between 0 and 90 degrees is characteristic of the type B binding (agonist-like) as observed in the PDB ID 3FXI (TLR4/MD-2/*E. coli* LPS)$_2$ complex; angle between 90 and 180 degrees is characteristic of the type A binding mode (antagonist-like) as observed in the PDB ID 2E59 (MD-2/lipid-IVa) complex. MD-2 is represented as semi-transparent grey cartoon, and FP11 ligand as cyan sticks.

During the MD simulations, the similar interactions were observed for FP11 (FP11-01 and FP11-02) and FP18 compounds, in both TLR4/MD-2 units. The ligands suffered a slightly reorientation of the saccharide moiety, which allowed them to establish a new electrostatic contact with each corresponding TLR4 chain, concretely between the hydroxyl group of the ligands and the TLR4 Arg264 residue (Fig. S1b, c). Consequently, the interaction between the hydroxyl group of the compounds and MD-2 Glu92 was lost in all the cases, and this residue became to interact with both, the ester CO group or the oxygen of the ligands 2-acyl chain, depending on the case. Interestingly, in the MD simulation of the (TLR4/MD-2/FP18)$_2$ complex, the interaction between the phosphate group and MD-2 Arg90 was lost and two new contacts with MD-2

residues were established, a hydrogen bond with the backbone of Lys122, as observed in the FP11 docked poses, and a polar contact with Ser120. Regarding FP11 poses, a new polar contact was displayed between the ligand 1-phosphate group and MD-2 Ser120, as in FP18 simulation, additional to the initially present interaction with Lys122, predicted by docking programs, which was maintained along the simulations. The interaction between Arg90 and the amide CO group of the 2-acyl chain was maintained in all the ligands. Remarkably, when we constructed the full (TLR4/MD-2/ligand)$_2$ heterotetramer model of the FP11-02 pose for running the MD simulations, the FP11 lipid chain placed in the MD-2 channel, interacted with residues of the partner TLR4, concretely, Phe440 and Ser441. During the simulation of the (TLR4/MD-2/FP11-02)$_2$ complex, this chain remained outside from the MD-2 pocket, pointing toward the partner TLR4, only in one of the (TLR4/MD-2/FP11-02)$_2$ subunits. The interactions between this chain and the partner TLR4 residues Phe440 and Ser441, observed when we constructed the full (TLR4/MD-2/FP11-02)$_2$ complex, were maintained over time (Fig. S1c), and one more interaction was established between this FA chain and the counterpart TLR4 Leu444 residue. On the contrary, the same FP11-02 FA chain of the TLR4 counterpart, was folded into the MD-2 cavity during the simulation . The electrostatic and hydrophobic interactions with the rest of MD-2 residues, predicted by docking calculations, were maintained.

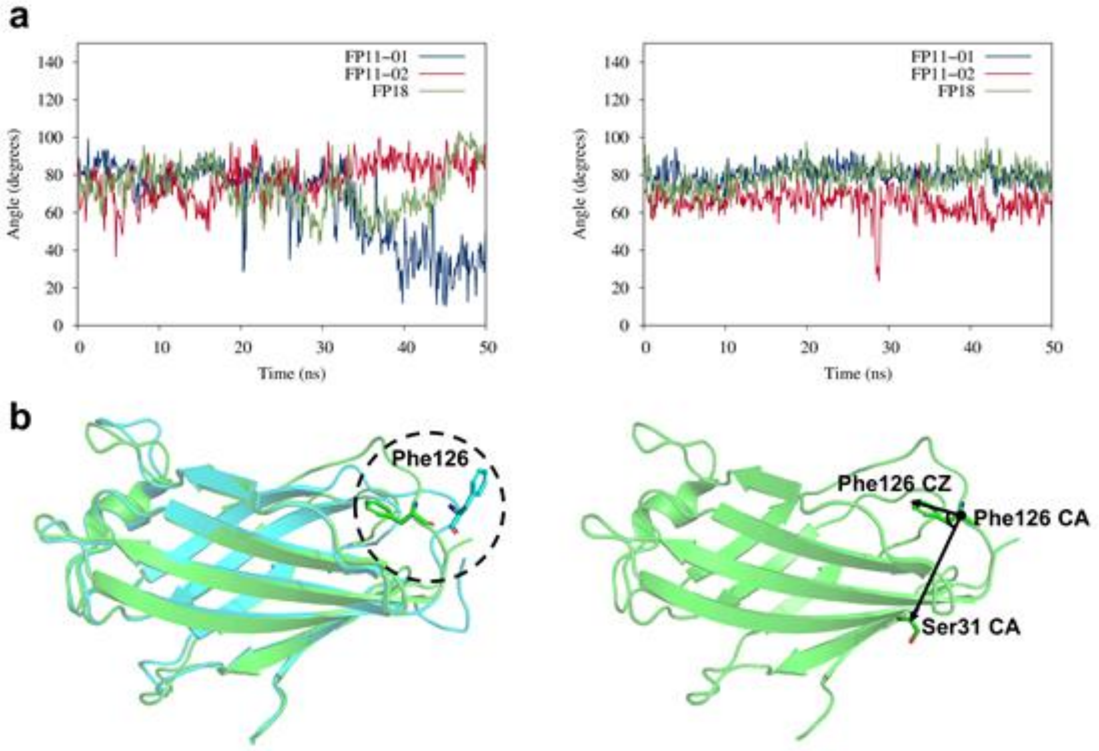

Fig. S4: Motion of the TLR4/MD-2 antagonist/agonist switch Phe126. a Angle plotted over simulation time between two arbitrarily selected vectors starting both from the α-carbon (CA) of residue MD-2 Phe126 to, respectively, the zeta-carbon (CZ) of the same residue and the α-carbon (CA) of residue Ser31. Angle for MD-2 chain A, on the left and chain B, on the right. **b** On the left: Superposition of the X-ray crystallographic

structures of the agonist (green) and the antagonist (cyan) conformations of MD-2 from, respectively, PDB ID 3FXI and 2E59. On the right: representation of the two selected vectors, within MD-2. Agonist MD-2 from PDB ID 3FXI and antagonist MD-2 from PDB ID 2E59 are represented in semi-transparent green and cyan cartoon, respectively. Bound ligands have been hidden for clarity (*E. coli* LPS in 3FXI; lipid IVA in 2E59). Conformational changes of the molecular switch Phe126 are marked. The angle plotted over time shows the stability of residue Phe126 during MD simulations associated with agonist activity of ligands. Angle between 0 and 100 degrees is characteristic of the agonist-like binding.

Chemical synthesis procedures

Compound 8

tert-butyldimethylsilyl-2-tetradecanamido-2-deoxy-3-O-tetradecanoyl-6-O-(4-methoxybenzyl)-β-D-glucopyranoside (**8**). Compound **6**[10] (320 mg, 0.38 mmol) was dissolved in dry THF (19 mL), 4Å molecular sieves (1.15 g) and $NaCNBH_3$ (157 mg, 18.38 mmol) were added. The reaction was stirred at r.t. for 1h, then TFA (581 mL, 7,6mmol) was added dropwise at 0°C. The resulting solution was left stirring for additional 45 min at r.t.. After neutralization with saturated $NaHCO_3$ solution, the mixture was extracted with DCM. Combined organic phases were with brine, dried over anhydrous Na_2SO_4, filtered and evaporated *in vacuo*.

Crude was purified with flash chromatography (petroleum ether-EtOAc 8:2) to give compound **8** as a red oil (273 mg, 85%).

^{1}H NMR (400 MHz, $CDCl_3$, 25 °C, TMS) δ 7.23 (d, $^3J_{H,H}$=8.6 Hz, 2H; 2x H-*ortho* PMB), 6.85 (t, $^3J_{H,H}$=8.7 Hz, 2H: 2x H-*meta* PMB), 5.81 (d, $^3J_{H,H}$=9.3 Hz, 1H; NH), 5.04 (dd, $^3J_{H,H}$=10.7, 9.2 Hz, 1H; H-3), 4.67 (d, $^3J_{H,H}$=8.1 Hz, 1H, H-1), 4.54-4.42 (m, 2H; CH_2-Ph), 3.94 (m, 1H; H-2), 3.78 (s, 4H; OMe, H-5), 3.70 (dd, $^3J_{H,H}$=10.8, 5.3 Hz, 1H, H-6a), 3.54 (m, 2H, H-4, H-6b), 2.38-2.24 (t, $^3J_{H,H}$=7.8 Hz, 2H, CH_2α-chain1), 2.15-1.98 (m, 2H, CH_2α-chain2),1.64-1.44 (m, 4H CH_2β-chains1,2), 1.19 (m, 40H, 20x CH_2), 0.96-0.70 (m, 15H, 2xCH_3 chains1,2 ,tBu-Si), 0.03 (s, 3H; CH_3-Si), 0.00 (s, 3H; CH_3-Si).

13C NMR (101 MHz, $CDCl_3$, 25 °C, TMS) δ 174.88, 172.79, 159.29, 129.66, 129.30, 113.80, 96.53, 75.26, 73.71, 73.42, 71.25, 70.57, 55.46, 55.23, 36.98, 34.28, 31.93, 29.72, 29.68, 29.55, 29.47, 29.38, 29.36, 29.14, 25.57, 24.97, 22.69, 17.88, 14.13.

HRMS (ESI): m/z [M+Na$^+$] calculated for $C_{48}H_{87}NNaO_8Si^+$: 856.2972. Found: 856.2977.

Compound 9

tert-butyldimethylsilyl-2-dodecanamido-2-deoxy-3-O-dodecanoyl-6-O-(4-methoxybenzyl)-β-D-glucopyranoside (**9**). Compound **9** was synthesized starting from **7**[10] accordingly to the procedure presented for compound **8** in 83% yield.

^{1}H NMR (400 MHz, $CDCl_3$, 25 °C, TMS) δ 7.19 (d, $^3J_{H,H}$=8.3 Hz, 2H; 2x H-*ortho* PMB), 6.82 (t, $^3J_{H,H}$=8.5 Hz, 2H: 2x H-*meta* PMB), 6.29 (d, $^3J_{H,H}$=9.6 Hz, 1H; NH), 5.09 (t, $^3J_{H,H}$=9.9 Hz, 1H; H-3), 4.67 (d, $^3J_{H,H}$=8.0 Hz, 1H, H-1), 4.55-4.37 (m, 2H; CH_2-Ph), 3.94 (q, , $^3J_{H,H}$=9.6 Hz, 1H; H-2), 3.75 (s, 4H; OMe, H-5), 3.66 (dd, $^3J_{H,H}$=9.7, 7.2 Hz, 1H, H-6a), 3.56 (m, 2H, H-4, H-6b), 2.36-2.20 (m, 2H, CH_2α-chain1), 2.16-2.01 (m, 2H, CH_2α-chain2), 1.64-1.44 (m, 4H CH_2β-chains1,2), 1.22 (m, 32H, 16x CH_2), 0.92-0.77 (m, 15H, 2xCH_3 chains1,2 ,tBu-Si), 0.05 (s, 3H; CH_3-Si), 0.02 (s, 3H; CH_3-Si).

^{13}C NMR (101 MHz, $CDCl_3$, 25 °C, TMS) δ 175.13, 173.04, 159.53, 129.91, 129.55, 114.05, 96.78, 75.51, 73.96, 73.67, 71.49, 70.82, 55.71, 55.48, 37.23, 34.53, 32.18, 29.97, 29.93, 29.80, 29.72, 29.63, 29.60, 29.39, 25.82, 25.22, 22.94, 18.13, 14.38.

HRMS (ESI): m/z [M+Na$^+$] calculated for $C_{44}H_{79}NNaO_8Si^+$: 800.5467. Found: 800.5471.

Compound 10

***10** tert-butyldimethylsilyl-2-tetradecanamido-2-deoxy-3,4-di-O-tetradecanoyl-6-O-(4-methoxybenzyl)-β-D-glucopyranoside* (**10**). To a 0°C cooled solution of compound **8** (263 mg, 0.32 mmol), DMAP (39 mg, 0.32 mmol), and TEA (87µL ,0.63 mmol) in DCM (5 mL), myrystoyl chloride (170 µL ,0.63 mmol)was added dropwise. The solution was stirred at r.t. for 1h. Solvents were evaporated *in vacuo* and crude product purified with flash chromatography (petroleum ether–EtOAc 9:1) to give compound **10** as a white solid (323 mg, 98%).

^1H NMR (400 MHz, $CDCl_3$, 25 °C, TMS) δ 7.23 (d, $^3J_{H,H}$=8.4 Hz, 2H, 2x H-*ortho* PMB), 6.85 (d, $^3J_{H,H}$=8.4 Hz, 2H, 2x H-*meta* PMB), 5.30 (d, 1H, NH), 5.15 (t, $^3J_{H,H}$=10.2 Hz, 1H, H-3), 5.03 (t, $^3J_{H,H}$=9.6 Hz, 1H, H-4), 4.77 (d, $^3J_{H,H}$=8.1 Hz, 1H, H-1), 4.45 (s, 2H, CH_2-Ph), 3.96-3.85 (m, 1H, H-2), 3.79 (s, 3H, OCH_3), 3.66-3.58 (m, 1H, H-5), 3.51 (m, 2H, H-6a, H-6b), 2.23 (t, $^3J_{H,H}$=7.8 Hz, 2H, CH_2α-chain1), 2.14 (t, $^3J_{H,H}$=7.6 Hz, 2H, CH_2α-chain2), 2.10-2.02 (m, 2H, CH_2α-chain3),1.63-1.43 (m, 6H, CH_2β-chains1,2,3), 1.24 (m, 60H, 30xCH_2), 0.94-0.79 (m, 15H, 2xCH_3-chains1,2 ,tBu-Si), 0.12 (s, 3H; CH_3-Si), 0.08 (s, 3H; CH_3-Si).

^{13}C NMR (101 MHz, $CDCl_3$, 25 °C, TMS) δ 173.89, 172.59, 172.26, 159.14, 129.98, 129.27, 113.67, 96.37, 73.50, 73.19, 72.31, 69.33, 69.27, 56.26, 55.23, 36.91, 34.18, 34.11, 32.78, 31.92, 29.67, 29.50, 29.36, 29.32, 29.14, 26.40, 25.58, 24.94, 24.79, 22.69, 17.88, 14.12, -4.02, -5.23.

HRMS (ESI): m/z [M+Na$^+$] calculated for $C_{62}H_{113}NNaO_9Si^+$: 1066.8077. Found: 1066.8081.

Compound 11

tert-butyldimethylsilyl-2-dodecanamido-2-deoxy-3,4-di-O-dodecanoyl-6-O-(4-methoxybenzyl)-β-D-glucopyranoside (**11**). Compound **11** was synthesized starting from **9** accordingly performing the acylation presented for compound **10** with lauroyl chloride. Compound was obtained in 97% yield.

^1H NMR (400 MHz, $CDCl_3$, 25 °C, TMS) δ 7.22 (d, $^3J_{H,H}$=8.6 Hz, 2H, 2x H-*ortho* PMB), 6.85 (d, $^3J_{H,H}$ =8.6 Hz, 2H, 2x H-*meta* PMB), 5.34 (d, J =9.3 Hz, 1H, NH), 5.15 (t, $^3J_{H,H}$=9.9 Hz, 1H, H-3), 5.02 (t, $^3J_{H,H}$=9.6 Hz, 1H, H-4), 4.76 (d, $^3J_{H,H}$=8.0 Hz, 1H, H-1), 4.44 (s, 2H, CH_2-Ph), 3.91 (dt, $^3J_{H,H}$ =10.8, 8.5 Hz, 1H, H-2), 3.79 (s, 3H, OCH_3), 3.64 (dt, $^3J_{H,H}$ =9.6, 4.5 Hz, 1H, H-5), 3.54-3.48 (m, 2H, H-6a, H-6b), 2.22 (t, $^3J_{H,H}$=7.6 Hz, 2H, CH_2α-chain1), 2.14 (t, $^3J_{H,H}$=7.6 Hz, 2H, CH_2α-chain2), 2.09-2.04 (m, 2H, CH_2α-chain3), 1.59-1.43 (m, 6H, CH_2β-chains1,2,3), 1.32-1.21 (m, 48H, 24xCH_2 chains), 0.91-0.83 (m, 15H, 2xCH_3-chains1,2 ,tBu-Si), 0.12 (s, 3H; CH_3-Si), 0.09 (s, 3H; CH_3-Si).

^{13}C NMR (101 MHz, $CDCl_3$, 25 °C, TMS) δ 173.98, 172.68, 172.34, 159.22, 130.06, 129.35, 113.75, 96.45, 73.58, 73.28, 72.39, 69.41, 69.35, 56.34, 55.31, 37.00, 34.26, 34.20, 32.87, 32.00, 29.76, 29.58, 29.45, 29.40, 29.22, 26.49, 25.67, 25.02, 24.88, 22.77, 17.97, 14.21, -3.94, -5.15.

HRMS (ESI): m/z [M+Na$^+$] calculated for $C_{56}H_{101}NNaO_9Si^+$: 982.7138. Found: 982.7143.

Compound 12

2-tetradecanamido-2-deoxy-3,4-di-O-tetradecanoyl-6-O-(4-methoxybenzyl)-β-D-glucopyranose (**12**). Compound **10** (323 mg, 0.31 mmol) was dissolved in dry THF (15 mL), and cooled to -15 °C. A solution of TBAF (107 mg, 0.34 mmol) and AcOH (22 µL, 0.39 mmol) in THF (340 µL) was added. The reaction was stirred at -15° C for 10 min, then allowed to warm at r.t. and stirred for additional 30 min. The solution was diluted with water and extracted with CH_2Cl_2. The combined organic layers were dried over anhydrous Na_2SO_4, filtered and evaporated *in vacuo*. The crude product was purified with flash chromatography (petroleum ether–EtOAc 7:3) to give afford **12** as a red oil (250 mg, 87%).

^{1}H NMR (400 MHz, $CDCl_3$, 25 °C, TMS) δ 7.24 (d, $^3J_{H,H}$=8.7 Hz, 1H, 2x H-*ortho* PMB), 6.85 (d, $^3J_{H,H}$=8.6 Hz, 1H, 2x H-*meta* PMB), 5.71 (d, $^3J_{H,H}$=9.5 Hz, 1H, H-1), 5.34-5.23 (m, 2H, NH, H-3), 5.07 (t, $^3J_{H,H}$=9.9 Hz, 1H, H-4), 4.45 (s, 2H, CH_2-PMP), 4.32-4.24 (m, 1H, H-2), 4.19-4.11 (m, 1H, H-5), 3.79 (s, 3H, OCH_3), 3.55-3.38 (m, 1H, H-6a, H-6b), 3.02-2.84 (bs, 1H, OH), 2.22 (t, $^3J_{H,H}$=7.6 Hz, 2H, CH_2α-chain1), 2.18-2.05 (m, 4H, CH_2α-chain2,3), 1.68-1.41 (m, 6H, CH_2β-chains1,2,3), 1.24 (m, 60H, 30xCH_2), 0.88 (t, $^3J_{H,H}$=6.7 Hz, 9H, 3x CH_3-chains1,2,3).

^{13}C NMR (101 MHz, $CDCl_3$, 25 °C, TMS) δ 174.18, 173.02, 172.22, 159.29, 129.58, 113.73, 91.68, 73.18, 70.50, 68.85, 68.73, 55.22, 52.09, 36.73, 34.22, 34.11, 31.92, 29.68, 29.37, 29.28, 29.17, 25.59, 24.91, 22.69, 14.13.

HRMS (ESI): m/z [M+Na$^+$] calculated for $C_{56}H_{99}NNaO_9^+$: 952.7212. Found: 952.7217.

Compound 13

2-dodecanamido-2-deoxy-3,4-di-O-dodecanoyl 6-O-(4-methoxybenzyl)-β-D-glucopyranose (**13**). Compound **13** was synthesized starting from **11** accordingly to the procedure presented for compound **12** in 90% yield.

^{1}H NMR (400 MHz, $CDCl_3$, 25 °C, TMS) δ 7.23 (d, $^3J_{H,H}$=6.7 Hz, 2H, 2x H-*ortho* PMB), 6.85 (d, $^3J_{H,H}$=8.8 Hz, 2H, 2x H-*meta* PMB), 5.27 (d, $^3J_{H,H}$=9.9 Hz, 1H, H-1), 5.26-5.23 (m, 1H, NH, H-3), 5.05 (t, $^3J_{H,H}$=9.8 Hz, 1H, H-4), 4.44 (s, 2H, CH_2-PMP), 4.31-4.22 (m, 1H, H-2), 4.19-4.10 (m, 1H, H-5), 3.79 (s, 3H, OCH_3), 3.52-3.41 (m, 2H, H-6a, H-6b), 2.36-2.02 (m, 6H, CH_2α-chain1,2,3), 1.57-1.45 (m, 6H, CH_2β-chains1,2,3), 1.25 (m, 48H, 24xCH_2 chains), 0.87 (s, 9H, 3x CH_3-chains1,2,3).

^{13}C NMR (101 MHz, $CDCl_3$, 25 °C, TMS) δ 173.61, 172.45, 171.65, 158.71, 129.01, 113.16, 91.11, 72.61, 69.93, 68.28, 68.16, 54.65, 51.51, 36.16, 33.65, 33.54, 31.35, 29.10, 28.80, 28.71, 28.59, 25.01, 24.34, 22.12, 13.56.

HRMS (ESI): m/z [M+Na$^+$] calculated for $C_{50}H_{87}NNaO_9^+$: 868.6273. Found: 868.6278.

Compound 14

1-(dibenzyl)phosphor-2-tetradecanamido-2-deoxy-3,4-di-O-tetradecanoyl-6-O-(4-methoxybenzyl)-α-D-glucopyranose (**14**). Compound **12** (100 mg, 0.11 mmol) was dissolved in dry CH_2Cl_2 (1.8 mL). Imidazolium triflate (83 mg, 0.32 mmol) and dibenzyl *N*,*N*-diisopropyl phosphoramidite (108 µl, 0.32 mmol) were added, and the reaction was stirred for 30 min at r.t.. After cooling at -20°C, *m*CPBA (93 mg, 0.54 mmol) was added, the reaction was then stirred overnight at r.t.. Mixture was diluted with CH_2Cl_2, washed with a saturated solution of $NaHCO_3$ and brine. The organic layer was dried over anhydrous Na_2SO_4, filtered and to dryness. The crude product was purified with flash chromatography (petroleum ether–EtOAc 8:2) affording compound **14** as a brown solid (70 mg, 55%).

^{1}H NMR (400 MHz, $CDCl_3$, 25 °C, TMS) δ: 7.42-7.27 (m, 10H, 2x BnO-P), 7.17 (d, $^3J_{H,H}$=8.5 Hz, 2H, 2x H-*ortho* PMB), 6.80 (d, $^3J_{H,H}$=8.4 Hz, 2H, 2x H-*meta* PMB), 5.71 (dd, $^3J_{H,P}$=5.7, $^3J_{H,H}$=3.3 Hz, 1H, H-1), 5.57 (d, $^3J_{H,H}$= 9.1

Hz, 1H, NH), 5.24-5.14 (m, 2H, H-3, H-4), 5.11-4.95 (m, 4H, 2x CH_2(Ph)-O-P), 4.45-4.23 (m, 3H; CH_2-Ph PMB, H-2), 4.05 (m, 1H, H-5), 3.76 (s, 3H, OCH_3), 3.38 (d, $^3J_{H,H}$=3.4 Hz, 2H, H6a, H6b), 2.21 (t, $^3J_{H,H}$=7.6 Hz, 2H, CH_2α-chain1), 2.14 (t, $^3J_{H,H}$=7.5 Hz, 2H, CH_2α-chain2), 1.85 (m, 2H, CH_2α-chain3), 1.50 (m, 4H, CH_2β-chains1,2), 1.46-1.36 (m, 2H, CH_2β-chain3), 1.27 (m, 60H, 30xCH_2 chains), 0.88 (t, $^3J_{H,H}$=6.6 Hz, 9H, 3x CH_3 chains).

^{13}C NMR (101 MHz, $CDCl_3$, 25 °C, TMS) δ 184.72, 174.06, 171.63, 139.76, 129.49, 128.71, 127.97, 113.65, 92.90, 75.02, 73.14, 71.00, 69.92, 69.78, 69.68, 69.29, 69.23, 67.79, 67.75, 55.18, 36.29, 34.14, 31.92, 29.67, 29.51, 29.16, 25.36, 24.84, 22.69, 14.13.

HRMS (ESI): m/z [M+Na^+] calculated for $C_{70}H_{112}NNaO_{12}P^+$: 1212.7814. Found: 1212.7817.

Compound 15

1-(dibenzyl)phosphor-2-dodecanamido-2-deoxy-3,4-di-O-dodecanoyl-6-O-(4-methoxybenzyl)-α-D-glucopyranose (**15**). Compound **15** was synthesized starting from **13** accordingly to the procedure presented for compound **14** in 55% yield.

^{1}H NMR (400 MHz, $CDCl_3$, 25 °C, TMS) δ: 7.36-7.31 (m, 10H, 2x BnO-P), 7.16 (d, $^3J_{H,H}$=8.5 Hz, 2H, 2x H-*ortho* PMB), 6.79 (d, $^3J_{H,H}$=8.6 Hz, 2H, 2x H-*meta* PMB), 5.70 (dd, $^3J_{H,P}$=6.0, $^3J_{H,H}$=3.2 Hz, 1H, H-1), 5.57 (d, $^3J_{H,H}$= 9.1 Hz, 1H, NH), 5.21-5.15 (m, 2H, H-3, H-4), 5.11-4.91 (m, 4H, 2x CH_2(Ph)-O-P), 4.70 (s, 3H; CH_2-Ph PMB, H-2), 4.04 (s, 1H, H-5), 3.76 (s, 3H, OCH_3), 3.52-3.34 (m, 2H, H6a, H6b), 2.21 (t, $^3J_{H,H}$=7.7 Hz, 2H, CH_2α-chain1), 2.14 (t, $^3J_{H,H}$=7.5 Hz, 2H, CH_2α-chain2), 1.92-1.78 (m, 2H, CH_2α-chain3), 1.57-1.44 (m, 6H, CH_2β-chains1,2,3), 1.26-1.23 (m, 48H, 24xCH_2 chains), 0.88 (t, $^3J_{H,H}$=6.2 Hz, 9H, 3x CH_3 chains).

^{13}C NMR (101 MHz, $CDCl_3$, 25 °C, TMS) δ 174.05, 173.01, 171.85, 129.48, 128.71, 127.93, 113.65, 93.67, 75.01, 73.14, 70.99, 69.78, 67.76, 55.17, 31.93, 31.92, 29.67, 29.51, 29.37, 29.30, 29.23, 29.16, 25.36, 25.36, 24.84, 24.84, 22.70, 22.69, 22.69, 14.13, 14.13, 14.11.

HRMS (ESI): m/z [M+Na^+] calculated for $C_{64}H_{100}NNaO_{12}P^+$: 1128.6875. Found: 1128.6880.

FP11

1-phospho-2-tetradecanamido-2-deoxy-3,4-di-O-tetradecanoyl-α-D-glucopyranose (sodium salt) (**FP11**) Compound **14** (25 mg, 0.021 mmol) was dissolved in degassed MeOH (1.3 mL), and 3 mg (10% weight) of 10% Pd-C was added under Ar atmosphere. The reaction mixture was stirred at r.t. under H_2 atmosphere overnight. Triethylamine (80 µL) was added to the reaction mixture, and the suspension was filtered with a syringe filter and evaporated to dryness. The resulting solid was dissolved in CH_2Cl_2/MeOH 1:2 (3 mL) and treated first with Amberlite IRA 120 H^+ exchange resin and then with IR 120 Na^+, giving compound **FP11** as a white solid (16 mg, 82%).

^{1}H NMR (400 MHz, CD_3OD, 25 °C, TMS) δ: 5.47 (dd, $^3J_{H,P}$=6.7, $^3J_{H,H}$=3.2 Hz, 1H; H-1), 5.34 (t, $^3J_{H,H}$=10.0 Hz, 1H, H-3), 5.09 (t, $^3J_{H,H}$=9.9 Hz, 1H, H-4), 4.28 (dd, $^3J_{H,H}$=10.8, 2.5 Hz, 1H, H-2), 4.14-4.06 (m, 1H, H-5), 3,66 (m, 1H, H-6a), 3,56 (m, 1H, H-6b), 2.39-2.10 (m, 6H, 3x CH_2α-chains), 1.55 (s, 6H, 3x CH_2β-chains), 1.29 (m, 60H, 30xCH_2 chains), 0.89 (t, $^3J_{H,H}$=6.6 Hz, 9H, 3x CH_3-chains).

^{13}C NMR (101 MHz, $CDCl_3$, 25 °C, TMS) δ 174.72, 172.78, 131.63, 109.99, 92.22, 73.52, 72.09, 66.78, 62.07, 59.75, 48.57, 38.68, 34.14, 33.96, 32.77, 31.96, 29.88, 29.80, 29.71, 29.67, 29.43, 29.29, 24.89, 22.72, 14.14, 1.03.

ESI-MS: $[M]^-$ m/z =888.6; found: m/z =888.7.

FP18

1-phospho 2-dodecanamido-2-deoxy 3,4-di-O-dodecanoyl-α-D-glucopyranose (sodium salt) (**FP18**). **FP18** was synthesized starting from **15** accordingly to the procedure presented for **FP11** in 80% yield.

^{1}H NMR (400 MHz,_CD_3OD, 25 °C, TMS) δ: 5.57 (dd, $^3J_{H,P}$=6.6, $^3J_{H,H}$=3.4 Hz, 1H; H-1), 5.35 (dd, $^3J_{H,H}$=10.8 Hz, $^3J_{H,H}$=9.4 Hz, 1H, H-3), 5.16 (t, $^3J_{H,H}$=9.8 Hz, 1H, H-4), 4.34 (dt, $^3J_{H,H}$=10.9, 3.2 Hz, 1H, H-2), 4.12-4.05 (m, 1H, H-5), 3.69 (dd, $^3J_{H,H}$=12.4, $^3J_{H,H}$=2.4 Hz, 1H, H-6a), 3,58 (dd, $^3J_{H,H}$=12.3, $^3J_{H,H}$=4.4 Hz, 1H, H-6b), 2.41-2.17 (m, 6H, 3x CH_2α-chains), 1.57 (d, *J* =8.3 Hz, 6H, 3x CH_2β-chains), 1.32 (s, 48H, 24xCH_2 chains), 0.92 (t, $^3J_{H,H}$=6.5 Hz, 9H, 3x CH_3-chains)

^{13}C NMR (101 MHz, CD_3OD, 25 °C, TMS) δ δ 175.08, 173.14, 172.51, 94.56, 71.23, 70.64, 68.48, 60.27, 51.72, 51.72, 51.64, 35.65, 33.73, 31.67, 29.36, 29.32, 29.28, 29.24, 29.21, 29.17, 29.14, 29.10, 28.83, 25.55, 24.52, 22.31, 13.01, 13.01.

ESI-MS: $[M]^-$ m/z =804.5396; found: m/z =804.5401.

Compound 16

2-tetradecanamido-2-deoxy-3,4-di-O-tetradecanoyl-β-D-glucopyranose (**16**). Compound **13** (120 mg, 0.129 mmol) was dissolved in degassed MeOH (8 mL), and 12 mg (10% weight) of 10% Pd-C was added under Ar atmosphere. The reaction mixture was stirred at r.t. under H_2 atmosphere overnight. After Catalyst removal by filtration on Celite®, solvent was evaporated *in vacuo* giving compound **16** as a white solid (100 mg, 95%).

^{1}H-NMR: (400 MHz, $CDCl_3$, 25 °C, TMS): δ 6.09 (d, $^3J_{H,H}$=9.1 Hz, 1H, H-1α), 5.34 (t, $^3J_{H,H}$ =10.1 Hz, 1H, H-3), 5.02 (t, $^3J_{H,H}$=9.7 Hz, 1H, H-4), 4.21 (t, $^3J_{H,H}$=9.1 Hz, 1H, H-2), 4.04 (m, 1H, H-5), 3.67 (m, 1H, H-6a), 3.56 (m, 1H, H-6b), 2,25 (m, 4H, CH_2α-chains1,2), 2,11 (m, 4H, CH_2α-chain3), 1.52 (m, 6H, CH_2β-chains1,2,3), 1.23 (m, 60H, 30xCH_2), 0.86 (t, $^3J_{H,H}$=6.6 Hz, 9H, CH_3-chains1,2,3).

^{13}C NMR (101 MHz, $CDCl_3$, 25 °C, TMS): δ 174.15, 173.64, 173.17, 91.38, 70.30, 69.67, 68.57, 68.47, 61.17, 52.46, 36.71, 34.20, 34.15, 31.92, 31.88, 29.73, 29.71, 29.68, 29.56, 29.55, 29.49, 29.41, 29.38, 29.37, 29.30, 29.20, 29.17, 25.61, 24.97, 24.93, 22.70, 22.68, 14.12, 14.11.

HRMS (ESI): m/z [M+Na$^+$] calculated for $C_{48}H_{91}NNaO_8^+$: 832.6637. Found: 832.6639.

Compound 17

1,6-bis(dibenzyl)phospho-2-tetradecanamido-2-deoxy-3,4-di-O-tetradecanoyl-α-D-glucopyranose (**17**). Compound **16** (50 mg, 0.062 mmol) was dissolved in dry CH_2Cl_2 (1.0 mL), then imidazolium triflate (72 mg, 0.28 mmol) and dibenzyl *N*,*N*-diisopropyl phosphoramidite (91 µl, 0.27 mmol) were added. The reaction was stirred at r.t. for 1,5 h. The solution was then cooled at 0°C and *m*CPBA (85 mg, 0.49 mmol) was added. After stirring at r.t. overnight, the mixture was diluted with CH_2Cl_2, washed with a saturated solution of $NaHCO_3$ and brine. The organic layer was dried over anhydrous Na_2SO_4, filtered and evaporated *in vacuo*. The crude product was purified with flash chromatography (petroleum ether–EtOAc 8:2) affording compound **17** as a brown solid (36 mg, 44%).

^{1}H-NMR: (400 MHz, $CDCl_3$, 25 °C, TMS): δ 7.44-7.21 (m, 20H, 4x Ar-H), 5.64 (dd, $^3J_{H,H}$=6.0, 3.3 Hz, 1H, H-1β), 5.61 (d, $^3J_{H,H}$=9.3 Hz, 1H, NH), 5.20-5.10 (m, 2H, H-3, H-5), 5.10-4.96 (m, 9H, 4x CH2-Ph, H-4), 4.33 (t, $^3J_{H,H}$=9.8

Hz, 1H, H-2), 3.92 (m, 2H, H-6a, H-6b), 2,20 (m, 4H, $CH_2\alpha$-chains1,2), 1.95-1.77 (m, 2H, $CH_2\alpha$-chain3), 1.49 (m, 4H, $CH_2\beta$-chains1,2), 1.47-1.36 (m, 2H, $CH_2\beta$-chain3), 1.27 (m, 60H, 30xCH_2), 0.88 (t, $^3J_{H,H}$=6.6 Hz, 9H, CH_3-chains1,2,3).

^{13}C NMR (101 MHz, $CDCl_3$, 25 °C, TMS) δ 173.95, 171.72, 151.63, 128.86, 128.77, 128.73, 128.54, 128.53, 128.49, 128.47, 128.10, 128.03, 127.96, 127.89, 109.99, 96,23, 88,76, 88,44, 70.16, 69.88, 69.71, 69.44, 69.38, 66.67, 34.11, 31.92, 29.67, 29.51, 29.37, 29.31, 29.24, 29.16, 25.39, 24.85, 22.69, 14.13, 14.12.

HRMS (ESI): m/z [MNa^+] calculated for $C_{76}H_{117}NNaO_{14}P_2^+$: 1352.7842. Found: 1352.7848.

FP111

1,6-bisphospho-2-tetradecanamido-2-deoxy-3,4-di-O-tetradecanoyl-α-D-glucopyranose (sodium salt) (**FP111**) Compound **17** (33 mg, 0.028 mmol) was dissolved in degassed MeOH (1.3 mL), and 4 mg (10% weight) of 10% Pd-C was added under Ar atmosphere. The reaction mixture was stirred at r.t. under H_2 atmosphere overnight. Triethylamine (80 µL) was added to the reaction mixture, and the suspension was filtered with a syringe filter and evaporated to dryness. The resulting solid was dissolved in CH_2Cl_2/MeOH 1:2 (3 mL) and treated first with Amberlite IRA 120 H^+ exchange resin and then with IR 120 Na^+, giving compound **FP111** as a white solid (20 mg, 68%).

^1H-NMR: (400 MHz, CD3OD, 25 °C, TMS): 5.53 (dd, $^3J_{H,H}$=6.3, 3.2 Hz, 1H, H-1β), 5.32 (t, $^3J_{H,H}$=10.8 Hz, 1H, H-3), 5.14 (t, $^3J_{H,H}$=9.8 Hz, 1H, H-4), 4.32 (dt, $^3J_{H,H}$=10.9, 3.1 Hz, 1H, H-2), 4.28-4.21 (m, 1H, H-5), 4.12-3.97 (m, 2H, H-6a, H-6b), 2.48-2.06 (m, 6H, $CH_2\alpha$-chains1,2,3), 1.71-1.47 (m, 6H, $CH_2\beta$-chains1,2,3), 1.29 (s, 60H, 30xCH_2), 0.90 (t, $^3J_{H,H}$=6.6 Hz, 9H, CH_3-chains1,2,3).

^{13}C NMR (101 MHz, CD3OD, 25 °C, TMS) δ 210.68, 175.10, 172.94, 172.30, 127.68, 89.22, 85,74, 70.50, 69.44, 69.37, 68.31, 51.52, 48.20, 35.57, 33.65, 33.46, 31.71, 31.68, 29.47, 29.45, 29.28, 29.20, 29.12, 29.10, 29.02, 28.85, 28.80, 25.61, 24.50, 24.45, 22.35, 13.06, 13.05, -5.54.

ESI-MS: $[M+H]^+$ m/z =968.6; found: m/z =968,7.

NMR spectra

Fig. S5: Compound 8. ^{1}H NMR and ^{13}C APT NMR spectra in $CDCl_3$

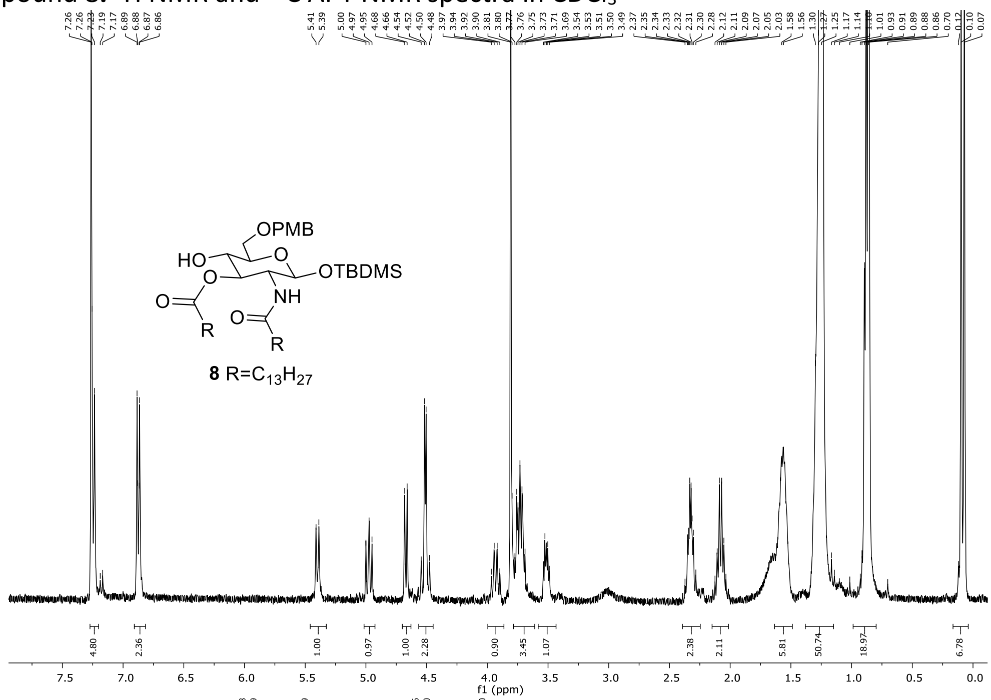

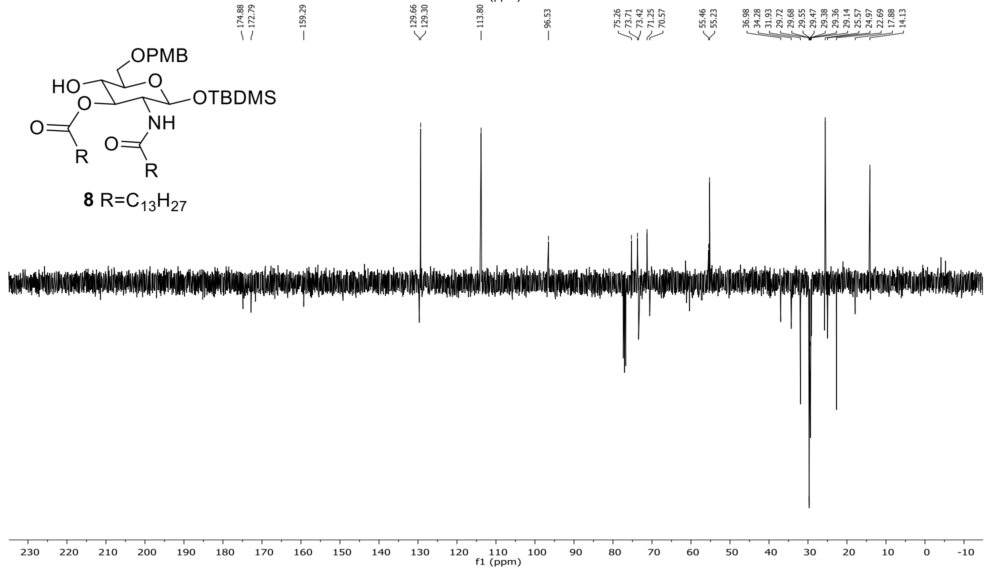

Fig. S6:Compound 10. ^{1}H NMR and ^{13}C APT NMR spectra in $CDCl_3$

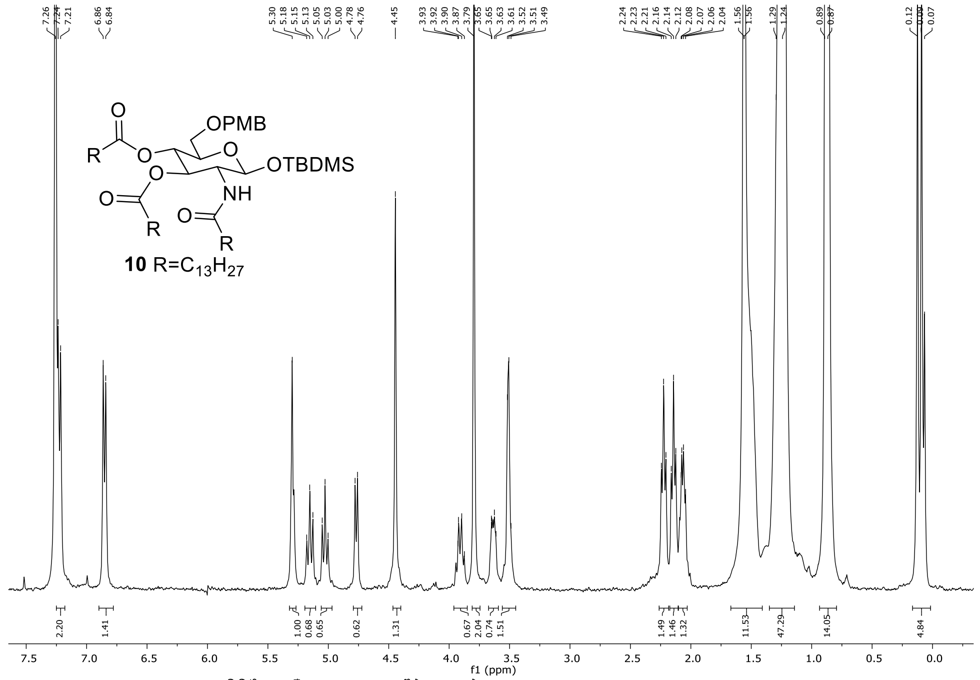

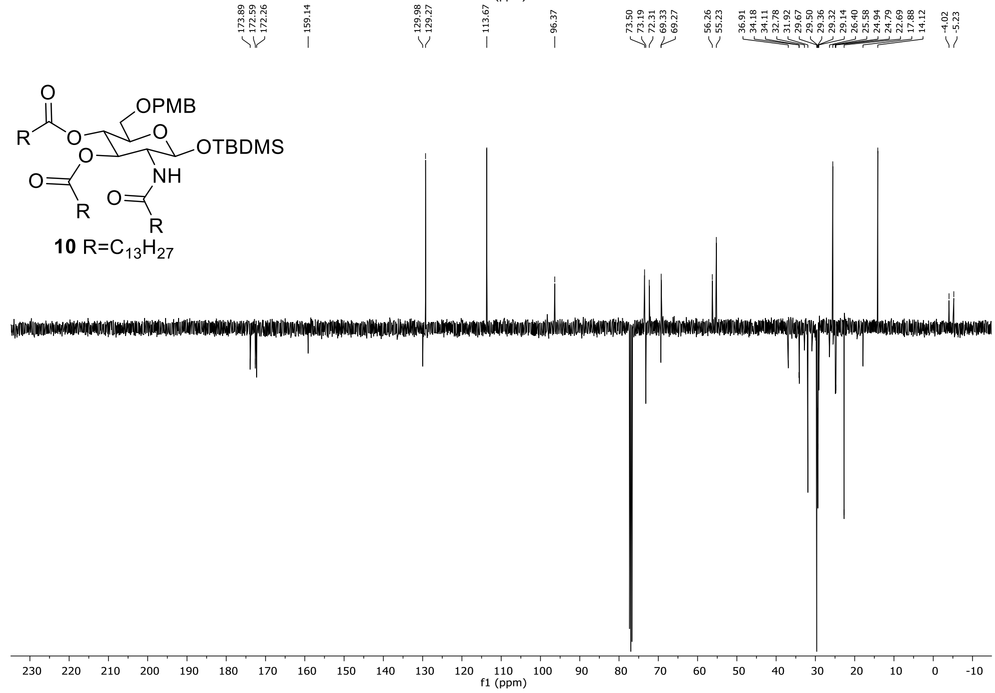

Fig. S7: Compound 12. ^{1}H NMR and ^{13}C APT NMR spectra in $CDCl_3$

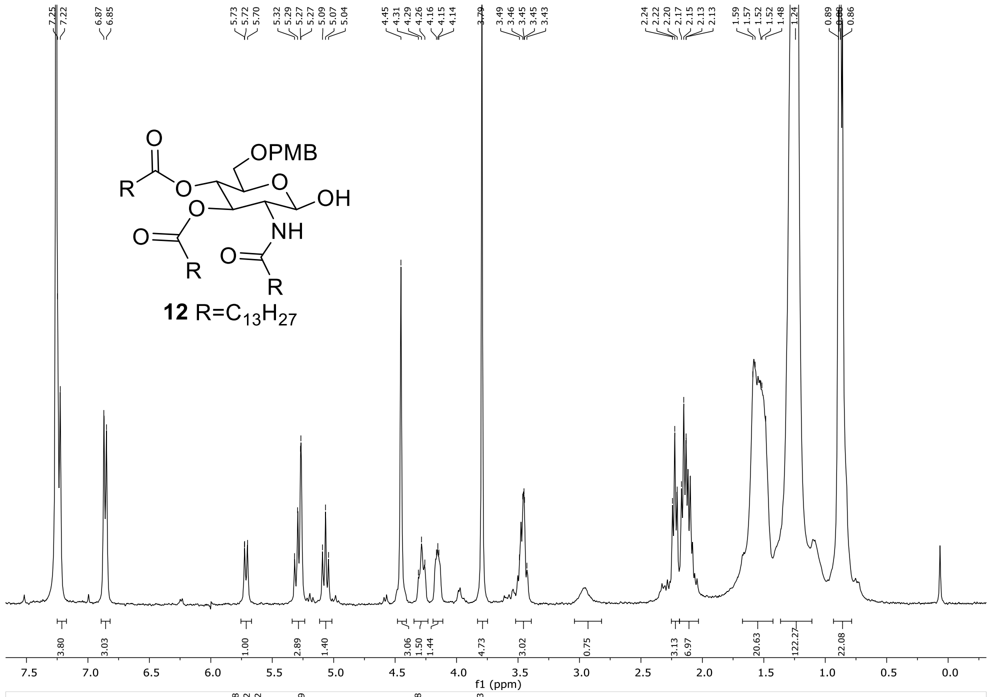

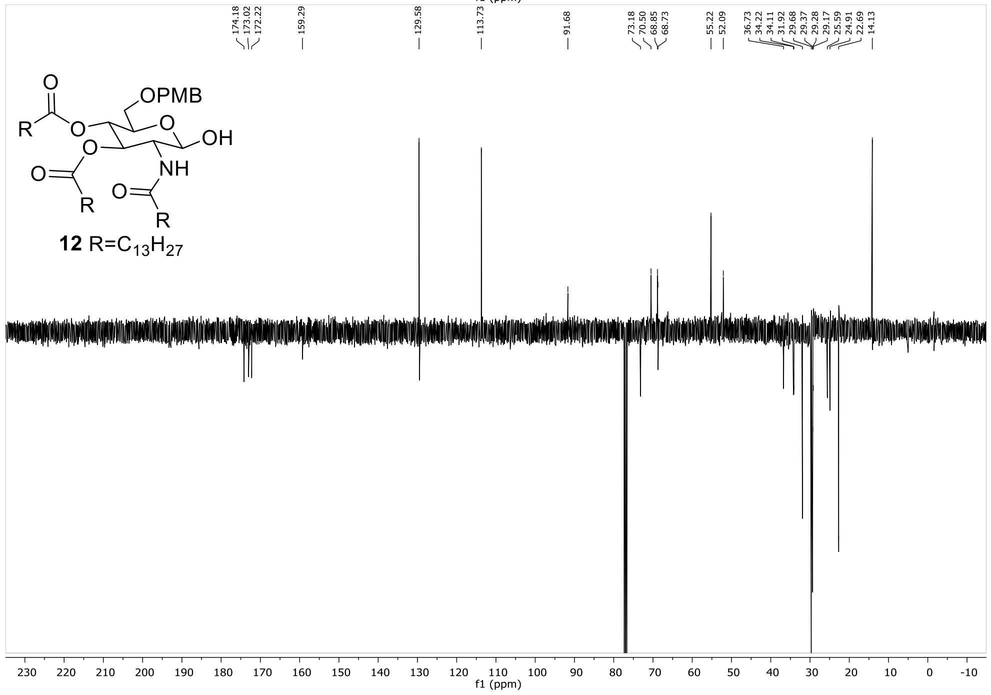

Fig. S8: Compound 14. ^{1}H NMR and ^{13}C APT NMR spectra in $CDCl_3$

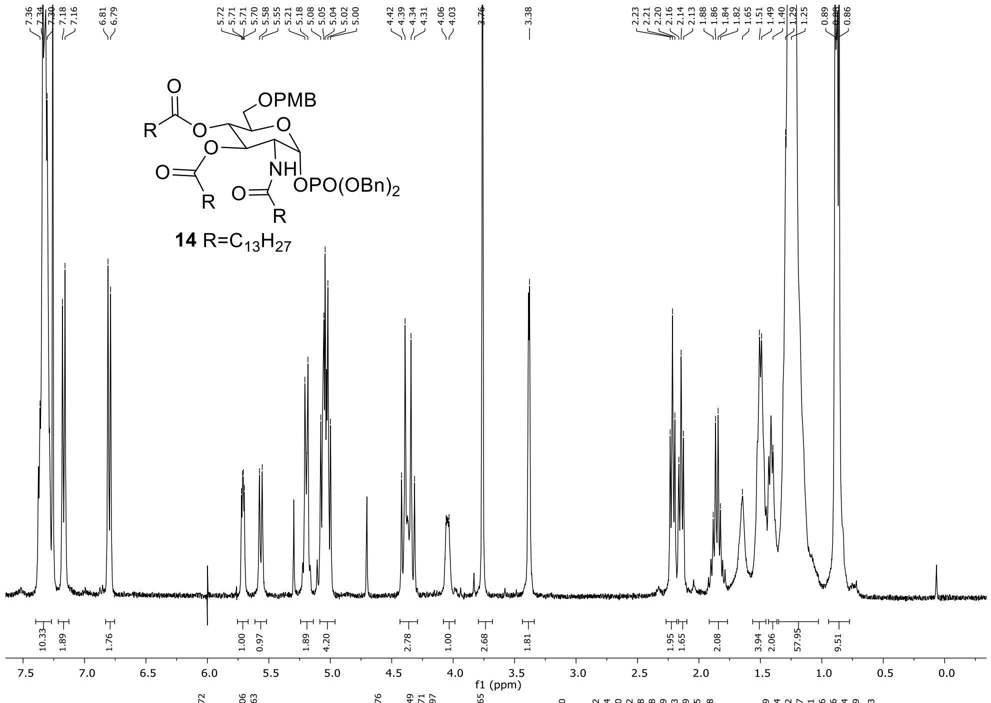

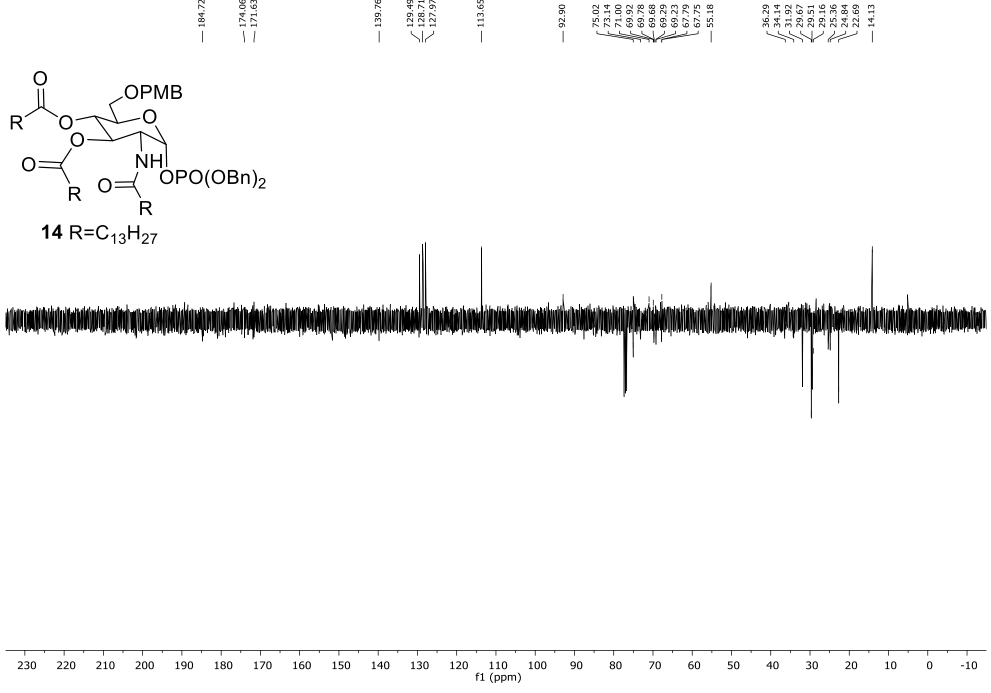

Fig. S9: Compound FP11. ^{1}H NMR and ^{13}C APT NMR spectra in CD_3OD

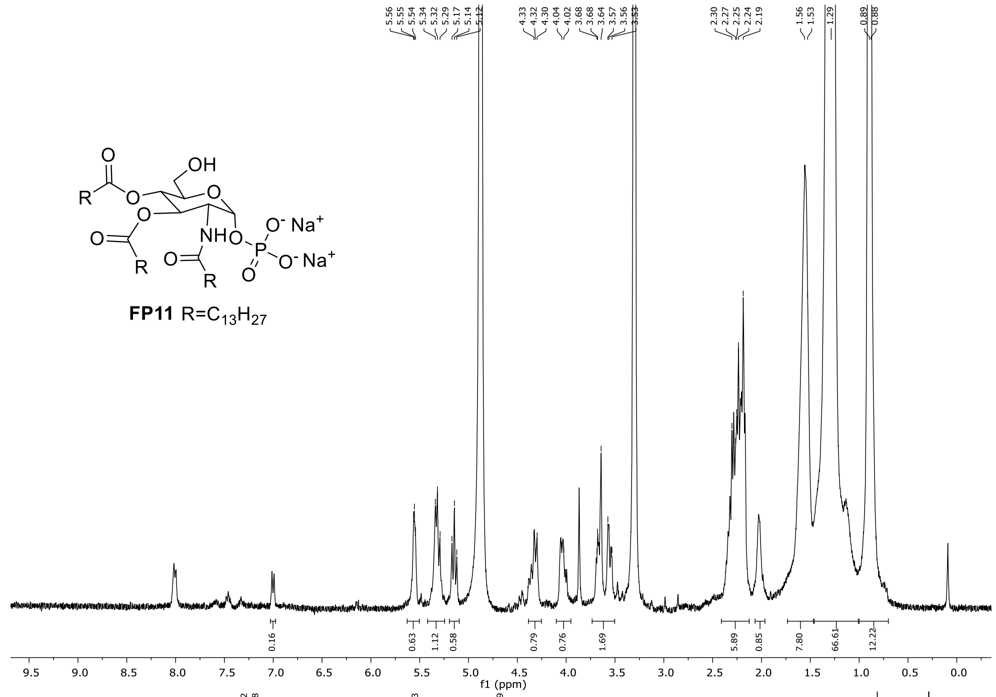

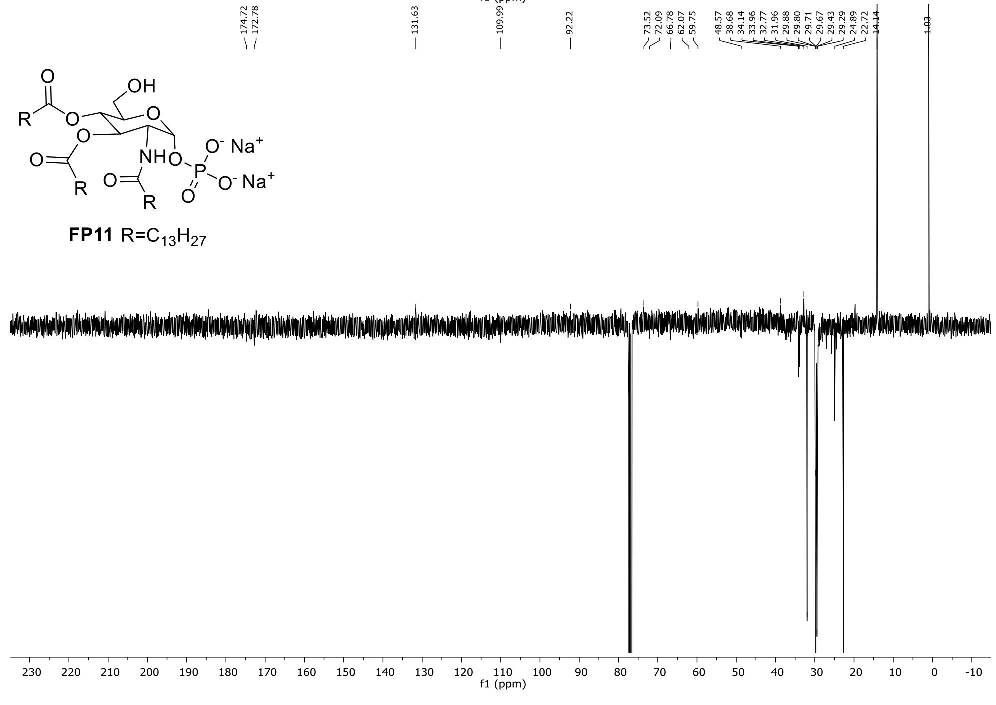

Fig. S10: Compound 9. ^{1}H NMR and ^{13}C APT NMR spectra in $CDCl_3$

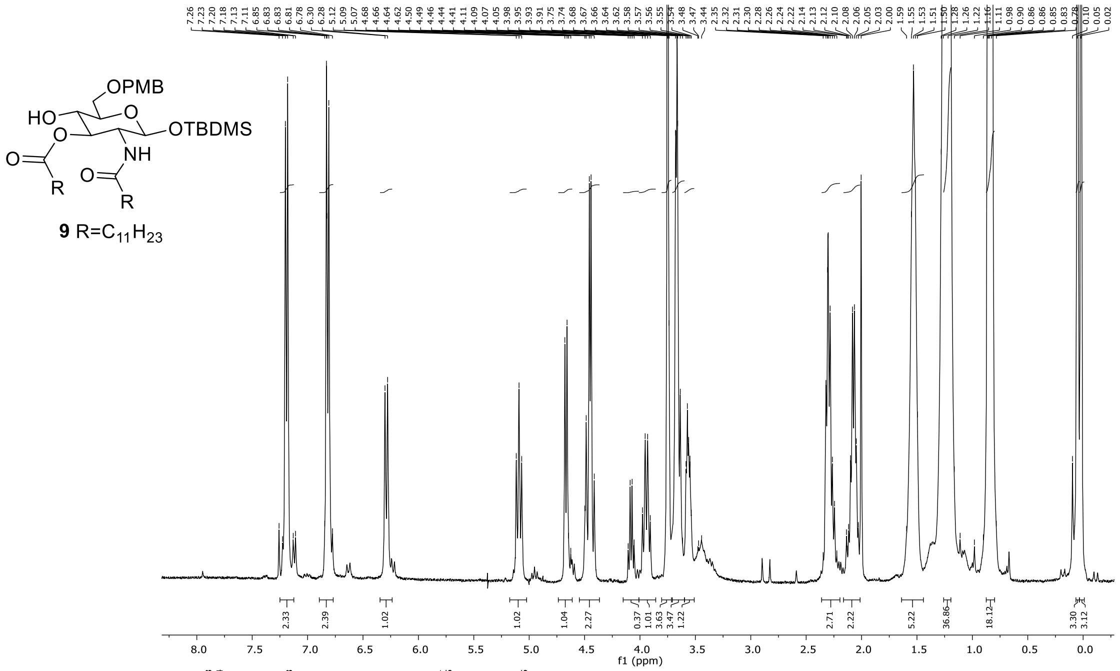

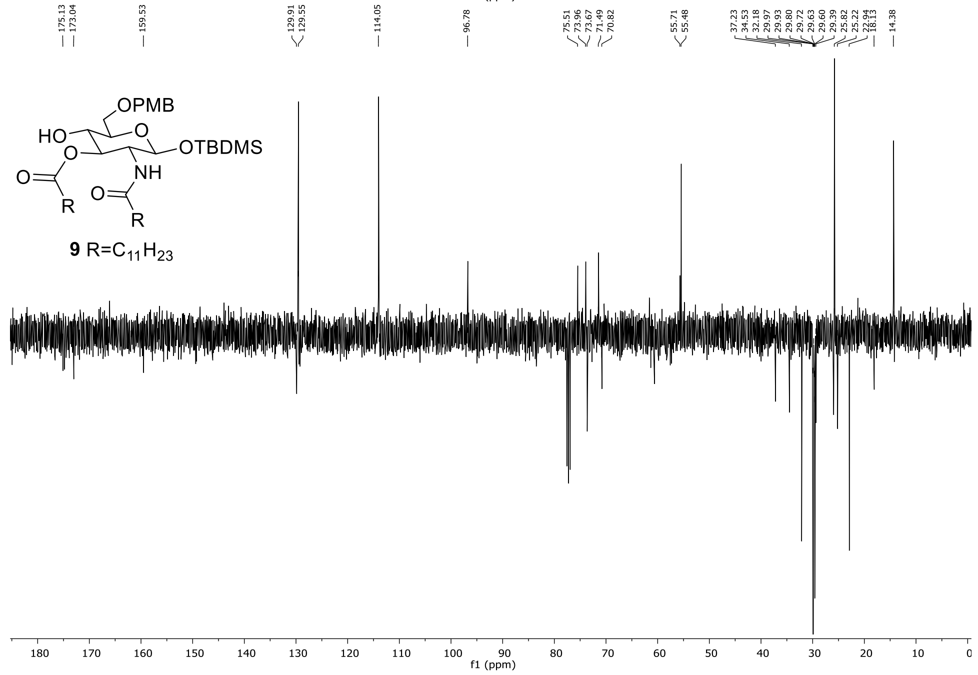

Fig. S11: Compound 11. ^{1}H NMR and ^{13}C APT NMR spectra in $CDCl_3$

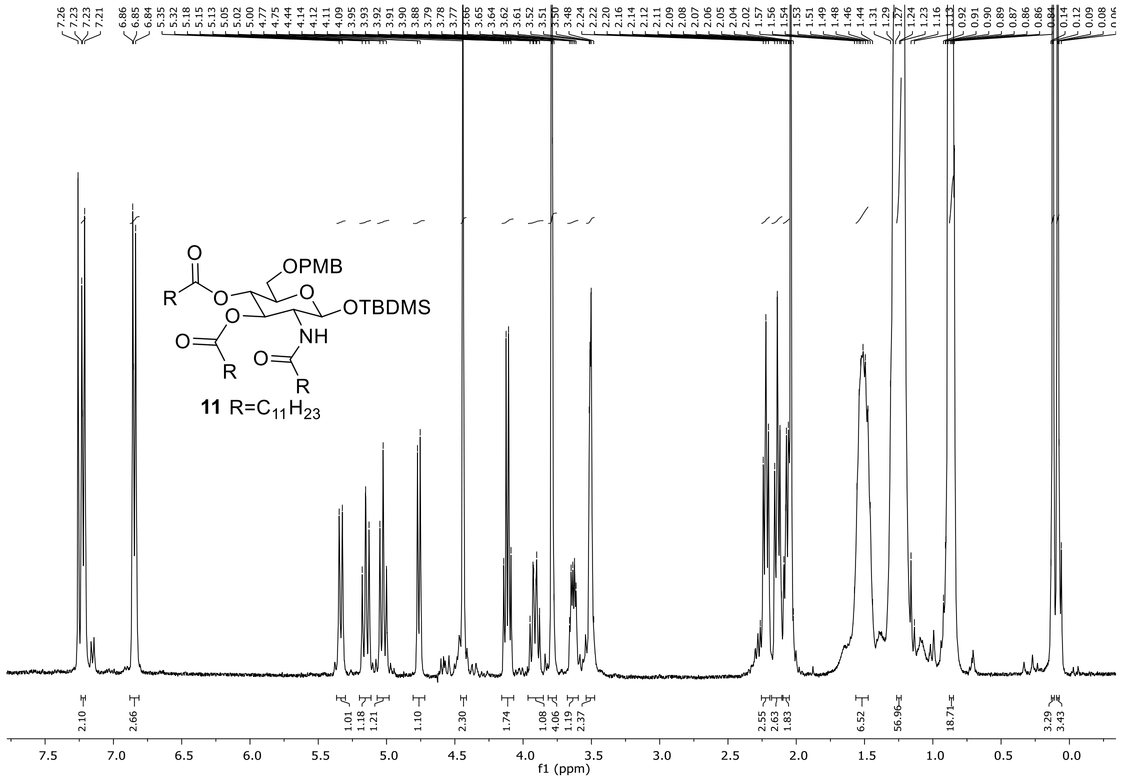

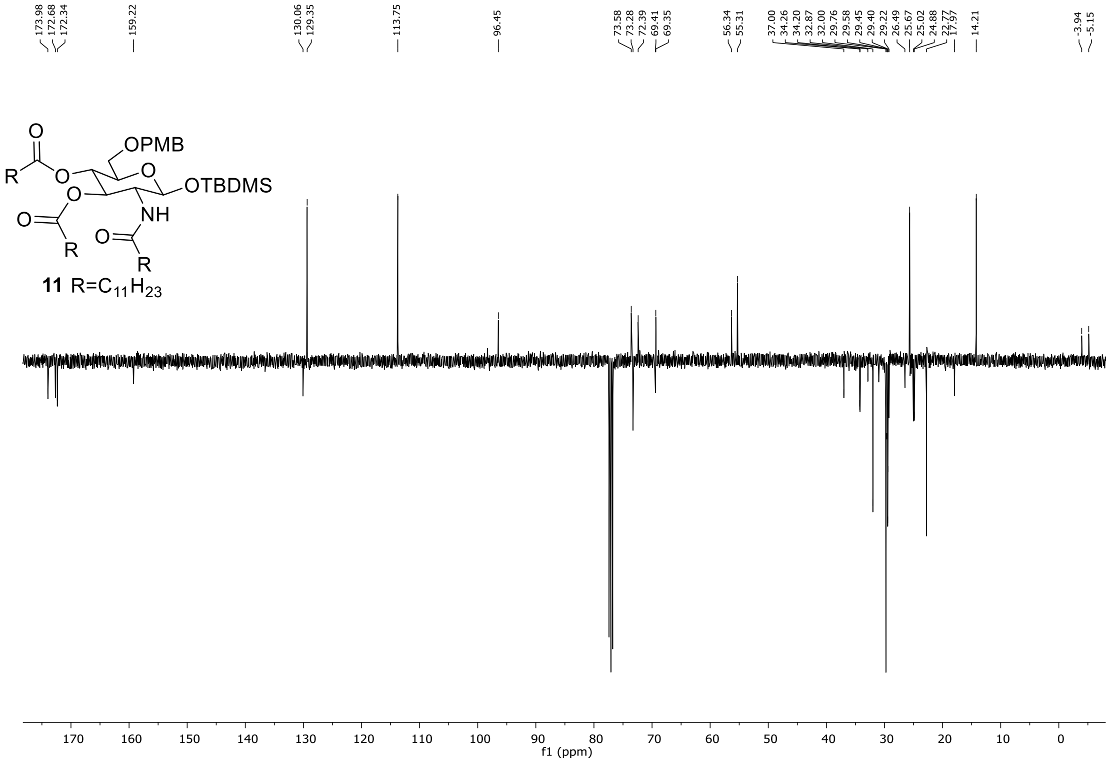

Fig. S12: Compound 13. ^{1}H NMR and ^{13}C APT NMR spectra in $CDCl_3$

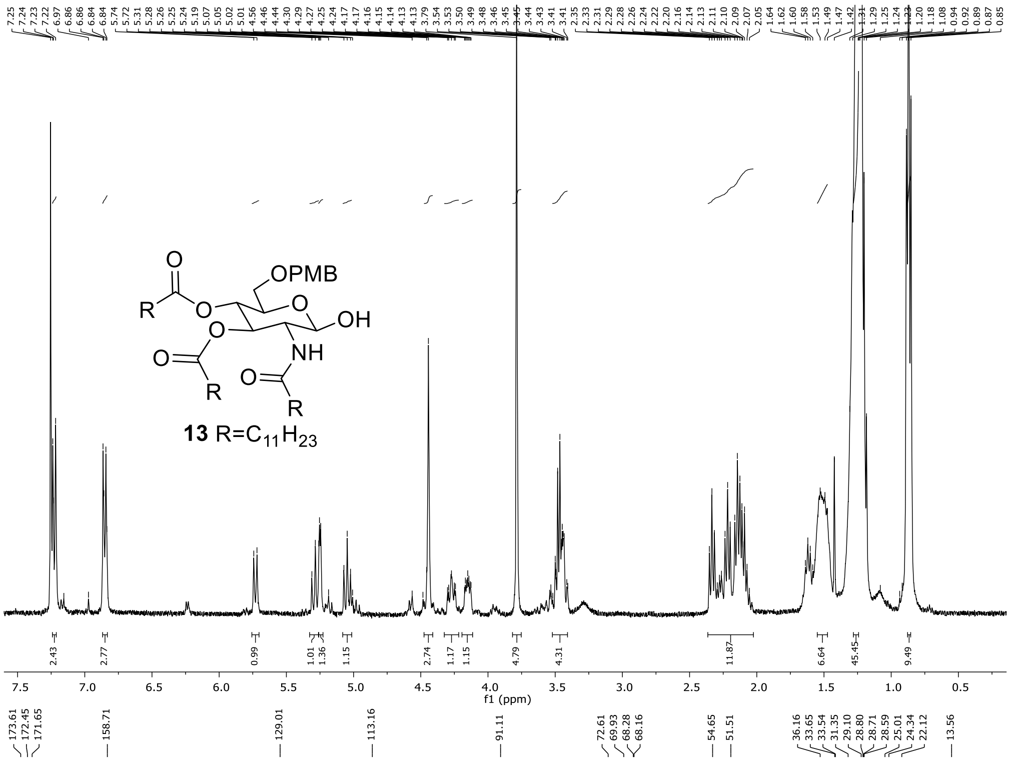

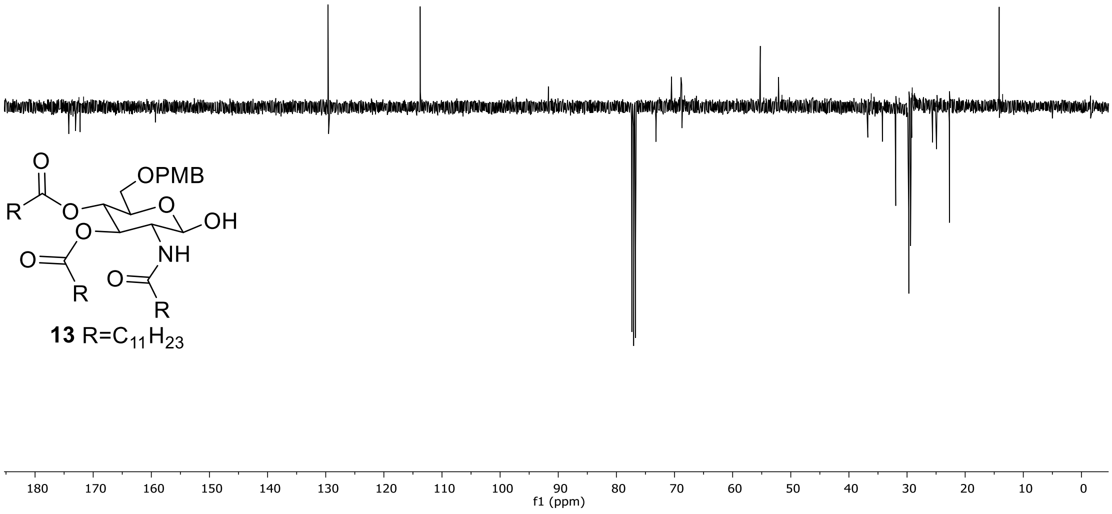

Fig. S13: Compound 15. ^{1}H NMR and ^{13}C APT NMR spectra in $CDCl_3$

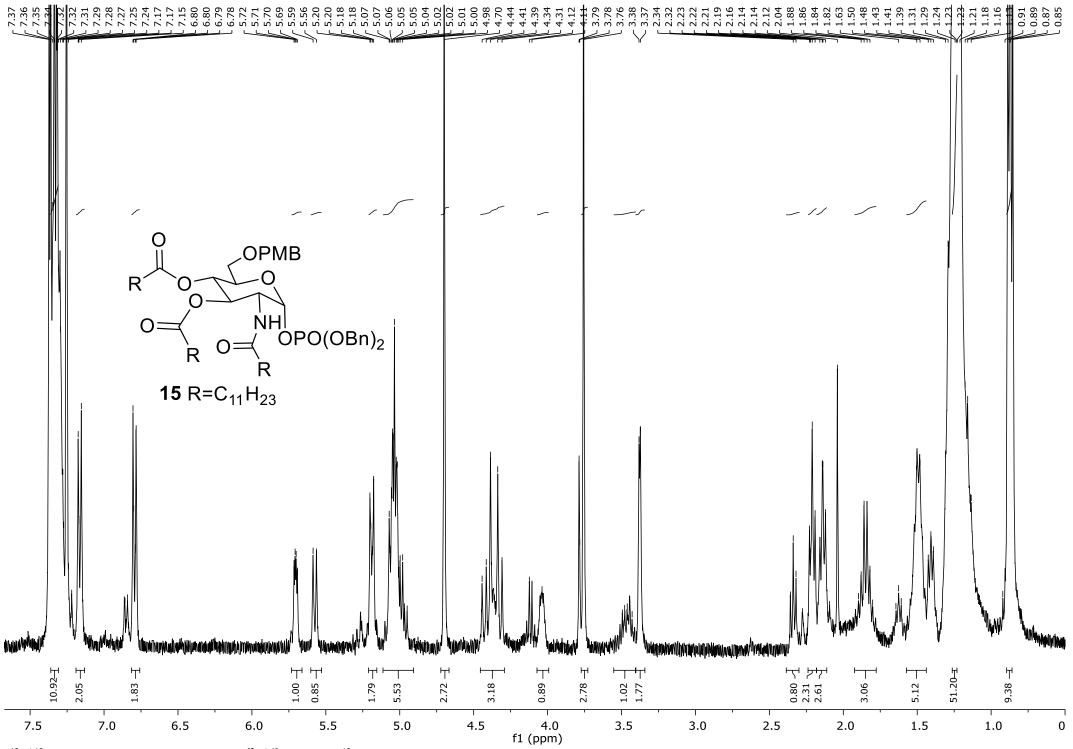

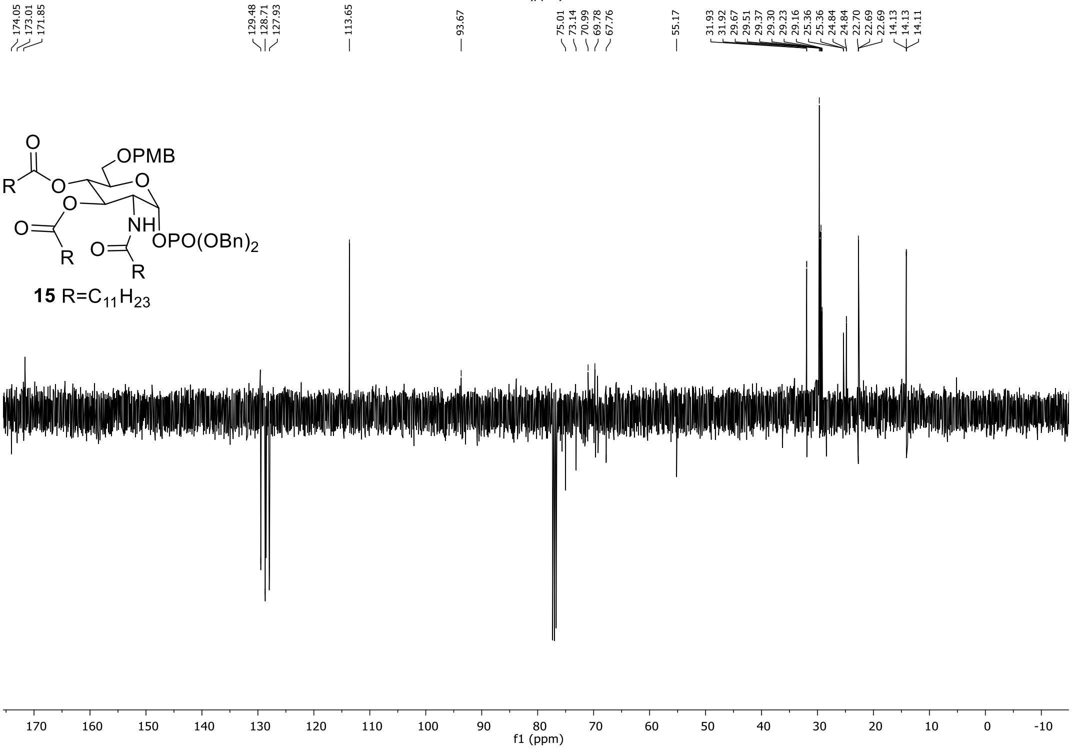

Fig. S14: Compound FP18. ^{1}H NMR and ^{13}C APT NMR spectra in CD_3OD

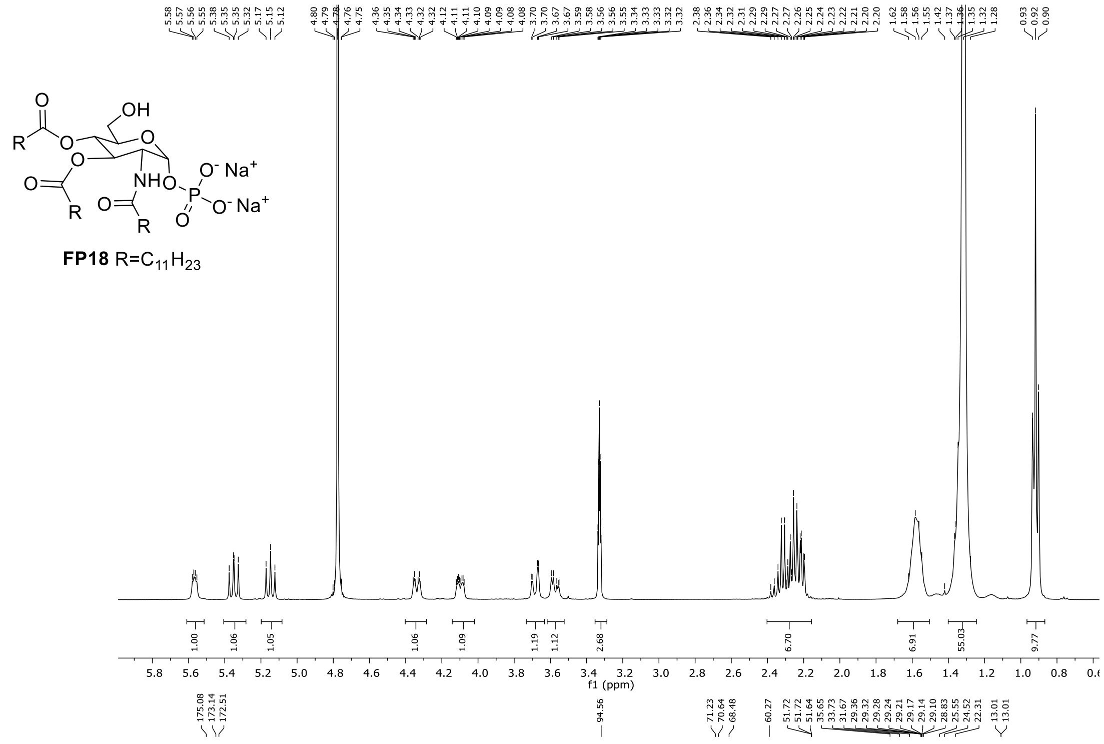

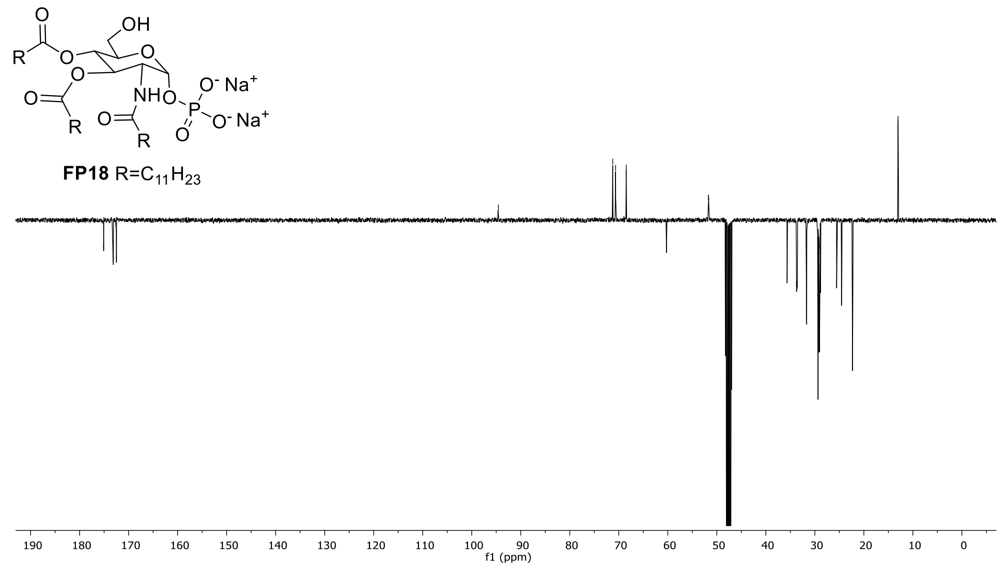

Fig. S15: Compound 16. ^{1}H NMR and ^{13}C APT NMR spectra in $CDCl_3$

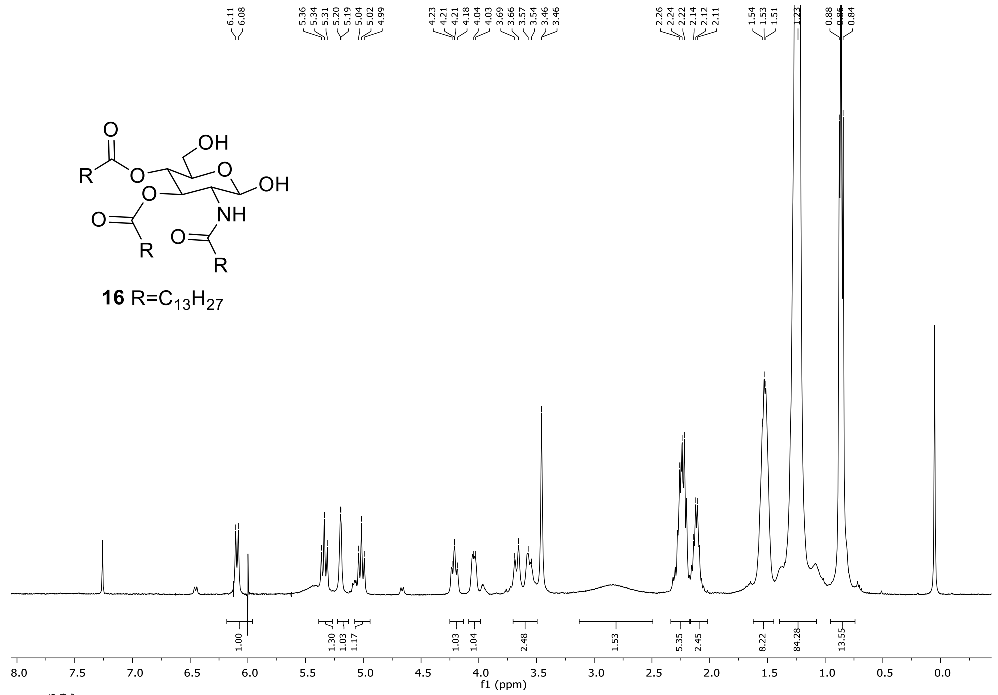

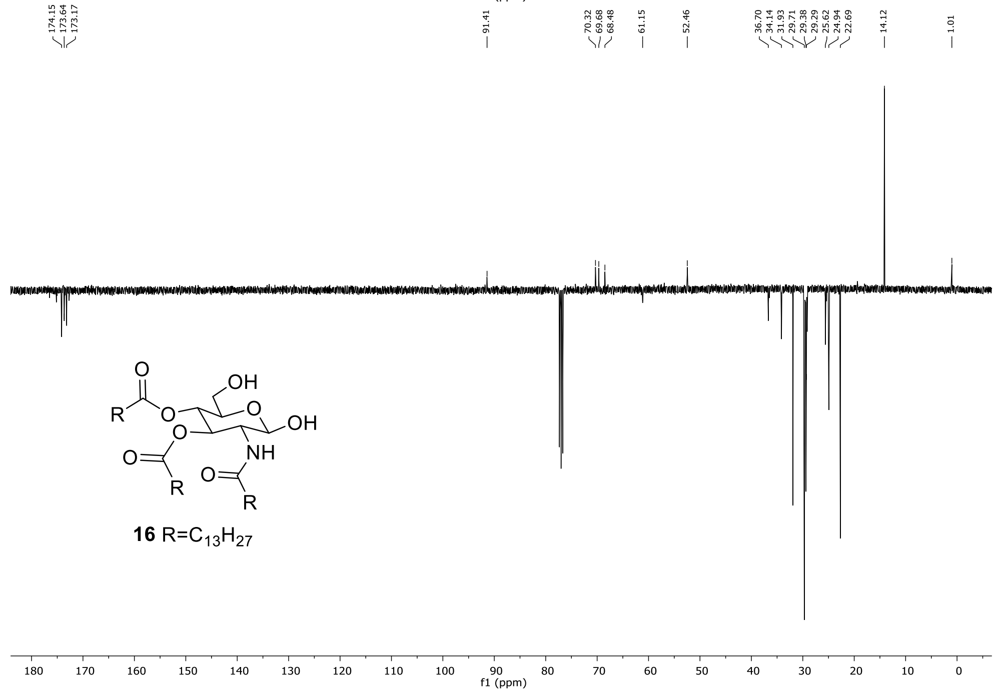

Fig. S16: Compound 17. ^{1}H NMR and ^{13}C APT NMR spectra in $CDCl_3$

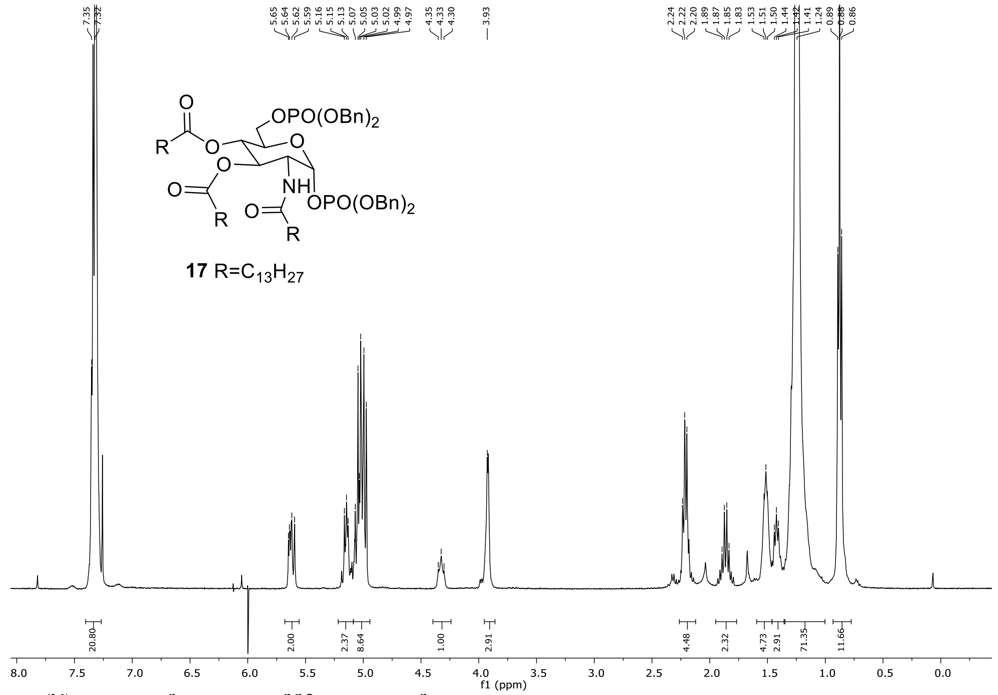

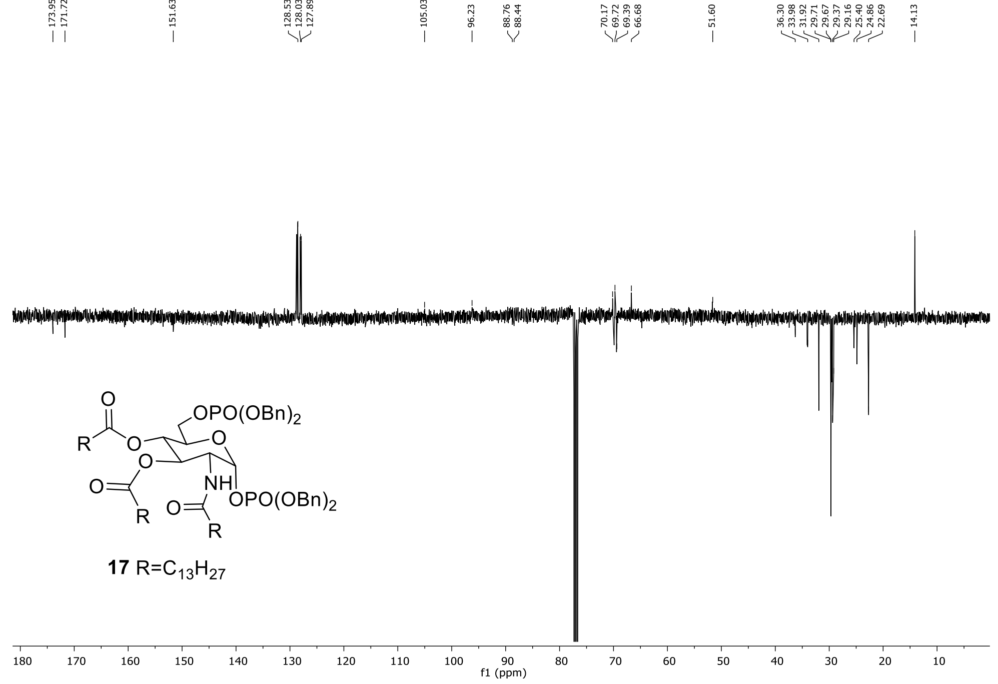

Fig. S17: Compound FP111. ^{1}H NMR and ^{13}C APT NMR spectra in CD_3OD

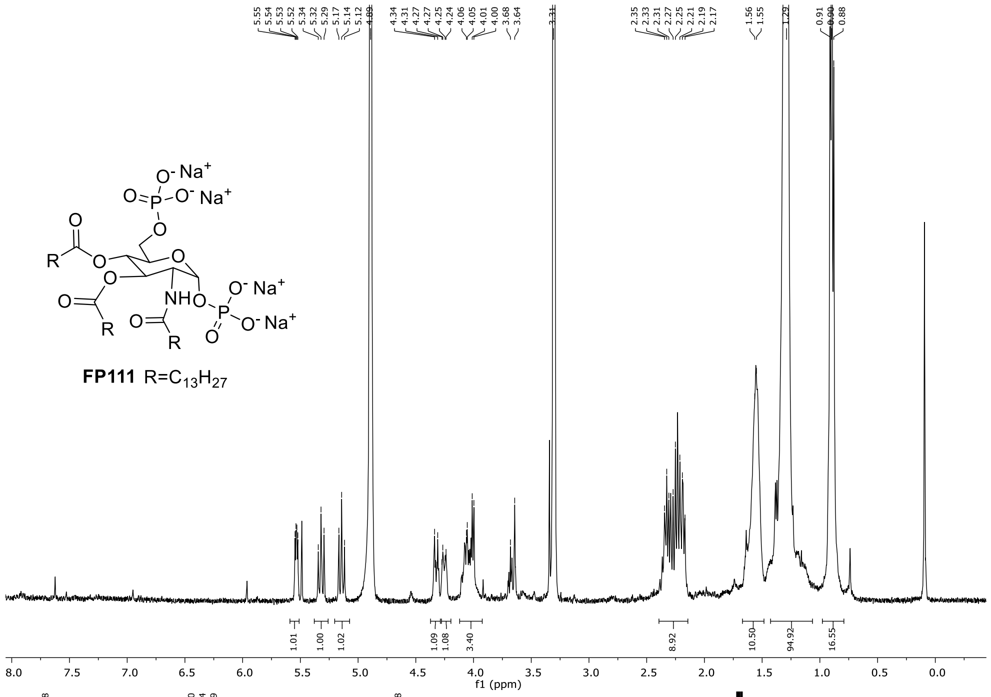

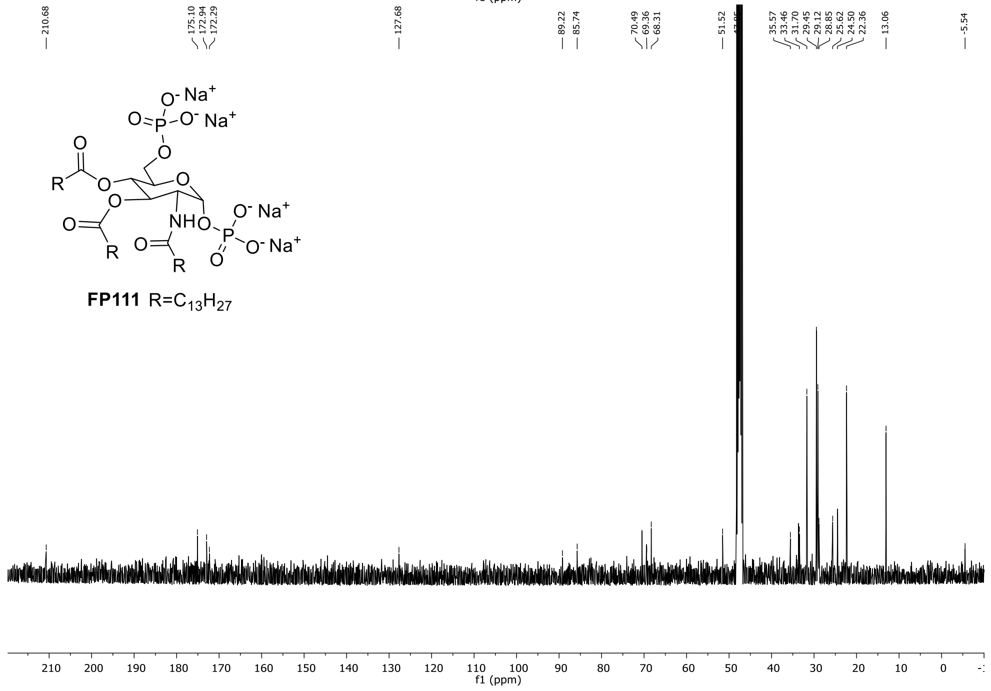

In vitro evaluation of the cytotoxicity of compounds FP11 and FP18

THP1-derived macrophages were exposed to increasing concentration of compounds FP11 and FP18. FPs molecules do not affect THP-1 macrophage viability

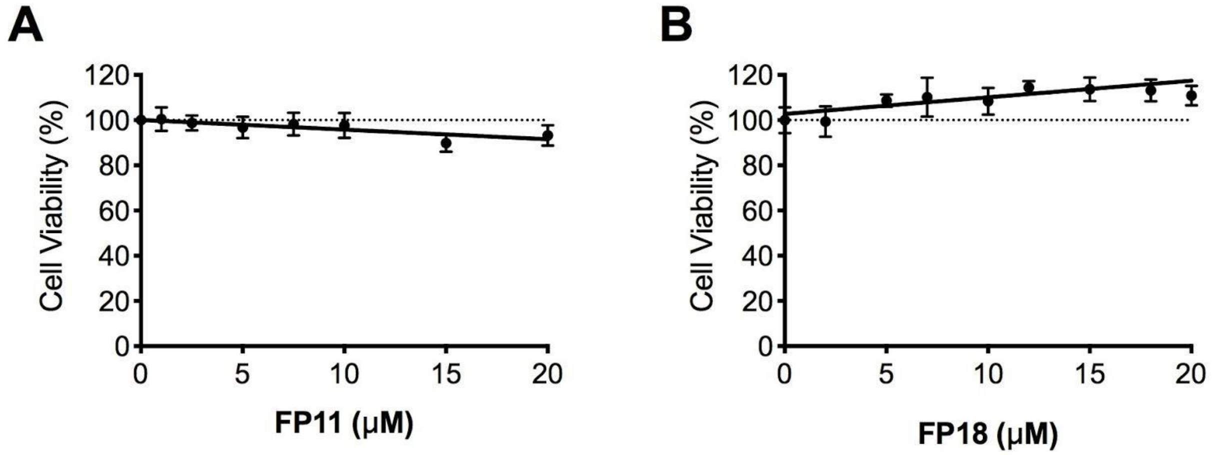

Fig. S18: THP1-derived macrophages were exposed to FP11 (A) and FP18 (B) at a concentration range of 0-20 μM. Cell viability was measured via MTT assay. Results were normalised to untreated controls and shown as mean ±SD of 5 independent samples.

Table S1.

Cell Line	Resistance	Transfected gene	Cell/well
HEK-Blue hTLR4	HEK-Blue Selection 1x	TLR4/MD-2/CD14/SEAP	4×10^4
HEK-Blue hTLR2	HEK-Blue Selection 1x	TLR2/CD14/SEAP	4×10^4
HEK-Blue Null	Zeocin (200 μg/mL)	SEAP	4×10^4

Table S1: HEK reporter cell lines used. In the table are reported the reporter cell lines used, with the corresponding antibiotic resistance, genes expression and concentration used for the experiments.

Table S2.

Compound	QPlogPo/w[a]	QPlogS[b]
FP11	10.260	-10.027
FP18	8.325	-8.765

Table S2: Log P and log S calculations**.** The values of QPlogPo/w and QPlogS parameters were computationally calculated within the QikProp tool implemented in the Maestro package (www.schroedinger.com). [a]Predicted octanol/water partition co-efficient log P. [b]Predicted aqueous solubility, log S. S in mol dm^{-3} is the concentration of the solute in a saturated solution that is in equilibrium with the crystalline solid.

Assessment of water solubility of FP11 and FP18 compounds as predicted by Qikprop. The highest log P value was obtained for FP11, indicating higher lipophilicity that might result in lower water solubility. This is in agreement with the lower log S value predicted for FP11, compared with FP18. In any case, this did not interfere with the performance of the cell assays.

References

(1) M. J. Frisch, G. W. Trucks, H. B. Schlegel, G. E. Scuseria, M. A. Robb, J. R. Cheeseman, G. Scalmani, V. Barone, B. Mennucci, G. A. Petersson, H. Nakatsuji, M. Caricato, X. Li, H. P. Hratchian, A. F. Izmaylov, J. Bloino, G. Zheng, J. L. Sonnenberg, M. Had, W. C. *Gaussian 09, Revision A.1*; 2009.

(2) Case, D. A.; Babin, V.; Berryman, J.; Betz, R. M.; Cai, Q.; Cerutti, D. S.; Cheatham, T. E., III; Darden, T. A.; Duke, R. E.; Gohlke, H.; Goetz, A. W.; Gusarov, S.; Homeyer, N.; Janowski, P.; Kaus, J.; Kolossváry, I.; Kovalenko, A.; Lee, T. S.; LeGrand, S, X. . K. *P. A. Amber 14*; 2014.

(3) Morris, G. M.; Huey, R.; Lindstrom, W.; Sanner, M. F.; Belew, R. K.; Goodsell, D. S.; Olson, A. J. AutoDock4 and AutoDockTools4: Automated Docking with Selective Receptor Flexibility. *J. Comput. Chem.* **2009**, *30* (16), 2785–2791.

(4) Trott, O.; Olson, A. J. AutoDock Vina: Improving the Speed and Accuracy of Docking with a New Scoring Function, Efficient Optimization, and Multithreading. *J. Comput. Chem.* **2009**, NA-NA.

(5) Roe, D. R.; Cheatham, T. E. PTRAJ and CPPTRAJ: Software for Processing and Analysis of Molecular Dynamics Trajectory Data. *J. Chem. Theory Comput.* **2013**, *9* (7), 3084–3095.

(6) Park, B. S.; Song, D. H.; Kim, H. M.; Choi, B.-S.; Lee, H.; Lee, J.-O. The Structural Basis of Lipopolysaccharide Recognition by the TLR4–MD-2 Complex. *Nature* **2009**, *458* (7242), 1191–1195.

(7) Ohto, U.; Fukase, K.; Miyake, K.; Shimizu, T. Structural Basis of Species-Specific Endotoxin Sensing by Innate Immune Receptor TLR4/MD-2. *Proc. Natl. Acad. Sci.* **2012**, *109* (19), 7421–7426.

(8) Kim, H. M.; Park, B. S.; Kim, J.-I.; Kim, S. E.; Lee, J.; Oh, S. C.; Enkhbayar, P.; Matsushima, N.; Lee, H.; Yoo, O. J.; Lee, J.-O. Crystal Structure of the TLR4-MD-2 Complex with Bound Endotoxin Antagonist Eritoran. *Cell* **2007**, *130* (5), 906–917.

(9) Ohto, U.; Fukase, K.; Miyake, K.; Satow, Y. Crystal Structures of Human MD-2 and Its Complex with Antiendotoxic Lipid IVa. *Science (80-.).* **2007**, *316* (5831), 1632–1634.

(10) Facchini, F. A.; Zaffaroni, L.; Minotti, A.; Rapisarda, S.; Calabrese, V.; Forcella, M.; Fusi, P.; Airoldi, C.; Ciaramelli, C.; Billod, J.-M.; Schromm, A. B.; Braun, H.; Palmer, C.; Beyaert, R.; Lapenta, F.; Jerala, R.; Pirianov, G.; Martin-Santamaria, S.; Peri, F. Structure–Activity Relationship in Monosaccharide-Based Toll-Like Receptor 4 (TLR4) Antagonists. *J. Med. Chem.* **2018**, *61* (7), 2895–2909.

(11) Scior, T.; Lozano-Aponte, J.; Figueroa-Vazquez, V.; Yunes-Rojas, J. A.; Zähringer, U.; Alexander, C. THREE-DIMENSIONAL MAPPING OF DIFFERENTIAL AMINO ACIDS OF HUMAN, MURINE, CANINE AND EQUINE TLR4/MD-2 RECEPTOR COMPLEXES CONFERRING ENDOTOXIC ACTIVATION BY LIPID A, ANTAGONISM BY ERITORAN AND SPECIES-DEPENDENT ACTIVITIES OF LIPID IVA IN THE MAMMALIAN LPS SE. *Comput. Struct. Biotechnol. J.* **2013**, *7* (9), e201305003.

(12) Paramo, T.; Piggot, T. J.; Bryant, C. E.; Bond, P. J. The Structural Basis for Endotoxin-Induced Allosteric Regulation of the Toll-like Receptor 4 (TLR4) Innate Immune Receptor. *J. Biol. Chem.* **2013**,

288 (51), 36215–36225.

(13) Nguyen, N. T.; Nguyen, T. H.; Pham, T. N. H.; Huy, N. T.; Bay, M. Van; Pham, M. Q.; Nam, P. C.; Vu, V. V.; Ngo, S. T. Autodock Vina Adopts More Accurate Binding Poses but Autodock4 Forms Better Binding Affinity. *J. Chem. Inf. Model.* **2020**, *60* (1), 204–211.
